# Longitudinal plasma phosphorylated‐tau217 and other related biomarkers in a non‐demented Alzheimer's risk‐enhanced sample

**DOI:** 10.1002/alz.14100

**Published:** 2024-07-05

**Authors:** Lianlian Du, Rebecca E. Langhough, Rachael E. Wilson, Ramiro Eduardo Rea Reyes, Bruce P. Hermann, Erin M. Jonaitis, Tobey J. Betthauser, Nathaniel A. Chin, Bradley Christian, Lauren Chaby, Andreas Jeromin, Guglielmo Di Molfetta, Wagner S. Brum, Burak Arslan, Nicholas Ashton, Kaj Blennow, Henrik Zetterberg, Sterling C. Johnson

**Affiliations:** ^1^ Wisconsin Alzheimer's Disease Research Center Department of Medicine University of Wisconsin School of Medicine and Public Health Madison Wisconsin USA; ^2^ Wisconsin Alzheimer's Institute University of Wisconsin School of Medicine and Public Health Madison Wisconsin USA; ^3^ Department of Neurology University of Wisconsin‐Madison School of Medicine and Public Health Madison Wisconsin USA; ^4^ Waisman Laboratory for Brain Imaging and Behavior University of Wisconsin‐Madison Madison Wisconsin USA; ^5^ ALZpath, Inc. Carlsbad California USA; ^6^ Department of Psychiatry and Neurochemistry Institute of Neuroscience and Physiology the Sahlgrenska Academy at the University of Gothenburg Mölndal Sweden; ^7^ Graduate Program in Biological Sciences: Biochemistry Universidade Federal do Rio Grande do Sul (UFRGS) Porto Alegre RS Brazil; ^8^ Clinical Neurochemistry Laboratory Sahlgrenska University Hospital Mölndal Sweden; ^9^ ICM Paris Brain Institute, ICM Pitie‐Salpetriere Hospital Sorbonne University Paris France; ^10^ Neurodegenerative Disorder Research Center Division of Life Sciences and Medicine and Department of Neurology Institute on Aging and Brain Disorders University of Science and Technology of China and First Affiliated Hospital of USTC Hefei Anhui China; ^11^ Department of Neurodegenerative Disease UCL Institute of Neurology London UK; ^12^ UK Dementia Research Institute at UCL London UK; ^13^ Hong Kong Center for Neurodegenerative Diseases Clear Water Bay Hong Kong China

**Keywords:** ALZpath, biomarkers, BMI, cognitive composite, plasma, preclinical Alzheimer's disease

## Abstract

**INTRODUCTION:**

Understanding longitudinal change in key plasma biomarkers will aid in detecting presymptomatic Alzheimer's disease (AD).

**METHODS:**

Serial plasma samples from 424 Wisconsin Registry for Alzheimer's Prevention participants were analyzed for phosphorylated‐tau217 (p‐tau217; ALZpath) and other AD biomarkers, to study longitudinal trajectories in relation to disease, health factors, and cognitive decline. Of the participants, 18.6% with known amyloid status were amyloid positive (A+); 97.2% were cognitively unimpaired (CU).

**RESULTS:**

In the CU, amyloid‐negative (A–) subset, plasma p‐tau217 levels increased modestly with age but were unaffected by body mass index and kidney function. In the whole sample, average p‐tau217 change rates were higher in those who were A+ (e.g., simple slopes(se) for A+ and A– at age 60 were 0.232(0.028) and 0.038(0.013))). High baseline p‐tau217 levels predicted faster preclinical cognitive decline.

**DISCUSSION:**

p‐tau217 stands out among markers for its strong association with disease and cognitive decline, indicating its potential for early AD detection and monitoring progression.

**Highlights:**

- Phosphorylated‐tau217 (p‐tau217) trajectories were significantly different in people who were known to be amyloid positive.
- Subtle age‐related trajectories were seen for all the plasma markers in amyloid‐negative cognitively unimpaired.
- Kidney function and body mass index were not associated with plasma p‐tau217 trajectories.
- Higher plasma p‐tau217 was associated with faster preclinical cognitive decline.

## 1 INTRODUCTION

Early detection of Alzheimer's disease (AD) is a research and clinical priority, especially with the emergence of potential new treatments like lecanemab^1^ and donanemab,^2^ which rely on an early molecular AD diagnosis. Blood‐based biomarkers (BBMs) have emerged as a promising avenue for accurately detecting AD proteinopathy.^3^, ^4^ Compared to positron emission tomography (PET) or cerebrospinal fluid (CSF)–based biomarkers, the scalability of BBMs including cost‐effectiveness and ease of collection,^5^, ^6^, ^7^ will make them attractive front‐line tools for assessing AD proteinopathy and progression, addressing the urgent need for early detection.^8^, ^9^

Several BBMs including phosphorylated tau (p‐tau), amyloid beta (Aβ)42/40 ratio, glial fibrillary acidic protein (GFAP), and neurofilament light (NfL) detect AD pathological changes or their downstream effects. However, (p‐tau217 has consistently shown high performance in differentiating AD from other neurodegenerative disorders^10^ and detecting AD pathology in patients with mild cognitive impairment (MCI), and has detected plaques and tangles in cognitively normal adults.^11^, ^12^, ^13^, ^14^ Its high discriminative validity, with areas under the curve (AUCs) exceeding 90%, makes it a promising tool.^3^, ^10^, ^15^, ^16^ In addition, p‐tau217 appears to exhibit an AD‐specific longitudinal trajectory, showing increases over time significantly associated with worsening cortical atrophy and declining cognitive performance in amyloid‐positive (A+) individuals.^11^, ^13^ There are multiple plasma p‐tau immunoassays currently available with different assay configurations and analytical detection. A novel single‐molecule array (Simoa) assay, ALZpath pTau217, demonstrates high accuracy in identifying abnormal Aβ and tau pathologies, comparable to CSF measures and superior to brain atrophy assessments, and significantly outperforms other putative plasma biomarkers and their optimal combinations.^3^

Although, plasma p‐tau217 has emerged as a viable candidate AD biomarker, less is known about the trajectories of plasma p‐tau217 alongside other biomarkers in late middle‐aged cognitively unimpaired (CU) individuals at baseline. Previous studies have indicated that factors such as age, sex, apolipoprotein E (*APOE*) genotype, and health risk factors including body mass index (BMI) and kidney function can influence the trajectories or accuracy of biomarkers.^17^, ^18^, ^19^, ^20^, ^21^, ^22^, ^23^ By investigating these modifiable risk factors alongside plasma biomarkers in cognitively healthy, amyloid‐negative (A–) adults, we can gain insight into potential age‐related or other modifiable changes in plasma biomarkers that separate from amyloid pathology. Our study's three aims addressed the overall goals of understanding longitudinal patterns of plasma p‐tau217 in individuals without and with evidence of brain amyloid proteinopathy (Aims 1 and 2) and investigating the clinical and research implications of elevated levels of p‐tau217 (Aim 3). Specifically, the aims were: (1) characterize plasma biomarker trajectories in CU, amyloid PET–negative healthy controls to assess potential moderators of trajectories (e.g., sex, *APOE*, or selected baseline health risk factors) and estimate within‐person variability; (2) examine these same potential modifiers of longitudinal plasma trajectories before and after adding A status determined from PET or CSF in the sample with available A status (*n* = 382); and (3) determine whether plasma p‐tau217 or other plasma biomarkers modified longitudinal cognitive trajectories across a set of outcomes representing multiple cognitive domains.

## 2 METHODS

### 2.1 Participants

Longitudinal plasma samples were selected from a subset of the Wisconsin Registry for Alzheimer's Prevention (WRAP)^24^ participants who met the following criteria: at least one ethylenediaminetetraacetic acid (EDTA) plasma sample, and non‐demented status at the time of the first eligible blood sample collection. All participants return approximately biennially for neuropsychological and health assessments. A total of 424 participants satisfied these criteria (detailed in Table 1; 399 [94.1%] had more than two plasma samples). For Aim 1, we further refined the subset to 226 A– individuals from PET scans, who remained CU across all sample collections. The remaining 198 participants, excluded from Aim 1, included 29 with cognitive impairment at one or more visits, 91 without an amyloid PET scan, 70 who were A+, and 8 reporting neurological disorders (6 with epilepsy, 1 with stroke, and 1 with multiple sclerosis). Aim 2 included all participants (*n* = 382) with assayed plasma biomarkers and at least one PET scan or CSF result if a PET scan was not available (PET: *n* = 326; CSF: *n* = 56). Aim 3 included 404 to 412 participants based on the cognitive outcome who were CU at their baseline cognitive assessment and had longitudinal cognitive assessments. A flowchart in Figure S1 details the inclusion criteria for each aim.

**TABLE 1:** Demographic and summary statistics.

	Overall (*N* = 424)	Sample with known A status (Aim 2: *N* = 382)	Amyloid neg CU subset (Aim 1: *N* = 226)
Age at first plasma, mean (SD)	61.79 (6.89)	62.01 (6.77)	60.94 (6.95)
Age at last plasma, mean (SD)	66.59 (6.86)	66.84 (6.71)	64.50 (6.95)
Years between visits, mean (SD)	2.48 (0.54)	2.48 (0.54)	2.50 (0.55)
Number of plasma samples, median [IQR]	3 [3, 3]	3 [3, 3]	3 [3, 3]
Age at last amyloid PET, mean (SD)	65.90 (6.91)	65.82 (6.90)	65.87 (6.87)
Female, n (%)	286 (67.45)	256 (67.02)	156 (69.0)
Non‐Hispanic White, n (%)	402 (94.8)	364 (95.3)	214 (94.7)
*APOE* ε4 carrier, n (%)^*^	163 (39.5)	143 (38.1)	68 (30.6)
*APOE* risk score, mean (SD)^*^	0.63 (1.07)	0.60 (1.05)	0.43 (0.97)
Education, mean (SD)	16.13 (2.66)	16.13 (2.65)	16.03 (2.69)
Baseline cognitive status, n (%)			
CU‐S	337 (79.3)	301 (78.8)	186 (82.3)
CU‐D	75 (17.6)	69 (18.1)	40 (17.7)
MCI	9 (2.1)	9 (2.4)	
Other	3 (0.7)	3 (0.8)	
Last cognitive status, n (%)			
CU‐S	367 (86.6)	331 (86.6)	206 (91.2)
CU‐D	42 (9.9)	38 (9.9)	20 (8.8)
MCI	14 (3.3)	12 (3.1)	
Dementia	1 (0.2)	1 (0.3)	
Baseline p‐tau217, pg/mL, median [IQR]	0.33 [0.24, 0.45]	0.33 [0.24, 0.45]	0.30 [0.23, 0.40]
Last p‐tau217, pg/mL, median [IQR]	0.37 [0.28, 0.59]	0.37 [0.28, 0.59]	0.34 [0.26, 0.44]
Baseline Aβ42/40, median [IQR]	0.07 [0.06, 0.08]	0.07 [0.06, 0.08]	0.07 [0.06, 0.08]
Last Aβ42/40, median [IQR]	0.07 [0.06, 0.08]	0.07 [0.06, 0.08]	0.07 [0.06, 0.08]
Baseline p‐tau181, pg/mL, median [IQR]	2.31 [1.81, 3.06]	2.32 [1.81, 3.04]	2.22 [1.75, 2.92]
Last p‐tau181, pg/mL, median [IQR]	2.41 [1.83, 3.12]	2.41 [1.84, 3.11]	2.21 [1.74, 2.85]
Baseline p‐tau231, pg/mL, median [IQR]	10.92 [8.15, 13.90]	10.94 [8.31, 13.80]	10.60 [8.08, 13.15]
Last p‐tau231, pg/mL, median [IQR]	11.50 [8.71, 14.30]	11.50 [8.78, 14.30]	10.89 [8.52, 13.40]
Baseline GFAP, pg/mL, median [IQR]	102.00 [75.33, 139.00]	102.00 [76.60, 140.75]	97.60 [75.17, 130.75]
Last GFAP, pg/mL, median [IQR]	103.50 [74.88, 144.25]	105.00 [75.30, 146.75]	101.00 [72.25, 137.75]
Baseline NfL, pg/mL, median [IQR]	15.65 [11.60, 21.72]	15.85 [12.10, 21.78]	15.00 [11.53, 20.48]
Last NfL, pg/mL, median [IQR]	19.25 [14.30, 26.02]	19.30 [14.62, 26.08]	18.40 [14.05, 25.53]
Amyloid CL, median [IQR]	3.75 [−0.70, 12.65]	3.75 [−0.70, 12.65]	2.27 [−2.18, 5.24]
Overall A+, n (%)	71 (18.6)	71 (18.6)	0

* *n* = 4 is missing in healthy control data set, *n* = 7 is missing in sample with known A status, *n* = 12 is missing in overall data set.

Abbreviation: CL, Centiloid; CU‐S, cognitively unimpaired‐stable; CU‐D, CU‐declining; MCI, mild cognitive impairment; Other, other cognitive impairment.

### 2.2 Neuropsychological assessment protocol

WRAP includes cognitive measurement at ≈2‐year intervals, and the comprehensive battery is described elsewhere.^24^ For the analyses reported herein, longitudinal cognitive performance was assessed using a three‐test modified Preclinical Alzheimer's Cognitive Composite (PACC3) after Donohue et al.^25^ and described in Jonaitis et al.^26^ The component tests included the Rey Auditory Verbal Learning Test (RAVLT; sum of trials 1–5),^27^ Logical Memory II,^28^ and the Digit Symbol Substitution test.^29^ In addition, three domain‐specific cognitive composites were also examined including executive function (EF), immediate memory, and delayed memory.^30^ The tests contributing to each composite are described in.^30^, ^31^ Finally, the Clinical Dementia Rating–Sum of Boxes (CDR‐SB) was also examined whenever available using the CDR scale^32^ itself or an analogous CDR‐SB derived from the Quick Dementia Rating Scale (QDRS^33^, ^34^).

### 2.3 Cognitive status determination

Participant cognitive categories included CU, MCI based on National Institute on Aging–Alzheimer's Association [NIA‐AA] criteria described in Albert et al.^35^), or dementia (based on criteria described in McKhann et al.^36^). Cognitive status was determined at each visit through a multi‐disciplinary consensus process as reported previously for WRAP participants.^37^ Participants who had impairments but did not meet the preceding criteria were classified as having “Other Cognitive Impairment” cognitive statuses.

**RESEARCH IN CONTEXT**

1. **Systematic review**: The authors searched PubMed using the terms: plasma biomarkers, Alzheimer's disease, and amyloid positron emission tomography (PET). Increasing studies show that plasma phosphorylated‐tau217 (p‐tau217) matches cerebrospinal fluid (CSF) biomarkers and PET imaging in identifying amyloid pathology and offering diagnostic/prognostic insights. Recently, Ashton et al. (2024) confirmed plasma p‐tau217's effectiveness in detecting amyloid and tau pathology, highlighting the need for further study on its long‐term trends and influencing factors.
2. **Interpretation**: The study identified distinct trajectories for p‐tau217 in amyloid‐positive individuals, showing its potential in early AD detection. It is notable that its trajectories were not influenced by kidney function or body mass index (BMI). The link between p‐tau217 and faster preclinical cognitive decline is especially significant.
3. **Future directions**: Future research should explore the utility of p‐tau217 across broader demographics, investigate underlying biology in AD, and assess its clinical application for early screening and monitoring.

### 2.4 PET neuroimaging

Amyloid burden was assessed with [C‐11] Pittsburgh Compound B (PiB) PET imaging. Details for PET acquisition, processing, quantification, and analysis methods have been described elsewhere.^38^, ^39^ T1‐weighted MRI were used for tissue class and anatomic segmentation using Statistical Parametric Mapping version 12 (SPM12). Amyloid burden was quantified using the average PiB distribution volume ratio (DVR, 0‐70 min dynamic scan, cerebellum gray matter reference region, *t* ^*^ = 35 min, k_2_’ = 0.149 min^−1^) across eight bilateral cortical regions of interest (ROIs^40^). Amyloid positivity (A+/–) was defined as DVR >1.19 based on the last available scan, using a previously validated threshold (Equivalent to ≈21.6 Centiloids^41^, ^42^). Based on local data^42^ and recent publications indicating a subthreshold stage of amyloid accumulation,^43^, ^44^ we used a threshold of PiB DVR <1.14 (≈14.1 CL) to identify the healthy control subset for Aim 1 analyses (i.e., CU at all visits and PET A– at all scans).

### 2.5 CSF measurements

The CSF levels of Aβ42^45^ and p‐tau181^46^ were measured at the Clinical Neurochemistry Laboratory, University of Gothenburg, Sweden. This was achieved using the Elecsys β‐Amyloid (1‐42) and Phospho‐Tau(181P) CSF electrochemiluminescence immunoassays on a fully automated cobas e 601 analyzer (Roche Diagnostics International Ltd., Rotkreuz, Switzerland). The board‐certified laboratory technicians performing these measurements were blinded to diagnostic and other clinical data. Subsequently, the last available CSF p‐tau181/Aβ42 ratio was calculated and included in this study. This ratio has been included in the study as it may represent a reliable alternative to amyloid PET^47^ and has been found to be sufficient for predicting progression in AD with very high accuracy.^48^ CSF p‐tau/Aβ42 ratio >0.038 was defined as A+ when amyloid PET was missing.^49^

### 2.6 Plasma biomarkers

Plasma samples were processed and analyzed at the University of Gothenburg Department of Psychiatry and Neurochemistry. The plasma biomarker of primary interest was p‐tau217. To measure p‐tau217, a novel commercially available assay from ALZpath (ALZpathDX, Carlsbad, CA) was used. The assay has a low limit of detection (0.0052–0.0074 pg/mL), a dynamic range of 0.007–30 pg/mL (minimal required dilution of 3), and satisfactory spike recovery (80%), along with acceptable intra‐ and inter‐run precision (0.5%–13%, 9.2%–15.7%, respectively). The assay has demonstrated good repeatability and intermediate precision in WRAP and other cohorts using three internal plasma quality control samples from the University of Gothenburg and two quality controls provided with the ALZpath assay kit.^3^ p‐tau217 was measured in duplicate, as it does provide extra strength to the experimental process.

Several other plasma biomarkers were quantified from the same aliquots, including Aβ42/40, p‐tau181, p‐tau231, GFAP, and NfL. Quantification of plasma Aβ42/40, GFAP, and NfL was performed using the commercial Neurology 4‐plex E kit (#103670, Quanterix).^3^ In addition, plasma p‐tau231 was assessed using *in‐house* Simoa assays developed at the University of Gothenburg,^50^ and plasma p‐tau181 was quantified using the commercial Advantage V2.1 kit (#104111, Quanterix).^11^

Each plasma biomarker was standardized (i.e., transformed to a *z*‐scale) relative to the AD disease‐negative healthy control subset described in Section 2.4 as follows: biomarker *z*‐score = (Observed value – Mean value of CU, PET A–)/(Standard deviation of CU, PET A–). The means (SDs) of each biomarker used for standardizing are shown in Table S1.

### 2.7 Genotyping and scoring

*APOE* genotyping was described previously.^24^ A derived *APOE* score^51^ that accounts for non‐linear AD risk from the combination of ε2, ε3, and ε4 alleles was used. The *APOE‐npscore*, a logarithmically transformed measure of *APOE* genotype's odds ratio (OR) in relation to AD neuropathology case–control status, was derived from *APOE* ε2/ε2, ε2/ε3, ε3/ε3, ε2/ε4, ε3/ε4, and ε4/ε4 genotypes, adjusted for age and sex, with negative values indicating reduced AD risk compared to ε3/ε3. The *APOE*‐npscore values for specific genotypes were as follows: ε2ε2 = −1.833, ε2ε3 = −0.916, ε3ε3 = 0, ε2ε4 = 0.904, ε3ε4 = 1.742, and ε4ε4 = 3.293.^52^

### 2.8 Health factors

A subset of participants (*n* = 411) had the following health and medical history available for their baseline plasma assessment. BMI was calculated as (kg/m^2^) using height (cm) and weight (kg). Obese was defined as a BMI ≥30 kg/m^2^ (Not obese: reference group). We also calculated the estimated glomerular filtration rate (eGFR) from serum creatinine and other clinical parameters^53^ using the 2021 Chronic Kidney Disease Epidemiology Collaboration equation (2021 CKD‐EPI^54^). CKD was defined as an eGFR below 90 mL/min/1.73 m^2^ using the described equations in accordance with guideline‐recommended GFR stages (Stage 1 [reference group]: GFR >90; Stage 2: GFR = 60–89; Stage 3: GFR = 30–59; Stage 4: GFR = 15–29; Stage 5: GFR = <15).^55^ A questionnaire assessing modifiable health and lifestyle factors of dementia, the Lifestyle for Brain Health (LIBRA) index, was created based on clinical data from physical examination or self‐reported questions,^56^ with lower scores indicating healthier lifestyle and lower lifestyle‐based dementia risk. Three LIBRA risk groups were defined based on LIBRA tertiles (low risk [reference group]: LIBRA scores between −4.2 and 0; moderate risk: LIBRA scores between 0.1 and 2.0; High risk: LIBRA scores between 2.1 and 8.1.^56^

### 2.9 Statistical analyses

Analyses were conducted in R v4.0.2 (R Core Team, 2020). Descriptive statistics of all participants with assayed plasma biomarkers and healthy control subset are presented as mean (SD) for normally distributed and median (25th, 75th percentile) for non‐normally distributed continuous data; *n* (%) for categorical variable. Spearman rank correlations were used to assess the relation between the plasma biomarkers closest to PiB PET, PiB DVR, and baseline healthy factors (BMI, CDK_EPI, LIBRA Index).

#### 2.9.1 Aim 1: Characterizing plasma biomarker trajectories in healthy controls (CU, A–)

To test the hypothesis that there is no age‐related trend and the modifiable factors are not associated with plasma biomarkers in presumably healthy controls, for each plasma biomarker *z*‐score (p‐tau217; Aβ42/40, p‐tau181, p‐tau231, GFAP, and NfL) in the CU/A– subset (*n* = 226), we examined a series of mixed‐effects models (all models included a random intercept; random slopes were retained only when significant prior to adding fixed effects). Time was modeled as age in years, centered at age 60. After identifying significant random slopes, we ran a model (base model) with age (up to cubic polynomial, if significant) as the only fixed effect and removed non‐significant (NS), highest‐order age terms sequentially. Models 1–5 added one predictor (sex, *APOE*‐npscore, BMI, CKD_EPI, LIBRA index)^*^age included to the base model; if the interaction was NS, it was removed—leaving the base model plus predictor. Model 6 incorporated significant main effects and interactions from Models 1–5; we sequentially removed NS interactions (least significant out first) until only significant interactions (and their supporting main effects) or significant main effects (if no corresponding interactions were significant) remained. We compared model fits of Models 1–6 with the base model Akaike's information criterion‐corrected (AICc) and with the model with lowest/best AICc; we report results for the best‐fitting model and any others with ∆AICc <2 for each biomarker, as these models represent similarly adequate fit.^57^ The ∆ values for any given model are linked to the evidence ratio for the best model as exp{−(1/2)Δ}.^57^ For example, if a model in question has a Δ value of 11, its evidence ratio compared to the best model is ≈245. That is, the evidence is 245 times stronger for the best model relative to the model in question. People might often judge this evidence to be very strong. Other people might choose another word; however, both judgments are based on the same quantitative evidence, an evidence ratio of 245 to 1. AICc was calculated using the “AICcmodavg” R package.^58^ The methods and results about estimating within‐person variability are in the supplement.

In this and subsequent aims, when we depicted significant interactions for each biomarker, we used simple slope estimates. Moreover, when an interaction with quadratic age was significant, we compared the simple slopes of each plasma biomarker trajectories at specific age values using the “emmeans” R package.^59^

#### 2.9.2 Aim 2: Characterizing plasma biomarker trajectories in the full baseline nondemented sample

To test the hypothesis that trajectories of plasma biomarkers would differ based on amyloid status, we again used mixed‐effects models for plasma biomarker *z*‐scores. First we ran a base model with age (up to cubic polynomial, if significant) as the only fixed effect, removed NS highest‐order age terms sequentially, and examined the same potential modifiers of longitudinal plasma trajectories as in Aim 1, using the plasma dataset with at least one available amyloid PET or CSF (*n* = 382), both before and after adjusting for A status. A+ status was categorized using PET A status (A+: DVR >1.19; ≈21.6 Equivalent Centiloids^41^, ^42^), or CSF p‐tau/Aβ42 ratio binary variable (A+: p‐tau/Aβ42 ratio >0.038) when PET A was missing. For each plasma biomarker, we ran the sets of mixed‐effects models (1–5) used in Aim 1, and we again reported the best‐fitting model (Model 6) for each biomarker and any others with ∆AICc <2. We then added A status^*^age (up to cubic polynomial, if significant) to the best‐fitting model to characterize whether and how age‐related plasma trajectories differ by PET A status across biomarkers; if the interaction and/or the main effect was NS, it was removed sequentially leaving significant interactions (and their supporting main effects) or significant main effects (if no corresponding interactions are significant) remained (Model 7). Subsequently, we compared the best‐fitting model before and after adding PET A status. (The details of the sensitivity analyses are presented in the supplement.)

#### 2.9.3 Aim 3: Plasma biomarkers and longitudinal cognitive trajectories

To test the hypothesis that a higher baseline level of plasma biomarkers is predictive of faster preclinical cognitive decline, in Aim 3 analyses, we again used mixed‐effects models to examine whether baseline levels of plasma biomarkers modified longitudinal cognitive trajectories for the following cognitive outcomes: PACC3 (primary outcome), EF, Immediate Memory, Delayed Memory and CDR‐SB in participants who were CU at their baseline plasma assessment. We first ran a base model for each cognitive outcome including random effects, age (retaining significant polynomial terms up to a cubic polynomial, centered at age 60), sex, education level (<BA vs ≥ BA), and the Wide Range Achievement Test‐III (WRAT3) standard reading score. For all but the CDR‐SB, the number of prior exposures to the cognitive outcome was included (“practice,” range = 0–7). Next, Models 1–6 started with predictor (p‐tau217; Aβ42/40, p‐tau181, p‐tau231, GFAP, and NfL)^*^age added to the base model and then removed if NS. We compared model fits of Models 1–6 with the base model AICc and with the model with lowest/best AICc; we report results for the best‐fitting model and any others with ∆AICc <2 for each biomarker as these models represent a similarly adequate fit. When we depicted significant plasma p‐tau217^*^age interaction for each cognitive outcome, we used simple slope estimates. Moreover, when an interaction with quadratic age was significant, we used model output to estimate simple slopes (and confidence intervals [CIs]) at ages 60, 65, and 70 for each cognitive outcome and plotted simple slopes using the median of values of the cutoffs using the risk‐level approach.^3^

In secondary analyses of PACC3, our primary cognitive outcome, we examined whether adding retained biomarker terms in Models 2–6 improved Model 1 (predictor = p‐tau217) fit substantially. We added terms sequentially, beginning with the biomarker terms from the best‐fitting model from Models 2–6, retaining significant interactions or main effects before comparing with Model 1 AICc. If ∆AICc showed improved model fit, we continued to the next best model from Models 2–6 and added terms from that model. Only biomarkers from models that improved model fit over the base model were considered for this process. Multicollinearity was assessed using variance inflation factors (VIFs).

## 3 RESULTS

**FIGURE 1:**
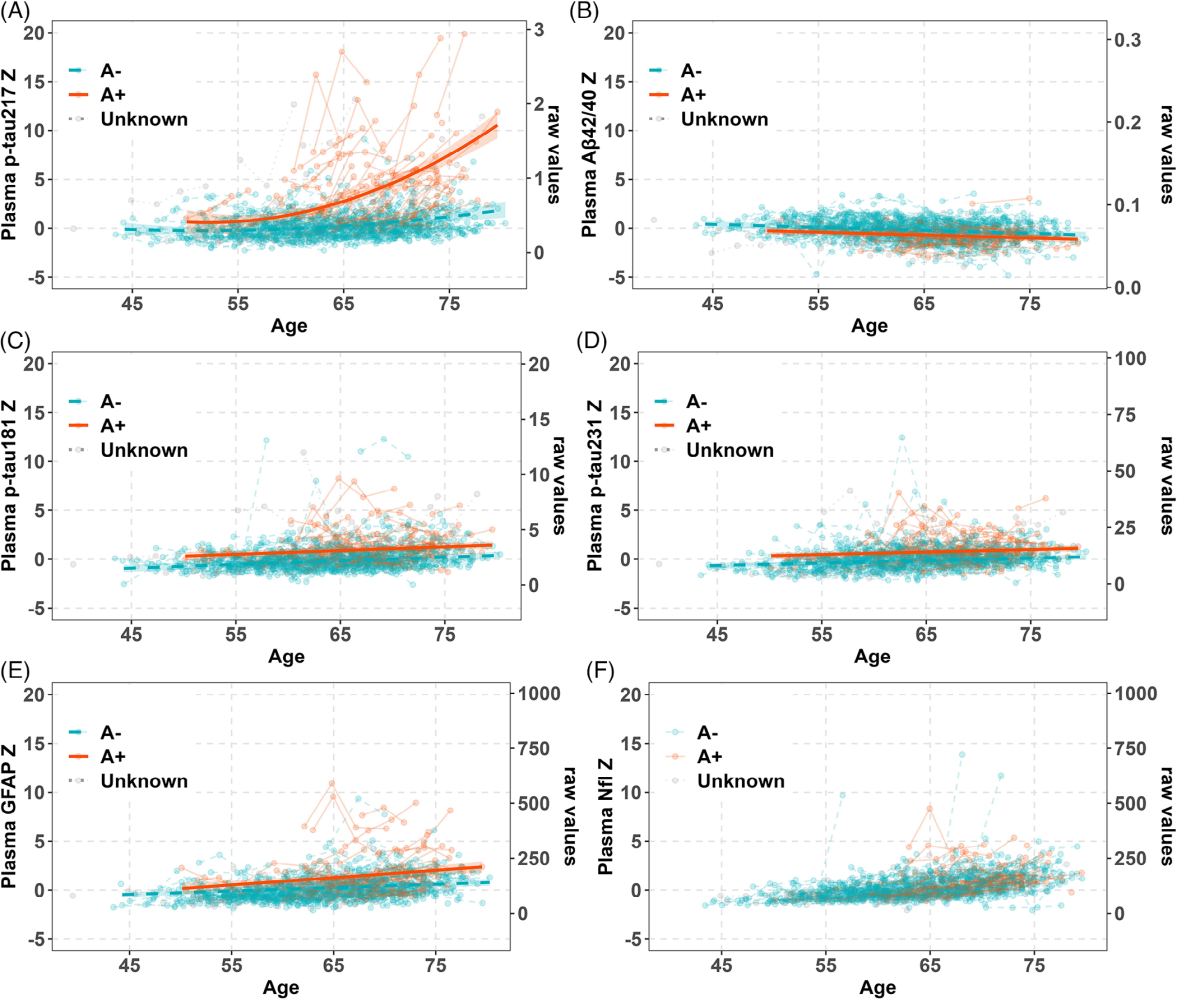
The spaghetti plot of plasma biomarkers in all available plasma samples aligned with the predicted values with A status. Amyloid positive (A+): Positron emission tomography (PET) Pittsburgh Compound B (PiB) Distribution volume ratio (DVR) >1.19 corresponding to a centiloid of 21.6, or cerebrospinal fluid (CSF) p‐tau/Aβ42 ratio >0.038 when amyloid PET was missing (*N* = 71); A‐: *N* = 311; Unknown = 42.

Table 1 shows the demographic and summary statistics for the whole sample (column 1, *n* = 424), the plasma sample with known A status based on amyloid PET or CSF (column 2 *n* = 382; Aim 2), and the healthy control subsample (column 3 *n* = 226; Aim 1). In the subset with known A status, overall, 71 of 382 (18.6%) with determinable amyloid status were classified as A+, including 65 of 326 (19.9%) with PiB PET (*n* = 326; Aim 3) and 6 of 56 (10.7%) with CSF in the absence of PiB PET. In the healthy control subset, mean (SD) age at first plasma was 60.9 (7.0) [range 43.4–76.2] with mean (SD) = 2.5 (0.6) years between the first and last plasma assessment; 68 (30.6%) were *APOE* ε4 carriers and 156 (69.0%) were female. Among all 424 people, mean (SD) age at first plasma was 0.85 years older, less female, and more *APOE* ε4 carriers than in the healthy control subsample; 412 participants (97.2%) were CU at baseline (Aim 4). Spaghetti plots of plasma biomarker *z*‐scores versus age are shown in Figure 1 (whole sample, by A +/–/unknown status, with the thicker prediction lines explained in Section 3.2.1) and Figure S2 (healthy control subsample). Figure S3 depicts pairwise Spearman correlations among the plasma and PET biomarkers. In the healthy control subsample, correlations among pairs of plasma biomarkers ranged from −0.22 to 0.65, whereas no significant correlations were observed between global PiB DVR and each plasma marker. In the whole sample, correlations among pairs of plasma biomarkers ranged from −0.28 to 0.71, whereas correlations between global PiB DVR and each plasma marker ranged from −0.31 to 0.44.

### 3.1 Aim 1 plasma trajectories in healthy control subset

In Aim 1, the healthy control subset, Figure S4 graphically represents the within‐person variability for each plasma biomarker.

#### 3.1.1 Plasma p‐tau217

**FIGURE 2:**
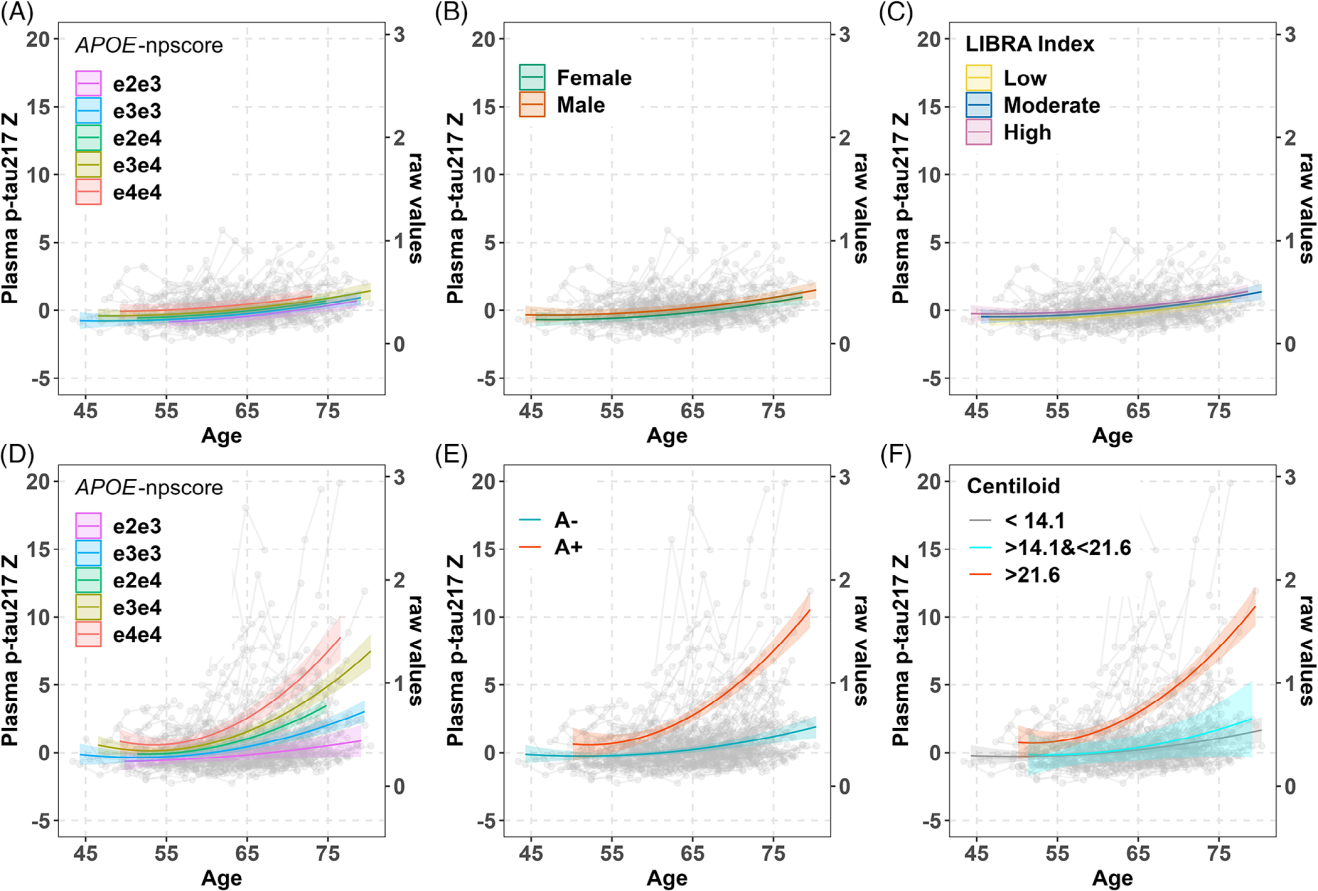
Interaction plot from mixed‐effects model of plasma p‐tau217 in cognitively unimpaired amyloid‐negative (top row) and whole sample with known A status (bottom row). Estimates come from the significant main effects or interaction effects of best‐fitting regression models for plasma p‐tau217 in healthy control (A, B, and C), in whole sample with known A status before and after adding A status to the model (D and E), and in sample with PET amyloid (F). The predicted mean plasma p‐tau217 z‐scores for plasma are on the left y‐axis, the raw values are on the right y‐axis, and age in years is represented on the x‐axis. The observed data points are shown in gray. Bands represent 95% confidence intervals. Estimates are truncated to be within the age range of participants for a particular group. The predicted age‐related trajectories of plasma p‐tau217 are observed to be higher among *APOE* ε4 carriers, male participants, and those with a higher LIBRA index within the healthy control group (A, B, and C). Across the whole sample with known A status, changes in these trajectories were associated significantly with several factors in separate mixed‐effects models (D, E, and F). Specifically, *APOE* genotype influenced the rate of change, with the fastest decline seen in ε4/ε4 carriers, followed by ε3/ε4, ε2/ε4, ε3/ε3, and ε2/ε3, in that order. In addition, amyloid status (A+ vs A–) and amyloid Centiloid values also significantly impacted p‐tau217 trajectories. The effects varied significantly across different Centiloid ranges, with those  >21.6 showing the most pronounced changes, followed by the 14.1 to 21.6 range, and the least changes observed in values,14.1.

In Aim 1 mixed‐effects analyses of the healthy CU and A– subset, linear and quadratic age terms were significant in the base model for plasma p‐tau217 (age beta (95% CI) = 0.04 (0.02–0.06), *p* < 0.001; age^2^ beta(95% CI) = 0.002(0.0002–0.003); *p* = 0.029; AICc = 1776.1; marginal *R* ^2^/Conditional *R* ^2^ = 0.090/0.764). No predictor^*^age interactions were retained in Models 1–6; Table 2). Models 1, 2, and 6 AICc values suggested a better fit (i.e., ∆AICc >2) than the age‐only base model. Model 6 (∆AICc = −10.18), for example, showed significant contributions to plasma p‐tau217 values associated with male sex, *APOE*‐npscore, and higher LIBRA index with beta (and *p*‐value), respectively, of 0.33 (0.022), 0.21 (0.003), and 0.41 (0.015); see Table 2 for additional model output for Models 1–6. Predicted age‐related trajectories for these variables are shown in Figure 2A–C. The BMI and kidney function variables were not significantly associated with slope over time or with the mean level of plasma p‐tau217.

**TABLE 2:** Plasma p‐tau217 mixed‐effects model sets output in healthy control group (Aim 1).

	1 (predictor = sex)			2 (predictor = npscore)			3 (predictor = BMI)			4 (predictor = CKD‐EPI)			5 (predictor = LIBRA)			6 (combined)		
Predictors	Estimates	CI	*p*	Estimates	CI	*p*	Estimates	CI	*p*	Estimates	CI	*p*	Estimates	CI	*p*	Estimates	CI	*p*
(Intercept)	−0.15	−0.32 to −0.02	0.076	−0.15	−0.31 to −0.00	0.054	−0.13	−0.30 to −0.04	0.137	−0.14	−0.34 to −0.06	0.178	−0.27	−0.54 to −0.00	**0.048**	−0.49	−0.78 to −0.21	**0.001**
c60age	0.04	0.02 to −0.06	**<0.001**	0.04	0.03 to −0.06	**<0.001**	0.04	0.02 to −0.06	**<0.001**	0.04	0.02 to −0.06	**<0.001**	0.04	0.02 to −0.06	**<0.001**	0.04	0.02 to −0.06	**<0.001**
c60age^2^	0.00	0.00 to −0.00	**0.030**	0.00	0.00 to −0.00	**0.034**	0.00	0.00 to −0.00	**0.031**	0.00	0.00 to −0.00	**0.026**	0.00	0.00 to −0.00	**0.028**	0.00	0.00 to −0.00	**0.033**
Male	0.31	0.02 to −0.60	**0.035**													0.33	0.05 to −0.61	**0.022**
npscore				0.19	0.06 to −0.33	**0.006**										0.21	0.07 to −0.34	**0.003**
BMI [obese]							0.22	−0.06 to −0.51	0.129									
CKD EPI [Stage 2]										0.15	−0.13 to −0.42	0.294						
CKD EPI [Stage 3]										0.35	−0.53 to 1.23	0.436						
LIBRA [Moderate]													0.20	−0.15 to −0.55	0.255	0.21	−0.13 to −0.55	0.236
LIBRA [High]													0.37	0.03 to −0.72	**0.033**	0.41	0.08 to −0.75	**0.015**
Random Effects																		
σ^2^	0.38			0.38			0.38			0.38			0.38			0.38		
τ_00_	0.70_Reggieid_			0.68_Reggieid_			0.70_Reggieid_			0.71_Reggieid_			0.70_Reggieid_			0.65_Reggieid_		
τ_11_	0.00_Reggieid.c60age_			0.00_Reggieid.c60age_			0.00_Reggieid.c60age_			0.00_Reggieid.c60age_			0.00_Reggieid.c60age_			0.00_Reggieid.c60age_		
ρ_01_	0.20_Reggieid_			0.18_Reggieid_			0.20_Reggieid_			0.23_Reggieid_			0.22_Reggieid_			0.19_Reggieid_		
ICC	0.74			0.73			0.74			0.74			0.74			0.72		
*N*	222_Reggieid_			222_Reggieid_			222_Reggieid_			222_Reggieid_			222_Reggieid_			222_Reggieid_		
Observations	665			665			665			665			665			665		
Marginal *R* ^2^/Conditional *R* ^2^	0.106/0.763			0.103/0.759			0.097/0.764			0.098/0.766			0.104/0.765			0.140/0.758		
AICc	1750.1			1747.2			1752.3			1755.1			1752.0			1742.3		
Δ AICc	−2.44			−5.37			−0.26			2.62			−0.54			−10.18		

*Note*: The base model is age fixed effects up to cubic polynomial (retaining highest order significant term and lower order terms; centered at age 60 (c60)) and random effects. Models 1–5 started with predictor^*^age included and then removed if non‐significant (NS). Model 6 brings in significant main effects and interactions from Models 1–5, removes NS interactions sequentially (least significant out first) until only significant interactions (and their supporting main effects) or significant main effects (if no corresponding interactions are significant) remain. Change in Akaike information criterion ΔAICc is calculated relative to the base model; negative numbers indicate a better fit in the expanded model. Bold values are statistically significant. npscore is the Apolipoprotein E neuropathology‐based risk score; BMI body mass index; CKD EPI chronic kidney disease epidemiology collaboration; LIBRA Lifestyle for Brainhealth Index; ICC intraclass correlation coefficient; Reggieid is the person‐level study number.

#### 3.1.2 Other plasma biomarkers

In similar analyses of the other plasma biomarkers, the highest‐order age term included in each base model was linear for Aβ42/40, p‐tau181, p‐tau231, and GFAP, and quadratic for NfL. Aβ42/40: Table S2 shows output for Models 1–6 for the outcome Aβ42/40. Model 6, the best‐fitting model (∆AICc = −8.1) showed significantly lower average values in men and higher average values in participants who were obese or had Stage 3 kidney disease. Predicted age‐related Aβ42/40 trajectories for sex, obesity status, and kidney disease are shown in Figure S5A–C. p‐tau181: Model 6 was again best fitting (Table S3) and showed higher average p‐tau181 for men, people with Stage 2 kidney disease, and high LIBRA index; predicted age‐related trajectories for these variables are shown in Figure S5D–F. p‐tau231: Model 4 (= Model 6) was best fitting for p‐tau231 and showed higher average values in participants with Stage 2 kidney disease (Table S4; Figure S5G). GFAP: Model 6 was again best fitting and showed faster average increases in those with Stage 3 kidney disease, higher average values in those with Stage 3 kidney disease, or higher *APOE*‐npscore and lower average values in men and people with obesity (Table S5; Figure S5H–K). NfL: Model 4 (= Model 6) was best fitting and indicated higher average NfL values for both Stages 2 and 3 kidney disease relative to the Stage 1 reference group (Table S6; Figure S5L).

### 3.2 Aim 2 plasma trajectories in sample with available amyloid PET or CSF

As noted in the methods, we again ran Models 1–5 in the larger sample that combined the healthy control subset with anyone else who had PET or CSF. We then added an A status^*^age interaction to each of the best‐fitting models and repeated the process of removing NS, higher‐order terms. Plasma p‐tau217 results are presented in Section 3.2.1; results for the remaining biomarkers are presented in Section 3.2.2.

#### 3.2.1 Plasma p‐Tau217

In Aim 2 mixed‐effects analyses that were anchored to A+/– status (determined from amyloid PET or CSF; *n* = 382), linear and quadratic age terms were significant in the base model for plasma p‐tau217 (age beta (95% CI) = 0.09 (0.06–0.11), *p* < 0.001; age^2^ beta (95% CI) = 0.01 (0.004–0.008); *p* < 0.001; AICc = 4000.9; marginal *R* ^2^/Conditional *R* ^2^ = 0.130/0.884). Model output for models retaining predictor^*^age (up to quadratic) is shown in Table 3 for Models 1–7. Before adding age^*^A status to the model, the most parsimonious model (Model 2) included *APOE*‐npscore^*^age interactions and showed a significantly better model fit than the base model (∆AICc = −47.9). Simple slopes analyses showed that slopes associated with each *APOE* genotype began to differ at approximately age 60, with slopes(se) of 0.039 (0.023) for e2e3, 0.073 (0.015) for e3e3, 0.107 (0.013) for e2e4, 0.138 (0.018) for e3e4, and 0.196 (0.033) for e4e4 (see simple slopes across multiple ages in Figure 2D). In Model 7, the addition of A status^*^age (linear and quadratic) significantly improved model fit and attenuated the *APOE*‐npscore^*^age interactions to NS. The *APOE*‐npscore main effect remained significant, showing higher average p‐tau217 values for higher *APOE*‐npscore values (Model 7, ∆AICc = −158.52). Predicted age trajectories are shown in Figure 2E for A+ and A–; simple slopes began to differ significantly at approximately age 60, with A+ and A– slope(se) estimates of 0.232 (0.028) and 0.038 (0.013), respectively. Sex, BMI, kidney function, and LIBRA index variables were not associated significantly with slope over time or with the mean level of plasma p‐tau217. The sensitivity analysis of CU participants at plasma baseline, along with parallel analyses of the entire plasma sample (*n* = 424), and exploratory analyses using PET‐based 3‐level A status to characterize p‐tau217 slopes in the PET A+ and A– sets are presented separately in Tables S7, S8, and S9.

**TABLE 3:** Plasma p‐tau217 *z*‐score mixed‐effects model sets output in samples with A status (Aim 2).

	1 (predictor = sex)			2 (predictor = npscore)			3 (predictor = BMI)			4 (predictor = CKD EPI)			5 (predictor = LIBRA)			6 (combined)			7 (A status)		
Predictors	Estimates	CI	*p*	Estimates	CI	*p*	Estimates	CI	*p*	Estimates	CI	*p*	Estimates	CI	*p*	Estimates	CI	*p*	Estimates	CI	*p*
(Intercept)	0.16	−0.04 to −0.37	0.122	−0.04	−0.23 to −0.16	0.709	0.22	0.02 to −0.43	**0.035**	0.08	−0.17 to −0.34	0.508	0.16	−0.16 to −0.49	0.325	−0.04	−0.23 to −0.16	0.709	−0.20	−0.38 to −0.01	**0.038**
c60age	0.09	0.06 to −0.11	**<0.001**	0.07	0.04 to −0.10	**<0.001**	0.09	0.06 to −0.11	**<0.001**	0.08	0.06 to −0.11	**<0.001**	0.09	0.06 to −0.11	**<0.001**	0.07	0.04 to −0.10	**<0.001**	0.04	0.02 to −0.07	**<0.001**
c60age^2^	0.01	0.00 to −0.01	**<0.001**	0.00	0.00 to −0.01	**<0.001**	0.01	0.00 to −0.01	**<0.001**	0.01	0.00 to −0.01	**<0.001**	0.01	0.00 to −0.01	**<0.001**	0.00	0.00 to −0.01	**<0.001**	0.00	0.00 to −0.00	**0.011**
Male	0.14	−0.21 to −0.49	0.426																		
APOE npscore				0.38	0.22 to −0.54	**<0.001**										0.38	0.22 to −0.54	**<0.001**	0.25	0.10 to −0.40	**0.001**
c60age × npscore				0.04	0.01 to −0.06	**0.002**										0.04	0.01 to −0.06	**0.002**			
c60age^2^ × npscore				0.00	0.00 to −0.00	**<0.001**										0.00	0.00 to −0.00	**<0.001**			
obese							−0.05	−0.39 to −0.29	0.758												
CKD EPI [Stage 2]										0.22	−0.11 to −0.55	0.188									
CKD EPI [Stage 3]										0.08	−1.25 to −1.42	0.902									
LIBRA [Moderate]													0.04	−0.38 to −0.47	0.837						
LIBRA [High]													0.07	−0.34 to −0.48	0.744						
A+																			1.44	0.96 to −1.91	**<0.001**
c60age × A+																			0.16	0.09 to −0.24	**<0.001**
c60age^2^ × A+																			0.01	0.01 to −0.02	**<0.001**
Random effects																					
σ^2^	0.82			0.80			0.82			0.82			0.82			0.80			0.80		
τ_00_	1.79 _Reggieid_			1.67_Reggieid_			1.80_Reggieid_			1.80_Reggieid_			1.80_Reggieid_			1.67_Reggieid_			1.30_Reggieid_		
τ_11_	0.03 _Reggieid.c60age_			0.03_Reggieid.c60age_			0.03_Reggieid.c60age_			0.03_Reggieid.c60age_			0.03_Reggieid.c60age_			0.03_Reggieid.c60age_			0.02_Reggieid.c60age_		
ρ_01_	0.75 _Reggieid_			0.76_Reggieid_			0.75_Reggieid_			0.76_Reggieid_			0.75_Reggieid_			0.76_Reggieid_			0.70_Reggieid_		
ICC	0.87			0.86			0.87			0.87			0.87			0.86			0.81		
*N*	374 _Reggieid_			374_Reggieid_			374_Reggieid_			374_Reggieid_			374_Reggieid_			374_Reggieid_			374_Reggieid_		
Observations	1112			1112			1112			1112			1112			1112			1112		
Marginal *R* ^2^/Conditional *R* ^2^	0.131/0.885			0.225/0.889			0.130/0.884			0.134/0.884			0.130/0.884			0.225/0.889			0.446/0.894		
AICc	4002.3			3953.0			4002.8			4003.2			4004.9			3953.0			3842.4		
Δ AICc	1.40			−47.90			1.93			2.33			3.95			−47.90			−158.52		

*Note*: The base model is age fixed effects up to cubic polynomial (retaining highest order significant term and lower order terms; centered at age 60 (c60)) and random effects. Models 1–5 started with predictor^*^age included and then removed if non‐significant (NS). Model 6 brings in significant main effects and interactions from Models 1–5, removes NS interactions sequentially (least significant out first) until only significant interactions (and their supporting main effects) or significant main effects (if no corresponding interactions are significant) remain. Model 7 added age^*^A status up to cubic polynomial age interaction to Model 6 and removed NS interactions sequentially until only significant interactions (and their supporting main effects) or significant main effects. The change in Akaike information criterion ΔAICc is calculated relative to the base model; negative numbers indicate a better fit in the expanded model. Bold values are statistically significant. BMI is body mass index; CKD EPI chronic kidney disease epidemiology collaboration; LIBRA lifestyle for brainhealth index; ICC intra‐class correlation coefficient; Reggieid is the person‐level study number.

#### 3.2.2 Other plasma biomarkers

In similar analyses of the other plasma biomarkers, the highest order age term included in each base model was linear for all the other plasma biomarkers, and Model 7 including A status is the best‐fitting model for all plasma biomarkers (range ∆AICc = −76.43 to −37.76) except NfL. Aβ42/40: Table S10 shows output for Models 1–7 for the outcome Aβ42/40. Model 7 showed significantly lower average values in men, faster average decrease in higher *APOE*‐npscore, lower average values in those with higher *APOE*‐npscore, faster average decrease in those with Stage 3 kidney disease, and lower average values in A+. Predicted age‐related trajectories for sex, *APOE*‐npscore, kidney disease, and A status is shown in Figure S6A–D. p‐tau181: Model 7 (Table S11) showed higher average p‐tau181 for people with moderate and high LIBRA index and A+ status (relative to each low LIBRA, A–, respectively); predicted age‐related trajectories for these variables are shown in Figure S6E,F. p‐tau231: Model 7 showed higher average values in those with Stage 2 kidney disease and A+ status (Table S12; Figure S6G,H). GFAP: Model 7 showed lower average values in men or those who were obese, faster average increases in those Stage 3 kidney disease and with A+ status, higher average values in those with Stages 2 and 3 kidney disease or A+ status (Table S13; Figure S6I–L). NfL: Model 6 was best‐fitting and indicated lower average values in people with obesity, and higher average NfL values for both Stages 2 and 3 kidney disease relative to the Stage 1 reference group (Table S14; Figure S6M,N).

### 3.3 Aim 3 plasma biomarkers and cognitive trajectories

Finally, we tested whether plasma biomarkers were significantly associated with cognitive decline as measured by PACC3 (primary) and EF, Immediate Memory, Delayed Memory, and CDR‐SB (secondary) among late‐middle‐aged, initially unimpaired participants (*n* = 412). The mean average follow‐up was 9.8 (SD = 2.9) years.

#### 3.3.1 PACC3 (primary cognitive outcome) results

**FIGURE 3:**
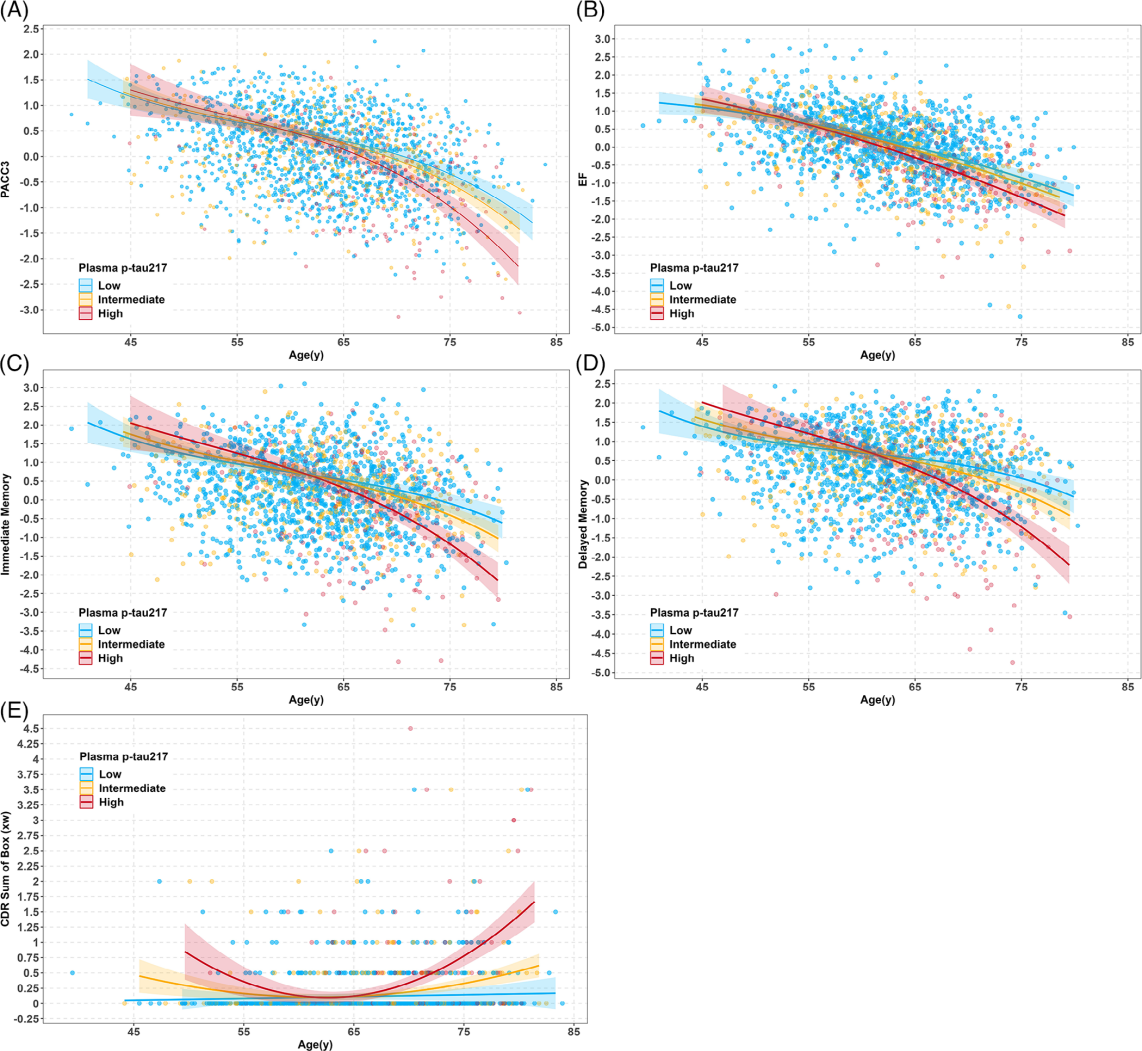
Interaction plot from mixed‐effects model of cognitive composite scores for plasma p‐tau217 (pg/mL). p‐tau217 <0.4, Low, 0.4 ≤ p‐tau217 ≤ 0.63, Intermediate, > 0.63 High risk groups. The lines represent the median plasma p‐tau217 values at baseline PACC3 (A), EF (B), Immediate memory (C), Delayed memory (D) and CDR‐SB (E) in the low, intermediate and high p‐tau217 risk groups described previously (Ashton et al.^3^). People with higher p‐tau217 values have higher rates of decline at older ages compared to people with lower p‐tau217 values.

Linear, quadratic, and cubic age terms were significant in the base model for PACC3 (age beta (95% CI) = −0.04 (−0.05 to −0.03), *p* < 0.001; age^2^ beta (95% CI) = −0.0007 (−0.001 to −0.0002); *p* = 0.005; age^3^ beta (95% CI) = −0.00008 (−0.0001 to −0.00005); *p* < 0.001; AICc = 2632.9; marginal *R* ^2^/Conditional *R* ^2^ = 0.331/0.841). Table 4 summarizes model output examining the addition of individual plasma biomarker^*^age interactions and corresponding model fits (after removing NS higher‐order interactions). All plasma biomarkers except NfL were significantly associated with longitudinal PACC3 changes. However, Models 2 (predictor = Aβ42/40) and 4 (predictor = p‐tau231) had ∆AICc <2 with the base model, suggesting a model fit similar to that of covariates and age‐only base model. The best‐fitting model was Model 1 (p‐tau217), showing significant interactions between p‐tau217 and linear and quadratic age terms. Interaction effects from Model 1 (best fitting) are depicted in Figure 3 for values representing median plasma p‐tau217 at baseline PACC3 in the low, intermediate, and high p‐tau217 risk groups described previously.^3^ People with higher p‐tau217 values have higher rates of decline at older ages compared to people with lower p‐tau217 values. Simple slopes differed significantly at age 60 and beyond (see also Figure S7 and Table S15). Models 3 (predictor = p‐tau181) and 5 (predictor = GFAP) also retained significant biomarker^*^age interactions and fit better than the base model but not as well as Model 1.

**TABLE 4:** PACC3 mixed‐effects output (Aim 3).

	1(predictor = p‐tau217)			2(predictor = Aβ42/40)			3(predictor = p‐tau181)			4(predictor = p‐tau231)			5(predictor = GFAP)			6(predictor = Nfl)		
Predictors	Estimates	CI	*p*	Estimates	CI	*p*	Estimates	CI	*p*	Estimates	CI	*p*	Estimates	CI	*p*	Estimates	CI	*p*
Intercept	0.00	−0.14 to −0.14	0.978	−0.01	−0.15 to −0.12	0.838	−0.00	−0.14 to −0.13	0.944	−0.01	−0.15 to −0.13	0.921	−0.01	−0.15 to −0.13	0.883	−0.00	−0.14 to −0.14	0.989
Male	−0.52	−0.64 to −0.39	**<0.001**	−0.53	−0.65 to −0.40	**<0.001**	−0.51	−0.63 to −0.39	**<0.001**	−0.53	−0.65 to −0.40	**<0.001**	−0.52	−0.65 to −0.40	**<0.001**	−0.53	−0.65 to −0.41	**<0.001**
No BA	−0.20	−0.33 to −0.06	**0.005**	−0.19	−0.33 to −0.05	**0.008**	−0.19	−0.33 to −0.06	**0.006**	−0.19	−0.33 to −0.05	**0.006**	−0.19	−0.33 to −0.05	**0.007**	−0.20	−0.34 to −0.06	**0.006**
Practice	0.09	0.07 to −0.12	**<0.001**	0.10	0.07 to −0.12	**<0.001**	0.09	0.07 to −0.12	**<0.001**	0.10	0.07 to −0.12	**<0.001**	0.10	0.07 to −0.12	**<0.001**	0.09	0.06 to −0.12	**<0.001**
WRAT3	0.02	0.01 to −0.03	**<0.001**	0.02	0.01 to −0.03	**<0.001**	0.02	0.01 to −0.03	**<0.001**	0.02	0.01 to −0.03	**<0.001**	0.02	0.01 to −0.03	**<0.001**	0.02	0.01 to −0.03	**<0.001**
c60age	−0.04	−0.05 to −0.03	**<0.001**	−0.04	−0.05 to −0.03	**<0.001**	−0.04	−0.05 to −0.03	**<0.001**	−0.04	−0.05 to −0.03	**<0.001**	−0.04	−0.05 to −0.03	**<0.001**	−0.04	−0.05 to −0.03	**<0.001**
c60age^2^	−0.00	−0.00 to −0.00	0.077	−0.00	−0.00 to −0.00	**0.015**	−0.00	−0.00 to −0.00	**0.037**	−0.00	−0.00 to −0.00	**0.029**	−0.00	−0.00 to −0.00	**0.015**	−0.00	−0.00 to −0.00	**0.005**
c60age^3^	−0.00	−0.00 to −0.00	**<0.001**	−0.00	−0.00 to −0.00	**<0.001**	−0.00	−0.00 to −0.00	**<0.001**	−0.00	−0.00 to −0.00	**<0.001**	−0.00	−0.00 to −0.00	**0.009**	−0.00	−0.00 to −0.00	**<0.001**
*z* p‐tau217	−0.00	−0.03 to −0.03	**0.875**															
c60age × *z* p‐tau217	−0.01	−0.01 to −0.00	**0.002**															
*z* p‐tau217 × c60age^2^	−0.00	−0.00 to −0.00	**0.041**															
*z* ab4240				−0.01	−0.07 to −0.05	0.739												
c60age × *z* ab4240				0.01	0.00 to −0.01	**0.016**												
*z* p‐tau181							−0.03	−0.07 to −0.01	0.199									
c60age × *z* p‐tau181							−0.01	−0.01 to −0.00	**<0.001**									
*z* p‐tau231										−0.00	−0.05 to −0.05	0.914						
c60age × *z* p‐tau231										−0.00	−0.01 to −0.00	**0.034**						
*z* GFAP													0.02	−0.03 to −0.08	0.356			
c60age × *z* GFAP													−0.00	−0.01 to −0.00	0.544			
*z* GFAP × c60age^2^													−0.00	−0.00 to −0.00	**0.001**			
*z* NfL																−0.02	−0.09 to −0.04	0.464
Random effects																		
σ^2^	0.11			0.11			0.11			0.11			0.11			0.11		
τ_00_	0.29_Reggieid_			0.29_Reggieid_			0.28_Reggieid_			0.29_Reggieid_			0.29_Reggieid_			0.28_Reggieid_		
τ_11_	0.00_Reggieid.c60age_			0.00_Reggieid.c60age_			0.00_Reggieid.c60age_			0.00_Reggieid.c60age_			0.00_Reggieid.c60age_			0.00_Reggieid.c60age_		
ρ_01_	−0.00_Reggieid_			−0.00_Reggieid_			−0.04_Reggieid_			−0.02_Reggieid_			0.02_Reggieid_			−0.01_Reggieid_		
ICC	0.76			0.76			0.76			0.76			0.76			0.76		
*N*	412_Reggieid_			412_Reggieid_			408_Reggieid_			412_Reggieid_			412_Reggieid_			412_Reggieid_		
Observations	1973			1973			1951			1973			1973			1973		
Marginal *R* ^2^/conditional *R* ^2^	0.352/0.842			0.333/0.842			0.349/0.841			0.333/0.841			0.339/0.841			0.332/0.841		
AICc	2600.4			2631.2			2594.6^a^			2632.2			2609.6			2634.4		
Δ AICc	−32.5			−1.73			−13.5			−0.66			−23.3			1.48		

*Notes*: Base model includes the age fixed effects up to cubic polynomial (retaining highest order significant term and lower order terms; centered at age 60 (c60)), random effects and covariates: sex, practice, education (BA bachelors degree), Wide Range Achievement Test‐III (WRAT3) reading score. The change in Akaike information criterion Δ AICc is calculated relative to the base model; negative numbers indicate a better fit in the expanded model. Bold values are statistically significant. ICC is the intraclass correlation coefficient; Reggieid is the person‐level study number.

^a^ There are four missing baseline p‐tau181. The smallest AICc is Model 1, 2575.9 (ΔAICc = −32.3); followed by Model 5, 2584.7 (ΔAICc = −23.5); and Model 3, 2594.6 (ΔAICc = −13.5) when we compared the model results in the same data sets.

In secondary analyses of PACC3, Model 7, the addition of GFAP^*^age (linear and quadratic) significantly improved model fit and attenuated the p‐tau217^*^quadratic age interactions to NS. The p‐tau217^*^linear age remained significant, showing higher average p‐tau217 values for higher rates of decline (Model 7, ∆AICc = −9.2; see Table S16). GFAP accounted for variability in PACC3 in addition to plasma p‐tau217.

#### 3.3.2 Secondary cognitive outcomes results

Parallel analyses of Models 1–6 for the secondary cognitive outcomes showed results very similar to PACC3, and p‐tau217 was significantly associated with changes in all longitudinal cognitive outcomes. Specifically, Model 1 (predictor = p‐tau217) was the best‐fitting model for each secondary cognitive outcome (ΔAICc = −13.13 for EF; ΔAICc = −49.8 for immediate memory; ΔAICc = −63.97 for delayed memory; ΔAICc = −48.67 for CDR‐SB). Each model included a significant p‐tau217^*^age interaction (EF) or significant p‐tau217^*^ age^2^ interaction (the remaining cognitive outcomes). Interaction effects from Model 1 (best fitting) for each secondary cognitive outcome are depicted in Figure 3B–E. Simple slopes differed significantly at age 60 and beyond for each cognitive outcome (see also Figure S7 and Table S15). For additional details on the model output for Models 1–6 of each cognitive outcome, refer to Tables S17–S20.

## 4 DISCUSSION

The discriminative accuracy of the ALZpath pTau217 assay, as recently described by Ashton et al.^3^ using WRAP showed a high AUC of 0.93 against amyloid PET, and a similar AUC for tau PET at a higher concentration cutoff. Comparable AUCs were reported in two other older, more impaired cohorts, validating the robustness of p‐tau217 as a biomarker.^3^ In contemporary medical practice, securing a high‐confidence etiological diagnosis of AD increasingly depends on the synergistic use of detailed clinical assessments and AD‐specific biomarkers. BBMs are particularly promising due to their potential for non‐invasive, cost‐effective application in clinical settings beyond the confines of specialized research studies and memory clinics. There is significant ongoing research and investment in developing the “best” blood‐based AD biomarker. Like other fluid and imaging biomarkers, existing blood‐based measures are not perfect when used in isolation and require contextual interpretation, which includes accounting for sex differences, genetic factors, and health risk factors. Our study contributes to this body of knowledge with a comprehensive analysis of longitudinal plasma samples from predominantly CU participants. Although most studies on plasma p‐tau217 are cross‐sectional, our longitudinal approach is crucial for enhancing our understanding of this biomarker's characteristics. We provide insights into the use of plasma p‐tau217 for early detection and continuous monitoring of AD, especially during its preclinical stages. Our goal in the present study was to gain a greater understanding of longitudinal p‐tau217 change and of factors that might be related to such change, and to compare the performance of this marker to several other plasma Simoa‐based markers of AD proteinopathy or its presumed downstream effects.

There were several informative findings. Among unimpaired people without PET evidence of Aβ, subtle age‐related trajectories were seen for all the BBMs, including p‐tau217; intra‐individual variability among non‐AD CU was clearly less for Aβ42/40 than the other markers, whereas all the other assays showed relatively similar coefficients of variation. In the larger sample including people who were A+ via amyloid PET or CSF, the trends over time were pronounced and AD disease related for p‐tau217 and GFAP. The slope difference of p‐tau217 for the A+ versus the A– group was the largest. *APOE‐*npscore and amyloid status were strongly related to p‐tau217 trajectories, whereas potential confounding factors of BMI and estimated kidney function and LIBRA index were not. These same confounding factors were variously associated with mean levels or trends over time in the other plasma markers. Furthermore, these findings were consistent across various analyses, including those ignoring amyloid status and exploratory analyses using a three‐level PET amyloid status. As we and other groups have observed with other p‐tau217 markers, baseline p‐tau217 levels were strongly associated with cognitive decline, indicating that individuals with higher plasma concentrations of p‐tau217 exhibited the greatest cognitive deterioration. This research contributes significantly to the ongoing development of blood‐based AD biomarkers, providing crucial insights into their application for early detection and monitoring of AD. We will continue to discuss these novel findings and their broader impact in subsequent sections.

### 4.1 Aim 1: Longitudinal plasma trajectories in amyloid‐negative CU controls

The Aim 1 focus on A– CU was intended to elucidate and compare non‐disease sources of fluctuation. All the markers, including p‐tau217, showed a modest but significant age‐related upward slope in this presumed non‐disease subset, although we cannot rule out that some of this would have been disease related but not yet detectable at the designated amyloid PET threshold. In fact, the *APOE‐*npscore, sex, and higher LIBRA index were associated with p‐tau217 concentration levels, although these were main effects that did not vary with time and did not explain additional variance in change over time beyond the base model involving age. It is important to note that the BMI and kidney function components were each unrelated to p‐tau217 change in this non‐disease subset, suggesting that the plasma p‐tau217 concentrations are valid within the ranges of BMI and kidney function that were assessable in this relatively healthy group.

In parallel analyses of other plasma biomarkers (Aβ42/40, p‐tau181, p‐tau231, GFAP, NfL) in this group, no consistent significant interactions were found between modifiable factors (LIBRA index, BMI, kidney function) and age on biomarker levels, except for a single interaction between kidney function and age on GFAP. These findings suggest that AD analyte measurements are largely unaffected by health and lifestyle factors in this group. This inference is of course limited to the variables and variance in this healthy, amyloid‐negative, CU control group.

Main effects were mixed: BMI correlated with lower Aβ42/40 (AD‐like) but also lower GFAP (less disease‐like). Higher chronic kidney disease was linked to increased Aβ42/40, p‐tau181, p‐tau231, GFAP, and NfL levels. A higher (less healthy) LIBRA index correlated with higher p‐tau181 levels but was stable over time.

### 4.2 Aim 2: Longitudinal plasma trajectories in samples with available amyloid PET or CSF

In Aim 2, we found that the trajectories of plasma p‐tau217 differed significantly based on amyloid status, which suggests distinct stages in AD progression for amyloid‐positive versus amyloid‐negative individuals. Before adding amyloid status in the model, *APOE* modified the trajectories over time for p‐tau217 and Aβ42/40; associate mean levels of p‐tau181, p‐tau231, and GFAP; but no association with NfL. For p‐tau217, the simple slopes by *APOE*‐npscore began to diverge at around age 60. These results make sense because *APOE* genotype is highly predictive of amyloid. Sex, BMI, kidney function, and LIBRA index did not influence the p‐tau217 longitudinal trajectories, which further raises confidence that this assay will be useful across these factors. Consistent with prior reports, kidney disease impacted Aβ42/40 trajectories.^17^, ^60^ Adding amyloid status significantly improved the model (ΔAICc = −158.52), with A+ status explaining most variance in p‐tau217 and altering GFAP trajectories. For p‐tau217, simple slopes differed significantly at age 60 and after for PET A+ versus A–. After incorporating A+ status, *APOE* was associated only with p‐tau217 mean levels. Amyloid status associated with the mean levels of Aβ42/40, p‐tau181, and p‐tau231. Plasma NfL was not associated with amyloid status, which was consistent with a recent study,^61^ although others have shown that higher NfL levels are associated with PET measurements of Aβ plaque.^62^

### 4.3 Aim 3: Association with longitudinal cognitive trajectories

In this study, we observed significant associations between various plasma biomarkers and cognitive decline, with p‐tau217 (ALZpath) exhibiting the strongest correlations across all cognitive outcomes over a median follow‐up of 10 years. Across the initially unimpaired sample, the cognitive trajectories of biomarker low, intermediate, and high groupings began to diverge at around age 60. Previous studies have found that plasma p‐tau217 with different assays predicted cognitive decline in preclinical AD.^13^, ^63^ It is noteworthy that GFAP accounted for variability in PACC3 in addition to plasma p‐tau217. This suggests that GFAP, which is a non‐specific marker of neurodegeneration, may capture certain aspects of Alzheimer's pathology downstream effect that p‐tau217 alone does not, providing a more nuanced understanding of biomarker dynamics in preclinical AD. Our findings extend the current literature by demonstrating the early elevation of GFAP levels, even before Aβ‐PET positivity becomes apparent. This aligns with recent studies suggesting GFAP's potential in early disease detection,^64^, ^65^ but goes further by quantifying its impact across multiple cognitive outcomes. This contrasts with Mattsson‐Carlgren et al.,^63^ who did not observe added value for GFAP beyond p‐tau217 in a cohort of older, Aβ‐positive CU participants. The differences are largely attributable to study design, as the present study included both amyloid‐positive and amyloid‐negative participants and a wider age range. Furthermore, our results highlight the potential of using a panel of plasma biomarkers for the early detection and monitoring of AD. By incorporating measures such as the Aβ42/40 ratio, p‐tau181, p‐tau231, and GFAP, we can achieve a more comprehensive assessment of disease progression. This approach is particularly critical in light of our findings that higher p‐tau217 levels correlate with accelerated cognitive decline, thereby reinforcing the utility of p‐tau217 as a powerful predictor of preclinical AD progression.

### 4.4 Limitations and future directions

Study limitations include the following. WRAP is a volunteer cohort with an over‐sampling of participants with a parental history of AD and is non–population‐based; these factors limit the generalizability of our findings. Furthermore, the sample overlaps with that in prior reports.^3^ As such, replication of this method in different cohorts is needed to determine to what extent this approach is generalizable. Another limitation is the potential for type I errors due to multiple comparisons, which may inflate the likelihood of finding significant results by chance. Given the high accuracy of plasma p‐tau217 compared to tau PET, our upcoming research will provide a comprehensive analysis of the relationship between plasma p‐tau217 and tau PET. Future studies will also assess the benefits of incorporating plasma p‐tau217 into a trial screening workflow to inform clinical trial design.

## 5 CONCLUSIONS

Our findings demonstrate subtle but detectable age‐related trajectories for all plasma markers including p‐tau217 in the healthy control sample. In the larger sample, the trends over time were pronounced and AD disease related. *APOE‐npscore*s and amyloid PET status modified p‐tau217 trajectories, whereas potential confounding factors of BMI and kidney function did not. These same confounding factors were associated with time changes in the other plasma markers. Similarly, we demonstrated that people with higher plasma concentrations of p‐tau217 exhibited the greatest cognitive decline across multiple cognitive outcomes.

## CONFLICT OF INTEREST STATEMENT

In the past 3 years, S.C.J. has served as consultant to Enigma, Prothena, and ALZpath. H.Z. has served at scientific advisory boards and/or as a consultant for Abbvie, Acumen, Alector, Alzinova, ALZPath, Amylyx, Annexon, Apellis, Artery Therapeutics, AZTherapies, Cognito Therapeutics, CogRx, Denali, Eisai, Merry Life, Nervgen, Novo Nordisk, Optoceutics, Passage Bio, Pinteon Therapeutics, Prothena, Red Abbey Labs, reMYND, Roche, Samumed, Siemens Healthineers, Triplet Therapeutics, and Wave; has given lectures in symposia sponsored by Alzecure, Biogen, Cellectricon, Fujirebio, Lilly, Novo Nordisk, and Roche; and is a co‐founder of Brain Biomarker Solutions in Gothenburg AB (BBS), which is a part of the GU Ventures Incubator Program (outside submitted work). K.B. has served as a consultant and at advisory boards for AC Immune, Acumen, ALZPath, AriBio, BioArctic, Biogen, Eisai, Lilly, Moleac Pte. Ltd, Novartis, Ono Pharma, Prothena, Roche Diagnostics, and Siemens Healthineers; has served on data monitoring committees for Julius Clinical and Novartis; has given lectures, produced educational materials, and participated in educational programs for AC Immune, Biogen, Celdara Medical, Eisai, and Roche Diagnostics; and is a co‐founder of Brain Biomarker Solutions in Gothenburg AB (BBS), which is a part of the GU Ventures Incubator Program, outside the work presented in this paper. All other authors declare that they have no conflicts of interest to disclose. Author disclosures are available in the supporting information.

## CONSENT STATEMENT

The study procedures received approval from the University of Wisconsin‐Madison Institutional Review Board and were conducted in compliance with the World Medical Association Declaration of Helsinki. All subjects provided informed consent.

## Supporting information

Supporting Information

Supporting Information
