# Supplementary material for: Longitudinal plasma phosphorylated‐tau217 and other related biomarkers in a non‐demented Alzheimer's risk‐enhanced sample
Source: Alzheimers Dement. 2024 Jul 5;20(9):6183–204. doi: 10.1002/alz.14100 (PMC11497664; doi:10.1002/alz.14100)
Supplement: Supplementary file 1 — Supporting Information [file ALZ-20-6183-s001.docx]

**Supplementary Online Content**

***Supplementary Methods***

**eStatistical analyses**

**e.1 Aim 1: characterizing plasma biomarker trajectories in healthy controls (CU, A-)**

Within-person variability in stable, amyloid-PET-negative people is needed in this rapidly developing area of AD diagnostics, where specific biomarkers and assays are being considered for clinical implementation and therapeutic trial use. To test the hypothesis that significant variability might undermine the utility of p-tau217 in clinical settings, we examined the within-subject variability over time for each plasma biomarkers using coefficient of variation (CV_I_), which was estimated with CV-ANOVA, the “Røraas method,” a validated and recommended ANOVA method for estimating CV_I_ (Røraas et al. 2016; Brum, Ashton, et al. 2023). Age was included in the model to adjust for the trend.

**e.2 Aim 2: characterizing plasma biomarker trajectories in the full baseline non-demented sample**

To test the hypothesis the trajectories of plasma biomarkers would differ based on amyloid status, for Aim 2 analyses characterizing longitudinal trajectories of plasma biomarkers by amyloid status, we ran sensitivity analyses and exploratory analyses.

In the first sensitivity analyses, we omitted those who had MCI at plasma baseline (n=9) and reran the mixed effect models on plasma p-tau217. In another sensitivity analyses adding in those with plasma but not PET or CSF (n=42), we reran the best-fitting model from base or model 1-6 above for each plasma biomarker using the full set who were non-demented at baseline plasma samples (including all available plasma data, n=424). We then reported whether the patterns hold or change.

In exploratory analyses reflecting the lower PET A- threshold used for ‘healthy controls’ in Aim 1, we reran the best fitting model from model 6 above including 3 level PET A status (DVR<1.14, 1.14-1.19, and >1.19) instead of 2 level PET A status using the subset of 326 with A status determined from amyloid PET data, and reported main effect or simple slope comparisons among these groups.

***Supplementary Results***

**e.1 Aim 1** **plasma trajectories in healthy control subset**

In Aim 1 mixed effects analyses of the healthy control subset, supplementary Figure 4 graphically represents the CV_I_ values and their associated 95% CIs. Plasma Aβ42/40 ratio demonstrated the lowest CV_I_ among all evaluated biomarkers (8.1%, 95% CI: 7.7–8.6), and all the other assays showed higher and relatively similar coefficients of variation; the CV_I_ (and 95% CI) for plasma p-tau217 was 19.8% (18.7-20.8). All these values highlight the utility of plasma biomarkers including p-tau217 in clinical settings.

**e.2 Aim 2 plasma trajectories in sample who have available Amyloid PET or CSF**

As noted in the emethods, in sensitivity analyses, we and reran the mixed effect models on plasma p-tau217 in those who were CU at plasma baseline (n=9), and then rerun the best fitting model before adding A status to the model above for each biomarker in full plasma sample. Last, in exploratory analyses connecting the Aim 1 healthy control subset with all with amyloid PET, we repeated these analyses with the subset with amyloid PET, partitioning those below the A+ threshold into <1.14 (healthy control) and 1.14-1.19. Plasma p-tau217 results for these analyses are presented in e.2.1; results for the remaining biomarkers are presented in e.2.2.

**e.2.1 Plasma p-Tau217**

In Aim 2 sensitivity analyses, we omitted those who had MCI at plasma baseline (n=9) and reran the mixed effect models on plasma p-tau217. Overall patterns of significance remained the same and parameter estimates appeared stable (see supplementary table 7).

In Aim 2 parallel analyses of the whole plasma sample (n=424), the model retained the same variables as the best-fitting model (Model 2) above and beta estimates were very similar (sensitivity analysis output in column 1 of Supplementary Table 8).

In exploratory analysis with PET-based 3-level A status analysis to characterize pTau217 slopes in the PET A+ set and the A- set partitioned into those in the healthy control (low PiB DVR) subset vs those with intermediate PiB DVR, the best-fitting model included A group* age^2^ (and lower order related terms) and *APOE*-npscore (column 1 in Supplementary Table 9). Predicted age trajectories are shown for each amyloid group in Figure 2F; the average pTau217 age trajectories of the two lower amyloid groups do not differ significantly, while the simple slopes of the A+ group differ significantly from the low group at approximately age 60 and beyond and from the intermediate A group at ages 65 and beyond.

**e.2.2 Other plasma biomarkers**

In parallel analyses of the whole plasma sample (n=424), the model retained the same variables as the best fitting model before adding A status to the model in the sample with A status and beta estimates were very similar except a significant *APOE*-npscore*age interaction for p-tau181 was shown in this whole sample (sensitivity analysis output in column 2-6 of Supplementary Table 8).

In exploratory analysis with PET-based 3-level A status analysis to characterize other biomarkers slopes in the PET A+ set and the A- set partitioned into those in the healthy control (low PiB DVR) subset vs those with intermediate PiB DVR, the best-fitting model included A group main effect for Aβ42/40, p-tau181 and p-tau231, A group*age for GFAP(columns 2-6 in Supplementary Table 9).

***Supplementary Figures***

**Supplementary Figure 1.** The Flowchart of this study.

**Supplementary Figure 2.** The spaghetti plot of plasma biomarkers in healthy control (N = 226).

**Supplementary Figure 3.** The spearman correlation matrix of plasma biomarkers and health factors.

**Supplementary Figure 4.** The forest plot of coefficient of variation for each plasma biomarkers in healthy control (at least 2 visits).

**Supplementary Figure 5.** Predicted mean other plasma biomarkers Z scores for significant main or interaction across age models in healthy control.

**Supplementary Figure 6.** Predicted mean other plasma biomarkers Z scores for significant main or interaction across age models in sample with known A status.

**Supplementary Figure 7.** Simple slopes and effect size for plasma p-Tau217 (pg/mls) using values representing three reference groups across age for each cognitive outcome.

***Supplementary Tables***

**Supplementary Table 1.** The means (SDs) of each biomarker used for Z scores.

**Supplementary Table 2.** Plasma Aβ42/40 z-score mixed effects model sets output in healthy control group (Aim 1).

**Supplementary Table 3.** Plasma p-tau181 z-score mixed effects model sets output in healthy control group (Aim 1). **Supplementary Table 4.** Plasma p-tau231 z-score mixed effects model sets output in healthy control group (Aim 1). **Supplementary Table 5.** Plasma GFAP z-score mixed effects model sets output in healthy control group (Aim 1). **Supplementary Table 6.** Plasma Nfl z-score mixed effects model sets output in healthy control group (Aim 1).

**Supplementary Table 7:** Sensitivity analyses of p-tau217 plasma biomarkers mixed effects output in CU participants at plasma baseline (Aim 2).

**Supplementary Table 8.** Plasma biomarkers mixed effects output in all available plasma biomarkers without A status (Aim 2).

**Supplementary Table 9.** Plasma biomarkers mixed effects output in sample with PiB PET 3 level A status in exploratory analysis (Aim 2).

**Supplementary Table 10.** Plasma Aβ42/40 z-score mixed effects model sets output in people who have A status (Aim 2).

**Supplementary Table 11.** Plasma p-tau181 z-score mixed effects model sets output in people who have A status (Aim 2).

**Supplementary Table 12.** Plasma p-tau231 z-score mixed effects model sets output in people who have A status (Aim 2).

**Supplementary Table 13.** Plasma GFAP z-score mixed effects model sets output in people who have A status (Aim 2).

**Supplementary Table 14.** Plasma Nfl z-score mixed effects model sets output in people who have A status (Aim 2).

**Supplementary Table 15.** Interaction simple slopes summary for each cognitive outcome

**Supplementary Table 16.** Cognition mixed effects output in sensitivity analysis retaining any significant plasma biomarkers in each model.

**Supplementary Table 17.** EF mixed effects output.

**Supplementary Table 18.** Immediate Memory mixed effects output.

**Supplementary Table 19.** Delayed Memory mixed effects output.

**Supplementary Table 20.** CDR Sum of Box mixed effects output.

***Supplementary Figures***

**Supplementary Figure 1.** The Flowchart of this study.

**
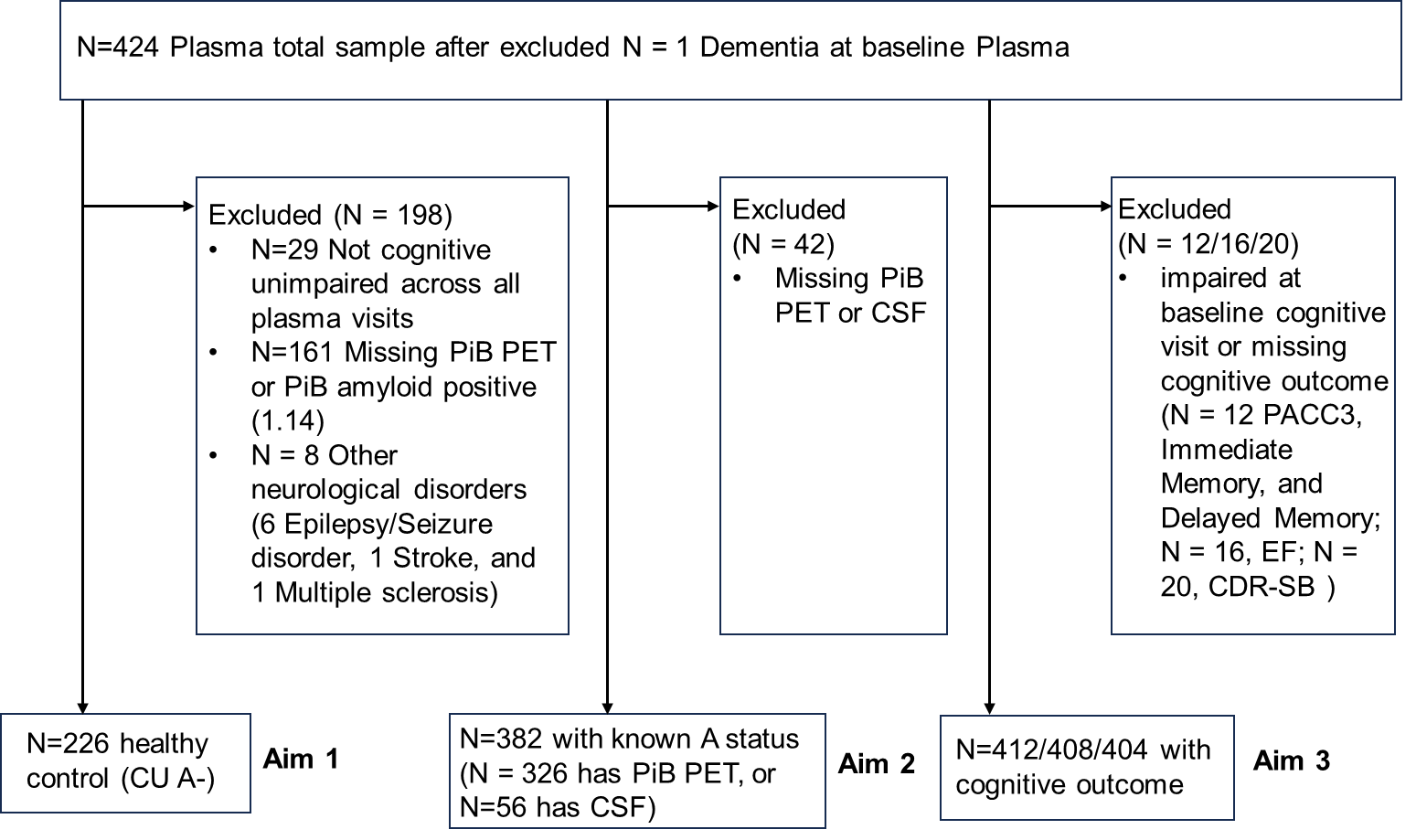
**

**Supplementary Figure 1. The Flowchart of this study.** Aim 1 focused on the longitudinal plasma p-tau217 time course of amyloid negative and cognitively normal subset of 226 participants of the original 424 who met the following criteria: were not CU at all plasma assessments (n=29), did not have an amyloid PET scan to rule out presence of amyloid (n=91), were amyloid positive on a PET scan (n=70), or self-reported one or more neurological disorders (n = 8; 6 Epilepsy/Seizure disorder, 1 Stroke, and 1 Multiple sclerosis). All participants with assayed plasma biomarkers who has PiB PET or CSF were included in Aim 2 (n=382). To be included in Aim 3, participants were required to be cognitively unimpaired (CU) at their baseline cognitive assessment and have at least one cognitive assessment (n=404 to 412, depending on the outcome).

**Supplementary Figure 2.** The spaghetti plot of plasma biomarkers in healthy control (N = 226).

**
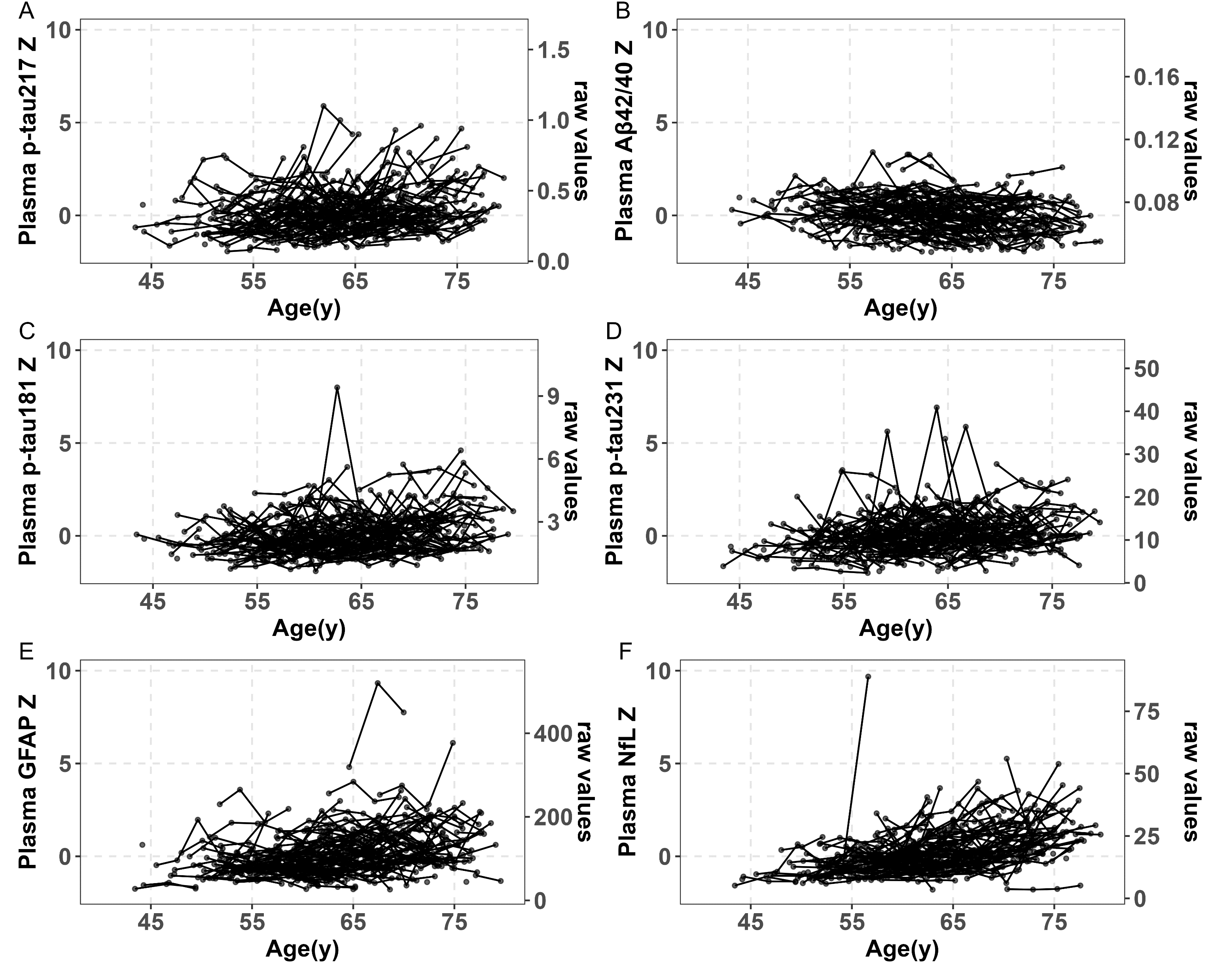
**

**Supplementary Figure 3.** The spearman correlation matrix of plasma biomarkers and health factors.

**
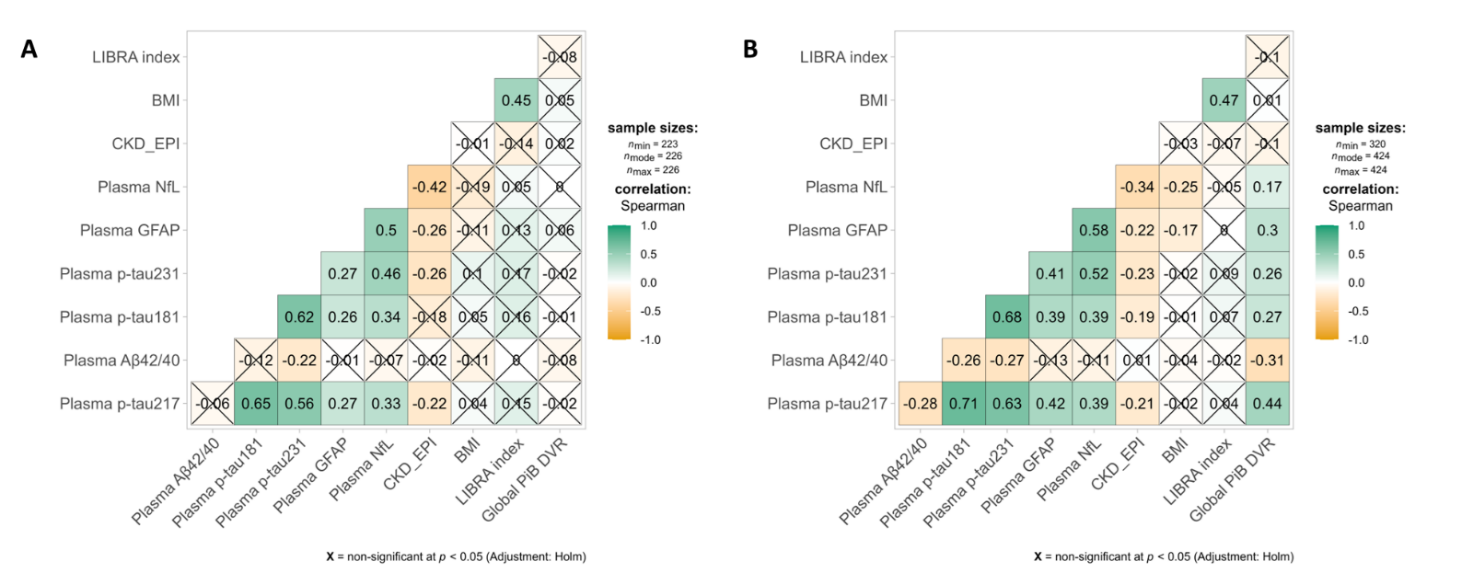
Supplementary Figure 4.** The forest plot of coefficient of variation for each plasma biomarkers in healthy control (at least 2 visits).

**
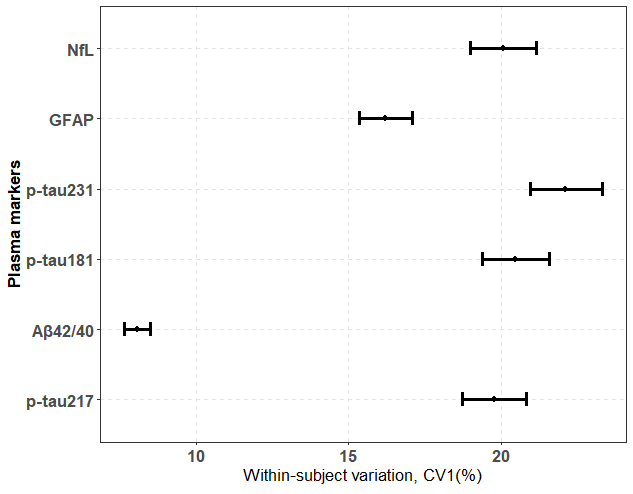
**

**Supplementary Figure 4. The forest plot of coefficient of variation for each plasma biomarkers in healthy control (at least 2 visits).** Following the paper Brum et al. “Biological variation estimates of Alzheimer's disease plasma biomarkers in healthy individuals” and we used the same method used in that paper to calculate CV; CV-ANOVA, the “Røraas method,” a validated and recommended ANOVA method for estimating CV_I_. The results shows the ab4240 has the lowest CV_I_ (8%), followed by GFAP, and ptau217, ptau181, NFL are similar, ptau231 has the highest CV_I_ (22%). But all these values are higher than the values in Brum’s paper (Aβ42/Aβ40 had the lowest CV_I_ (≈ 3%) and p-tau181 the highest (≈ 16%), while others ranged from 6% to 10%). The possible reason is the people are younger and very short follow up (10 weeks).

**Supplementary Figure 5.** Predicted mean other plasma biomarkers Z scores for significant main or interaction across age models in healthy control.

**
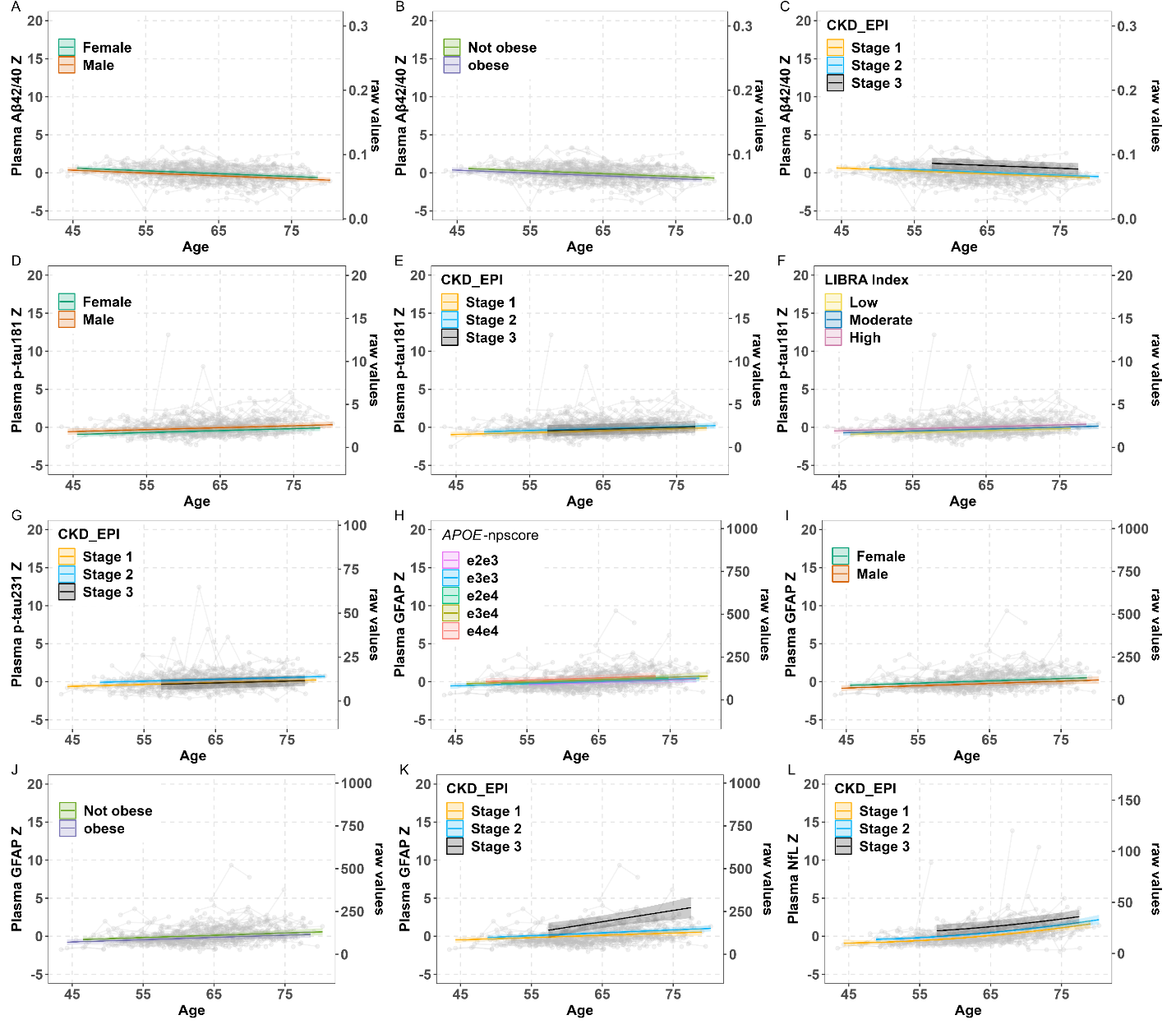
**

**Supplementary Figure 5. Predicted mean other plasma biomarkers Z scores for significant main or interaction across age models in healthy control.** The predicted mean plasma biomarkers Z scores for plasma is on the y-axis, and age in years is represented on the x-axis. Estimates come from regression models for Aβ42/40 (A, B and C), p-tau181 (D, E and F), p-tau231(G), GFAP (H, I, J, K), and Nfl (L) in healthy control subset. Bands represent 95% CIs. Estimates are truncated to be within the age range of participants for a particular predictor group. APOE, apolipoprotein E; CI, confidence interval. Note that the APOE score was included as a continuous measure in the regression analyses. For ease of interpretation, estimates in figures are provided for APOE scores that correspond to APOE genotypes as opposed to whole units or percentiles of the APOE score.

**Supplementary Figure 6.** Predicted mean other plasma biomarkers Z scores for significant main or interaction across age models in sample with known A status.


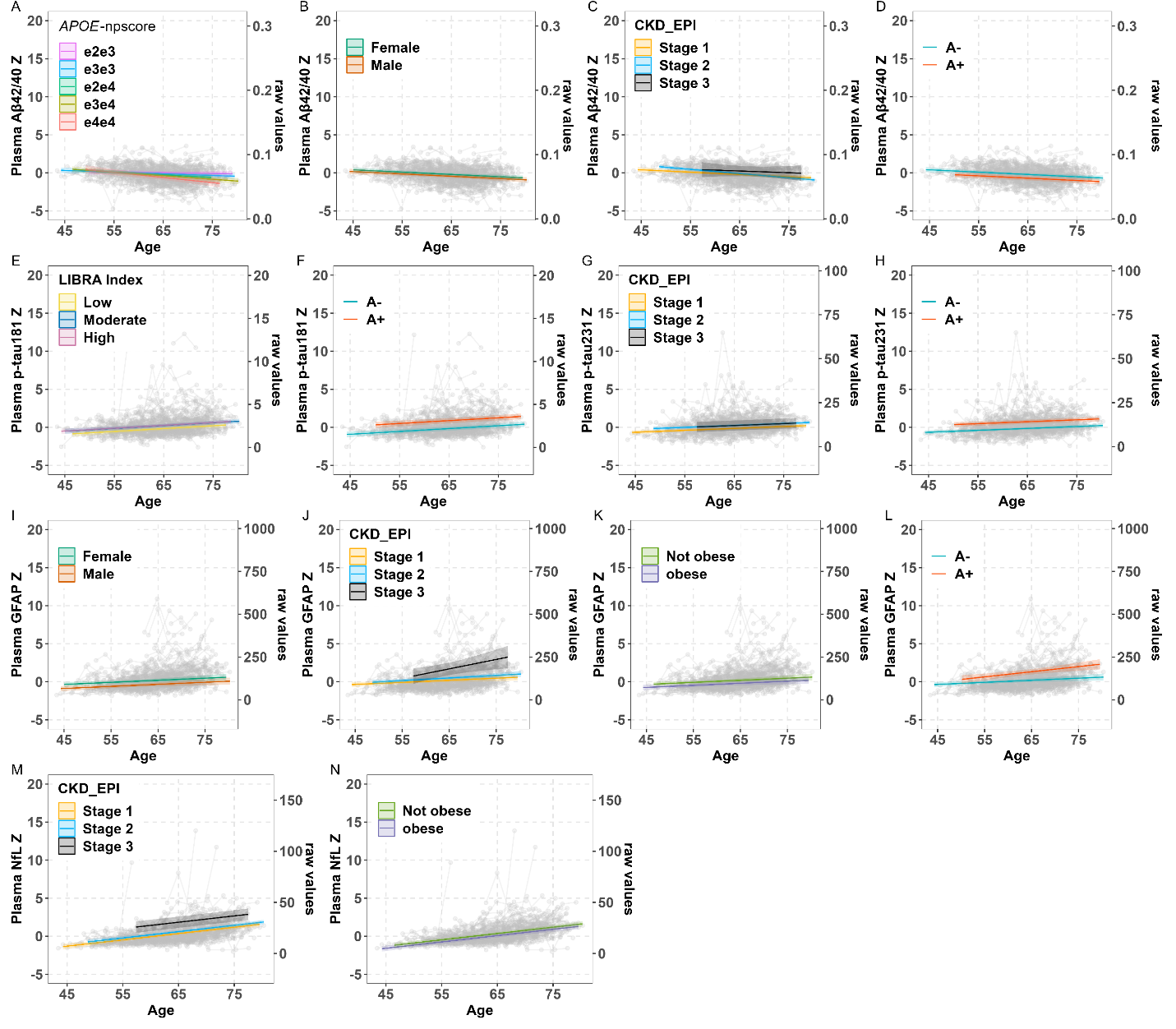


**Supplementary Figure 6. Predicted mean other plasma biomarkers Z scores for significant main or interaction across age models in sample with known A status.** The predicted mean plasma biomarkers Z scores for plasma is on the y-axis, and age in years is represented on the x-axis. Estimates come from regression models for Aβ42/40 (A, B, C and D), p-tau181 (E and F), p-tau231(G and H), GFAP (I, J, K and L), and Nfl (M and N) in sample with known A status. Bands represent 95% CIs. Estimates are truncated to be within the age range of participants for a particular predictor group. APOE, apolipoprotein E; CI, confidence interval. Note that the APOE score was included as a continuous measure in the regression analyses. For ease of interpretation, estimates in figures are provided for APOE scores that correspond to APOE genotypes as opposed to whole units or percentiles of the APOE score.

**Supplementary Figure 7.** Simple slopes and effect size for plasma p-Tau217 (pg/mls) using values representing three reference groups across age for each cognitive outcome.**
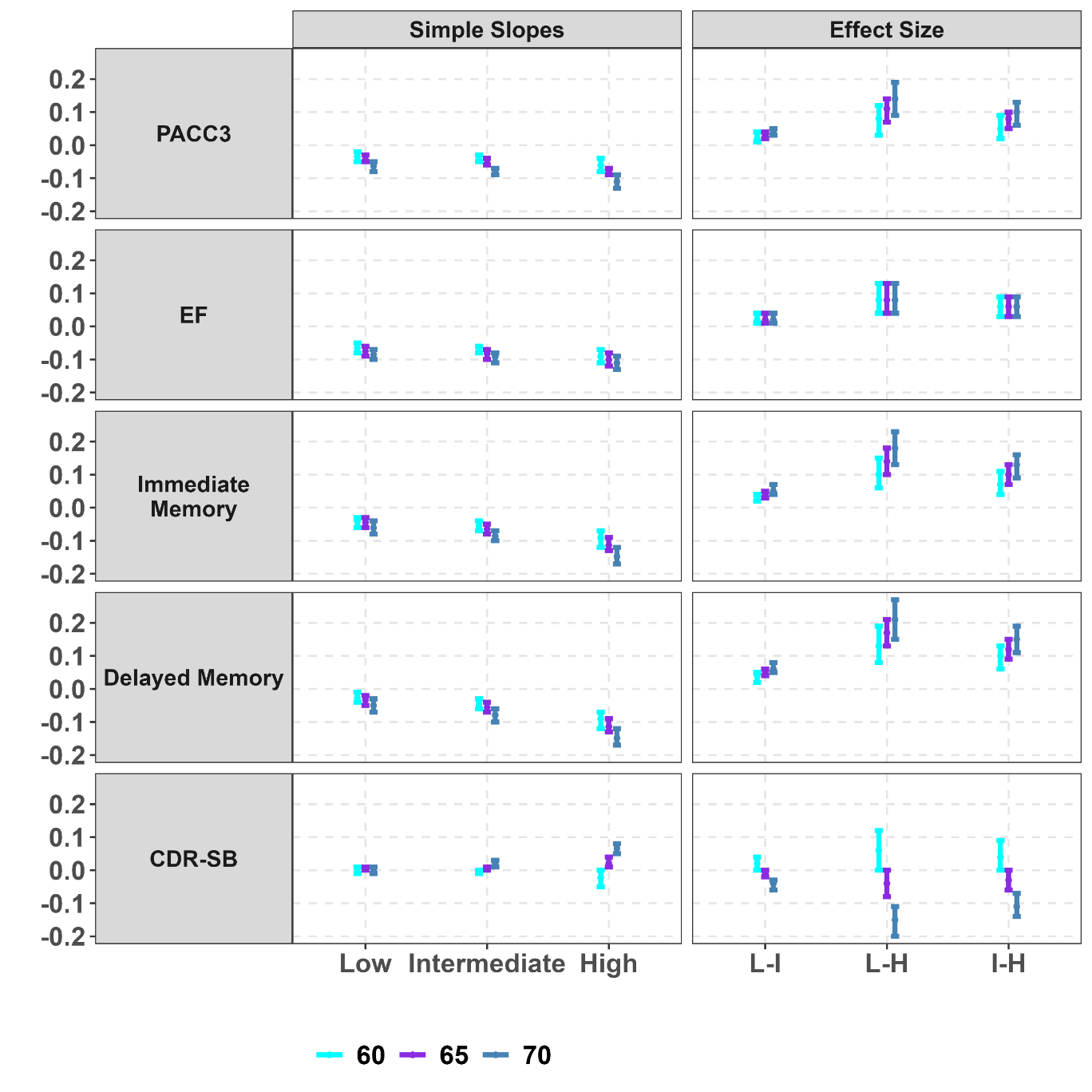
**

**Supplementary Figure 7. Simple slopes and effect size for plasma p-Tau217 (pg/mls) using values representing three reference groups across age for each cognitive outcome.** Left panels depict simple slopes and 95% CI’s estimated at ages 60, 65, and 70 for values representing low, intermediate, and high plasma pTau217 for each outcome. Right panel depict the pairwise comparisons of simple slopes. The asterisk shows the slope at first age is different from 0 at that p-tau217 group. Note: The model 1 for EF did not include a quadratic age term, so pairwise differences are the same across all ages.

***eTables***

| **SupplementaryTable 1. The means (SDs) of each biomarker used for Z scores** |
| --- |

|  | **Mean (SDs), pg/mL** |
| --- | --- |
| **ALZpath p-tau217** | 0.325 (0.13) |
| **Aβ42/40** | 0.0716 (0.012) |
| **p-tau181** | 2.333 (0.89) |
| **p-tau231** | 10.959 (4.33) |
| **GFAP** | 105.494 (44.43) |
| **NfL** | 16.86 (7.44) |

**Supplementary Table 2: Plasma Aβ42/40 z-score mixed effects model sets output in healthy control group (Aim 1)**

|  | **1 (predictor=sex)** | | | **2 (predictor=npscore)** | | | **3 (predictor=BMI)** | | | **4 (predictor=CKD EPI)** | | | **5 (predictor=LIBRA)** | | | **6 (combined)** | | |
| --- | --- | --- | --- | --- | --- | --- | --- | --- | --- | --- | --- | --- | --- | --- | --- | --- | --- | --- |
| *Predictors* | *Estimates* | *CI* | *p* | *Estimates* | *CI* | *p* | *Estimates* | *CI* | *p* | *Estimates* | *CI* | *p* | *Estimates* | *CI* | *p* | *Estimates* | *CI* | *p* |
| (Intercept) | 0.10 | -0.06 – 0.26 | 0.199 | 0.07 | -0.08 – 0.22 | 0.372 | 0.09 | -0.07 – 0.26 | 0.255 | -0.11 | -0.30 – 0.08 | 0.267 | 0.07 | -0.14 – 0.28 | 0.491 | 0.07 | -0.16 – 0.30 | 0.546 |
| c60age | -0.03 | -0.05 – -0.02 | **<0.001** | -0.04 | -0.05 – -0.02 | **<0.001** | -0.04 | -0.05 – -0.02 | **<0.001** | -0.04 | -0.05 – -0.02 | **<0.001** | -0.04 | -0.05 – -0.02 | **<0.001** | -0.04 | -0.05 – -0.02 | **<0.001** |
| Male | -0.28 | -0.56 – -0.01 | **0.042** |  |  |  |  |  |  |  |  |  |  |  |  | -0.28 | -0.55 – -0.01 | **0.044** |
| npscore |  |  |  | -0.11 | -0.24 – 0.03 | 0.120 |  |  |  |  |  |  |  |  |  |  |  |  |
| obese |  |  |  |  |  |  | -0.23 | -0.50 – 0.04 | 0.090 |  |  |  |  |  |  | -0.28 | -0.54 – -0.01 | **0.041** |
| CKD EPI [Stage 2] |  |  |  |  |  |  |  |  |  | 0.19 | -0.07 – 0.45 | 0.147 |  |  |  | 0.19 | -0.07 – 0.45 | 0.154 |
| CKD EPI [Stage 3] |  |  |  |  |  |  |  |  |  | 1.13 | 0.34 – 1.91 | **0.005** |  |  |  | 1.08 | 0.30 – 1.86 | **0.007** |
| LIBRA  [Moderate] |  |  |  |  |  |  |  |  |  |  |  |  | -0.15 | -0.48 – 0.19 | 0.394 |  |  |  |
| LIBRA [High] |  |  |  |  |  |  |  |  |  |  |  |  | 0.01 | -0.32 – 0.34 | 0.965 |  |  |  |
| **Random Effects** | | | | | | | | | | | | | | | | | | |
| σ^2^ | 0.27 | | | 0.27 | | | 0.27 | | | 0.27 | | | 0.27 | | | 0.27 | | |
| τ_00_ | 0.83 _Reggieid_ | | | 0.84 _Reggieid_ | | | 0.83 _Reggieid_ | | | 0.81 _Reggieid_ | | | 0.84 _Reggieid_ | | | 0.79 _Reggieid_ | | |
| ICC | 0.75 | | | 0.75 | | | 0.75 | | | 0.75 | | | 0.76 | | | 0.74 | | |
| N | 222 _Reggieid_ | | | 222 _Reggieid_ | | | 222 _Reggieid_ | | | 222 _Reggieid_ | | | 222 _Reggieid_ | | | 222 _Reggieid_ | | |
| Observations | 665 | | | 665 | | | 665 | | | 665 | | | 665 | | | 665 | | |
| Marginal R^2^ / Conditional R^2^ | 0.068 / 0.769 | | | 0.055 / 0.768 | | | 0.062 / 0.769 | | | 0.075 / 0.768 | | | 0.055 / 0.770 | | | 0.102 / 0.770 | | |
| AICc | 1534.9 | | | 1536.7 | | | 1536.2 | | | 1532.4 | | | 1539.9 | | | 1529.0 | | |
| $\Delta$AICc | -2.14 | | | -0.40 | | | -0.87 | | | -4.66 | | | 2.82 | | | -8.10 | | |

***The base model is age fixed effects up to cubic polynomial (retaining highest order significant term and lower order terms; centered at age 60) and random effects. Models 1-5 started with predictor*age included and then removed if NS. Model 6 brings in significant main effects and interactions from Models 1-5, removes NS interactions sequentially (least significant out first) until only significant interactions (and their supporting main effects) or significant main effect (if no corresponding interactions are significant) remain.** $\boldsymbol{\Delta}$**AICc is calculated relative to the base model; negative numbers indicate better fit in the expanded model.**

**Supplementary Table 3: Plasma p-tau181 z-score mixed effects model sets output in healthy control group (Aim 1)**

|  | **1 (predictor=sex)** | | | **2 (predictor=npscore)** | | | **3 (predictor=BMI)** | | | **4 (predictor=CKD EPI)** | | | **5 (predictor=LIBRA)** | | | **6 (combined)** | | |
| --- | --- | --- | --- | --- | --- | --- | --- | --- | --- | --- | --- | --- | --- | --- | --- | --- | --- | --- |
| *Predictors* | *Estimates* | *CI* | *p* | *Estimates* | *CI* | *p* | *Estimates* | *CI* | *p* | *Estimates* | *CI* | *p* | *Estimates* | *CI* | *p* | *Estimates* | *CI* | *p* |
| (Intercept) | -0.14 | -0.30 – 0.01 | 0.066 | -0.04 | -0.19 – 0.11 | 0.571 | -0.13 | -0.29 – 0.03 | 0.105 | -0.19 | -0.38 – -0.01 | **0.041** | -0.35 | -0.60 – -0.11 | **0.005** | -0.56 | -0.83 – -0.29 | **<0.001** |
| c60age | 0.03 | 0.02 – 0.05 | **<0.001** | 0.03 | 0.02 – 0.05 | **<0.001** | 0.03 | 0.02 – 0.05 | **<0.001** | 0.03 | 0.01 – 0.04 | **0.001** | 0.03 | 0.02 – 0.05 | **<0.001** | 0.03 | 0.01 – 0.04 | **0.001** |
| Male | 0.34 | 0.08 – 0.60 | **0.010** |  |  |  |  |  |  |  |  |  |  |  |  | 0.39 | 0.13 – 0.64 | **0.003** |
| npscore |  |  |  | 0.00 | -0.12 – 0.13 | 0.943 |  |  |  |  |  |  |  |  |  |  |  |  |
| obese |  |  |  |  |  |  | 0.27 | 0.01 – 0.53 | **0.040** |  |  |  |  |  |  |  |  |  |
| CKD EPI [Stage 2] |  |  |  |  |  |  |  |  |  | 0.30 | 0.05 – 0.55 | **0.021** |  |  |  | 0.27 | 0.02 – 0.52 | **0.033** |
| CKD EPI [Stage 3] |  |  |  |  |  |  |  |  |  | 0.22 | -0.53 – 0.97 | 0.568 |  |  |  | 0.18 | -0.55 – 0.92 | 0.627 |
| LIBRA  [Moderate] |  |  |  |  |  |  |  |  |  |  |  |  | 0.28 | -0.03 – 0.60 | 0.077 | 0.20 | -0.11 – 0.52 | 0.203 |
| LIBRA [High] |  |  |  |  |  |  |  |  |  |  |  |  | 0.54 | 0.23 – 0.84 | **0.001** | 0.49 | 0.18 – 0.80 | **0.002** |
| **Random Effects** | | | | | | | | | | | | | | | | | | |
| σ^2^ | 0.72 | | | 0.72 | | | 0.72 | | | 0.72 | | | 0.72 | | | 0.72 | | |
| τ_00_ | 0.58 _Reggieid_ | | | 0.60 _Reggieid_ | | | 0.58 _Reggieid_ | | | 0.59 _Reggieid_ | | | 0.56 _Reggieid_ | | | 0.53 _Reggieid_ | | |
| ICC | 0.44 | | | 0.45 | | | 0.45 | | | 0.45 | | | 0.44 | | | 0.43 | | |
| N | 222 _Reggieid_ | | | 222 _Reggieid_ | | | 222 _Reggieid_ | | | 222 _Reggieid_ | | | 222 _Reggieid_ | | | 222 _Reggieid_ | | |
| Observations | 661 | | | 661 | | | 661 | | | 661 | | | 661 | | | 661 | | |
| Marginal R^2^ / Conditional R^2^ | 0.055 / 0.475 | | | 0.036 / 0.473 | | | 0.048 / 0.475 | | | 0.053 / 0.478 | | | 0.069 / 0.477 | | | 0.103 / 0.485 | | |
| AICc | 1931.7 | | | 1938.4 | | | 1934.1 | | | 1935.0 | | | 1928.6 | | | 1922.3 | | |
| $\Delta$AICc | -4.63 | | | 2.03 | | | -2.23 | | | -1.36 | | | -7.73 | | | -14.05 | | |

***The base model is age fixed effects up to cubic polynomial (retaining highest order significant term and lower order terms; centered at age 60) and random effects. Models 1-5 started with predictor*age included and then removed if NS. Model 6 bring in significant main effects and interactions from Models 1-5, remove NS interactions sequentially (least significant out first) until only significant interactions (and their supporting main effects) or significant main effect (if no corresponding interactions are significant) remain.** $\boldsymbol{\Delta}$**AICc is calculated relative to the base model; negative numbers indicate better fit in the expanded model.**

**Supplementary Table 4: Plasma p-tau231 z-score mixed effects model sets output in healthy control group (Aim 1)**

|  | **1 (predictor=sex)** | | | **2 (predictor=npscore)** | | | **3 (predictor=BMI)** | | | **4 (predictor=CKD EPI)** | | | **5 (predictor=LIBRA)** | | | **6 (combined)** | | |
| --- | --- | --- | --- | --- | --- | --- | --- | --- | --- | --- | --- | --- | --- | --- | --- | --- | --- | --- |
| *Predictors* | *Estimates* | *CI* | *p* | *Estimates* | *CI* | *p* | *Estimates* | *CI* | *p* | *Estimates* | *CI* | *p* | *Estimates* | *CI* | *p* | *Estimates* | *CI* | *p* |
| (Intercept) | -0.03 | -0.19 – 0.12 | 0.667 | -0.01 | -0.16 – 0.14 | 0.937 | -0.10 | -0.26 – 0.06 | 0.224 | -0.23 | -0.41 – -0.05 | **0.014** | -0.22 | -0.47 – 0.03 | 0.090 | -0.23 | -0.41 – -0.05 | **0.014** |
| c60age | 0.03 | 0.01 – 0.05 | **<0.001** | 0.03 | 0.01 – 0.05 | **<0.001** | 0.03 | 0.01 – 0.05 | **<0.001** | 0.02 | 0.01 – 0.04 | **0.002** | 0.03 | 0.01 – 0.04 | **<0.001** | 0.02 | 0.01 – 0.04 | **0.002** |
| Male | 0.06 | -0.20 – 0.32 | 0.656 |  |  |  |  |  |  |  |  |  |  |  |  |  |  |  |
| npscore |  |  |  | -0.02 | -0.15 – 0.11 | 0.771 |  |  |  |  |  |  |  |  |  |  |  |  |
| obese |  |  |  |  |  |  | 0.25 | -0.01 – 0.50 | 0.060 |  |  |  |  |  |  |  |  |  |
| CKD EPI [Stage 2] |  |  |  |  |  |  |  |  |  | 0.43 | 0.18 – 0.68 | **0.001** |  |  |  | 0.43 | 0.18 – 0.68 | **0.001** |
| CKD EPI [Stage 3] |  |  |  |  |  |  |  |  |  | -0.05 | -0.79 – 0.69 | 0.893 |  |  |  | -0.05 | -0.79 – 0.69 | 0.893 |
| LIBRA  [Moderate] |  |  |  |  |  |  |  |  |  |  |  |  | 0.18 | -0.14 – 0.50 | 0.278 |  |  |  |
| LIBRA [High] |  |  |  |  |  |  |  |  |  |  |  |  | 0.35 | 0.04 – 0.66 | **0.028** |  |  |  |
| **Random Effects** | | | | | | | | | | | | | | | | | | |
| σ^2^ | 0.73 | | | 0.73 | | | 0.73 | | | 0.73 | | | 0.73 | | | 0.73 | | |
| τ_00_ | 0.60 _Reggieid_ | | | 0.60 _Reggieid_ | | | 0.59 _Reggieid_ | | | 0.56 _Reggieid_ | | | 0.59 _Reggieid_ | | | 0.56 _Reggieid_ | | |
| ICC | 0.45 | | | 0.45 | | | 0.45 | | | 0.44 | | | 0.44 | | | 0.44 | | |
| N | 222 _Reggieid_ | | | 222 _Reggieid_ | | | 222 _Reggieid_ | | | 222 _Reggieid_ | | | 222 _Reggieid_ | | | 222 _Reggieid_ | | |
| Observations | 665 | | | 665 | | | 665 | | | 665 | | | 665 | | | 665 | | |
| Marginal R^2^ / Conditional R^2^ | 0.032 / 0.468 | | | 0.031 / 0.468 | | | 0.041 / 0.469 | | | 0.066 / 0.473 | | | 0.045 / 0.470 | | | 0.066 / 0.473 | | |
| AICc | 1957.5 | | | 1957.6 | | | 1954.1 | | | 1947.6 | | | 1954.8 | | | 1947.6 | | |
| $\Delta$AICc | 1.83 | | | 1.95 | | | -1.52 | | | -8.11 | | | -0.89 | | | -8.11 | | |

***The base model is age fixed effects up to cubic polynomial (retaining highest order significant term and lower order terms; centered at age 60) and random effects. Models 1-5 started with predictor*age included and then removed if NS. Model 6 bring in significant main effects and interactions from Models 1-5, remove NS interactions sequentially (least significant out first) until only significant interactions (and their supporting main effects) or significant main effect (if no corresponding interactions are significant) remain.** $\boldsymbol{\Delta}$**AICc is calculated relative to the base model; negative numbers indicate better fit in the expanded model.**

**Supplementary Table 5: Plasma GFAP z-score mixed effects model sets output in healthy control group (Aim 1)**

|  | **1 (predictor=sex)** | | | **2 (predictor=npscore)** | | | **3 (predictor=BMI)** | | | **4 (predictor=CKD EPI)** | | | **5 (predictor=LIBRA)** | | | **6 (combined)** | | |
| --- | --- | --- | --- | --- | --- | --- | --- | --- | --- | --- | --- | --- | --- | --- | --- | --- | --- | --- |
| *Predictors* | *Estimates* | *CI* | *p* | *Estimates* | *CI* | *p* | *Estimates* | *CI* | *p* | *Estimates* | *CI* | *p* | *Estimates* | *CI* | *p* | *Estimates* | *CI* | *p* |
| (Intercept) | 0.08 | -0.06 – 0.23 | 0.261 | -0.09 | -0.23 – 0.06 | 0.239 | 0.04 | -0.12 – 0.19 | 0.655 | -0.21 | -0.39 – -0.02 | **0.027** | -0.06 | -0.31 – 0.19 | 0.637 | -0.09 | -0.32 – 0.14 | 0.441 |
| c60age | 0.04 | 0.02 – 0.05 | **<0.001** | 0.04 | 0.03 – 0.05 | **<0.001** | 0.04 | 0.02 – 0.05 | **<0.001** | 0.03 | 0.01 – 0.05 | **0.010** | 0.04 | 0.02 – 0.05 | **<0.001** | 0.03 | 0.01 – 0.05 | **0.002** |
| Male | -0.38 | -0.65 – -0.11 | **0.006** |  |  |  |  |  |  |  |  |  |  |  |  | -0.34 | -0.61 – -0.08 | **0.010** |
| npscore |  |  |  | 0.11 | -0.02 – 0.24 | 0.099 |  |  |  |  |  |  |  |  |  | 0.13 | 0.01 – 0.26 | **0.036** |
| obese |  |  |  |  |  |  | -0.19 | -0.46 – 0.08 | 0.161 |  |  |  |  |  |  | -0.28 | -0.53 – -0.02 | **0.033** |
| CKD EPI [Stage 2] |  |  |  |  |  |  |  |  |  | 0.27 | 0.01 – 0.52 | **0.039** |  |  |  | 0.29 | 0.04 – 0.54 | **0.023** |
| CKD EPI [Stage 3] |  |  |  |  |  |  |  |  |  | 1.19 | 0.28 – 2.11 | **0.011** |  |  |  | 1.20 | 0.29 – 2.10 | **0.010** |
| c60age × CKD EPI  [Stage 2] |  |  |  |  |  |  |  |  |  | 0.01 | -0.02 – 0.04 | 0.540 |  |  |  | 0.01 | -0.02 – 0.03 | 0.551 |
| c60age × CKD EPI  [Stage 3] |  |  |  |  |  |  |  |  |  | 0.12 | 0.03 – 0.21 | **0.008** |  |  |  | 0.12 | 0.03 – 0.21 | **0.008** |
| LIBRA  [Moderate] |  |  |  |  |  |  |  |  |  |  |  |  | -0.02 | -0.35 – 0.30 | 0.882 |  |  |  |
| LIBRA [High] |  |  |  |  |  |  |  |  |  |  |  |  | 0.10 | -0.22 – 0.43 | 0.528 |  |  |  |
| **Random Effects** | | | | | | | | | | | | | | | | | | |
| σ^2^ | 0.26 | | | 0.26 | | | 0.26 | | | 0.26 | | | 0.26 | | | 0.27 | | |
| τ_00_ | 0.70 _Reggieid_ | | | 0.73 _Reggieid_ | | | 0.74 _Reggieid_ | | | 0.70 _Reggieid_ | | | 0.74 _Reggieid_ | | | 0.67 _Reggieid_ | | |
| τ_11_ | 0.00 _Reggieid.c60age_ | | | 0.00 _Reggieid.c60age_ | | | 0.00 _Reggieid.c60age_ | | | 0.00 _Reggieid.c60age_ | | | 0.00 _Reggieid.c60age_ | | | 0.00 _Reggieid.c60age_ | | |
| ρ_01_ | 0.74 _Reggieid_ | | | 0.78 _Reggieid_ | | | 0.77 _Reggieid_ | | | 0.73 _Reggieid_ | | | 0.72 _Reggieid_ | | | 1.00 _Reggieid_ | | |
| ICC | 0.79 | | | 0.79 | | | 0.79 | | | 0.78 | | | 0.79 | | | 0.76 | | |
| N | 222 _Reggieid_ | | | 222 _Reggieid_ | | | 222 _Reggieid_ | | | 222 _Reggieid_ | | | 222 _Reggieid_ | | | 222 _Reggieid_ | | |
| Observations | 664 | | | 664 | | | 664 | | | 664 | | | 664 | | | 664 | | |
| Marginal R^2^ / Conditional R^2^ | 0.070 / 0.805 | | | 0.055 / 0.802 | | | 0.056 / 0.803 | | | 0.157 / 0.811 | | | 0.053 / 0.805 | | | 0.187 / 0.809 | | |
| AICc | 1547.5 | | | 1552.3 | | | 1553.0 | | | 1538.3 | | | 1556.2 | | | 1530.2 | | |
| $\Delta$AICc | -5.42 | | | -0.64 | | | 0.097 | | | -14.67 | | | 3.25 | | | -22.76 | | |

***The base model is age fixed effects up to cubic polynomial (retaining highest order significant term and lower order terms; centered at age 60) and random effects. Models 1-5 started with predictor*age included and then removed if NS. Model 6 bring in significant main effects and interactions from Models 1-5, remove NS interactions sequentially (least significant out first) until only significant interactions (and their supporting main effects) or significant main effect (if no corresponding interactions are significant) remain.** $\boldsymbol{\Delta}$**AICc is calculated relative to the base model; negative numbers indicate better fit in the expanded model.**

**Supplementary Table 6: Plasma Nfl z-score mixed effects model sets output in healthy control group (Aim 1)**

|  | **1 (predictor=sex)** | | | **2 (predictor=npscore)** | | | **3 (predictor=BMI)** | | | **4 (predictor=CKD EPI)** | | | **5 (predictor=LIBRA)** | | | **6 (combined)** | | |
| --- | --- | --- | --- | --- | --- | --- | --- | --- | --- | --- | --- | --- | --- | --- | --- | --- | --- | --- |
| *Predictors* | *Estimates* | *CI* | *p* | *Estimates* | *CI* | *p* | *Estimates* | *CI* | *p* | *Estimates* | *CI* | *p* | *Estimates* | *CI* | *p* | *Estimates* | *CI* | *p* |
| (Intercept) | -0.03 | -0.18 – 0.13 | 0.744 | -0.08 | -0.23 – 0.07 | 0.281 | -0.02 | -0.18 – 0.14 | 0.798 | -0.29 | -0.47 – -0.11 | **0.001** | -0.15 | -0.38 – 0.09 | 0.228 | -0.29 | -0.47 – -0.11 | **0.001** |
| c60age | 0.07 | 0.06 – 0.09 | **<0.001** | 0.07 | 0.06 – 0.09 | **<0.001** | 0.07 | 0.06 – 0.09 | **<0.001** | 0.07 | 0.05 – 0.08 | **<0.001** | 0.07 | 0.06 – 0.09 | **<0.001** | 0.07 | 0.05 – 0.08 | **<0.001** |
| c60age^2 | 0.00 | 0.00 – 0.00 | **0.046** | 0.00 | 0.00 – 0.00 | **0.049** | 0.00 | 0.00 – 0.00 | **0.045** | 0.00 | 0.00 – 0.00 | **0.030** | 0.00 | -0.00 – 0.00 | 0.050 | 0.00 | 0.00 – 0.00 | **0.030** |
| Male | -0.16 | -0.41 – 0.09 | 0.216 |  |  |  |  |  |  |  |  |  |  |  |  |  |  |  |
| npscore |  |  |  | 0.02 | -0.11 – 0.14 | 0.794 |  |  |  |  |  |  |  |  |  |  |  |  |
| obese |  |  |  |  |  |  | -0.16 | -0.41 – 0.08 | 0.191 |  |  |  |  |  |  |  |  |  |
| CKD EPI [Stage 2] |  |  |  |  |  |  |  |  |  | 0.39 | 0.16 – 0.63 | **0.001** |  |  |  | 0.39 | 0.16 – 0.63 | **0.001** |
| CKD EPI [Stage 3] |  |  |  |  |  |  |  |  |  | 1.17 | 0.34 – 1.99 | **0.005** |  |  |  | 1.17 | 0.34 – 1.99 | **0.005** |
| LIBRA  [Moderate] |  |  |  |  |  |  |  |  |  |  |  |  | 0.07 | -0.23 – 0.37 | 0.653 |  |  |  |
| LIBRA [High] |  |  |  |  |  |  |  |  |  |  |  |  | 0.13 | -0.17 – 0.42 | 0.404 |  |  |  |
| **Random Effects** | | | | | | | | | | | | | | | | | | |
| σ^2^ | 0.78 | | | 0.78 | | | 0.78 | | | 0.79 | | | 0.78 | | | 0.79 | | |
| τ_00_ | 0.40 _Reggieid_ | | | 0.39 _Reggieid_ | | | 0.40 _Reggieid_ | | | 0.36 _Reggieid_ | | | 0.39 _Reggieid_ | | | 0.36 _Reggieid_ | | |
| τ_11_ | 0.00 _Reggieid.c60age_ | | | 0.00 _Reggieid.c60age_ | | | 0.00 _Reggieid.c60age_ | | | 0.00 _Reggieid.c60age_ | | | 0.00 _Reggieid.c60age_ | | | 0.00 _Reggieid.c60age_ | | |
| ρ_01_ | 0.91 _Reggieid_ | | | 0.86 _Reggieid_ | | | 0.89 _Reggieid_ | | | 0.99 _Reggieid_ | | | 0.86 _Reggieid_ | | | 0.99 _Reggieid_ | | |
| ICC | 0.50 | | | 0.50 | | | 0.50 | | | 0.48 | | | 0.50 | | | 0.48 | | |
| N | 222 _Reggieid_ | | | 222 _Reggieid_ | | | 222 _Reggieid_ | | | 222 _Reggieid_ | | | 222 _Reggieid_ | | | 222 _Reggieid_ | | |
| Observations | 665 | | | 665 | | | 665 | | | 665 | | | 665 | | | 665 | | |
| Marginal R^2^ / Conditional R^2^ | 0.186 / 0.594 | | | 0.185 / 0.595 | | | 0.189 / 0.591 | | | 0.223 / 0.593 | | | 0.186 / 0.596 | | | 0.223 / 0.593 | | |
| AICc | 2011.7 | | | 2013.3 | | | 2011.6 | | | 1999.3 | | | 2014.7 | | | 1999.3 | | |
| $\Delta$AICc | 0.46 | | | 1.97 | | | 0.31 | | | -11.94 | | | 3.38 | | | -11.94 | | |

***The base model is age fixed effects up to cubic polynomial (retaining highest order significant term and lower order terms; centered at age 60) and random effects. Models 1-5 started with predictor*age included and then removed if NS. Model 6 bring in significant main effects and interactions from Models 1-5, remove NS interactions sequentially (least significant out first) until only significant interactions (and their supporting main effects) or significant main effect (if no corresponding interactions are significant) remain.** $\boldsymbol{\Delta}$**AICc is calculated relative to the base model; negative numbers indicate better fit in the expanded model.**

**Supplementary Table 7: Sensitivity analyses of p-tau217 plasma biomarkers mixed effects output in CU participants at plasma baseline (Aim 2).**

|  | 1 (predictor=sex) | | | 2 (predictor=npscore) | | | 3 (predictor=BMI) | | | 4 (predictor=CKD EPI) | | | 5 (predictor=LIBRA) | | | 6 (combined) | | | 7 (A status) | | |
| --- | --- | --- | --- | --- | --- | --- | --- | --- | --- | --- | --- | --- | --- | --- | --- | --- | --- | --- | --- | --- | --- |
| *Predictors* | *Estimates* | *CI* | *p* | *Estimates* | *CI* | *p* | *Estimates* | *CI* | *p* | *Estimates* | *CI* | *p* | *Estimates* | *CI* | *p* | *Estimates* | *CI* | *p* | *Estimates* | *CI* | *p* |
| (Intercept) | 0.17 | -0.03 – 0.38 | 0.103 | -0.03 | -0.23 – 0.17 | 0.767 | 0.24 | 0.03 – 0.45 | **0.027** | 0.09 | -0.16 – 0.35 | 0.474 | 0.15 | -0.17 – 0.48 | 0.354 | -0.03 | -0.23 – 0.17 | 0.767 | -0.19 | -0.38 – -0.00 | **0.048** |
| c60age | .09 | 0.06 – 0.11 | **<0.001** | 0.07 | 0.04 – 0.10 | **<0.001** | 0.09 | 0.06 – 0.11 | **<0.001** | 0.08 | 0.06 – 0.11 | **<0.001** | 0.09 | 0.06 – 0.11 | **<0.001** | 0.07 | 0.04 – 0.10 | **<0.001** | 0.04 | 0.02 – 0.07 | **<0.001** |
| c60age^2 | 0.01 | 0.00 – 0.01 | **<0.001** | 0.00 | 0.00 – 0.01 | **0.001** | 0.01 | 0.00 – 0.01 | **<0.001** | 0.01 | 0.00 – 0.01 | **<0.001** | 0.01 | 0.00 – 0.01 | **<0.001** | 0.00 | 0.00 – 0.01 | **0.001** | 0.00 | 0.00 – 0.00 | **0.027** |
| Male | 0.15 | -0.20 – 0.50 | 0.402 |  |  |  |  |  |  |  |  |  |  |  |  |  |  |  |  |  |  |
| npscore |  |  |  | 0.38 | 0.22 – 0.54 | **<0.001** |  |  |  |  |  |  |  |  |  | 0.38 | 0.22 – 0.54 | **<0.001** | 0.25 | 0.10 – 0.40 | **0.001** |
| c60age × npscore |  |  |  | .04 | 0.02 – 0.06 | **0.001** |  |  |  |  |  |  |  |  |  | 0.04 | 0.02 – 0.06 | **0.001** | .04 |  |  |
| c60age^2 × npscore |  |  |  | 0.00 | 0.00 – 0.01 | **<0.001** | 0.00 |  |  |  |  |  |  |  |  | 0.00 | 0.00 – 0.01 | **<0.001** |  |  |  |
| obese |  |  |  |  |  |  | -0.06 | -0.40 – 0.29 | 0.747 |  |  |  |  |  |  |  |  |  |  |  |  |
| CKD EPI [Stage 2] |  |  |  |  |  |  |  |  |  | 0.23 | -0.10 – 0.56 | 0.175 |  |  |  |  |  |  |  |  |  |
| CKD EPI [Stage 3] |  |  |  |  |  |  |  |  |  | 0.07 | -1.27 – 1.41 | 0.922 |  |  |  |  |  |  |  |  |  |
| LIBRA  [Moderate] |  |  |  |  |  |  |  |  |  |  |  |  | 0.07 | -0.35 – 0.50 | 0.733 |  |  |  |  |  |  |
| LIBRA [High] |  |  |  |  |  |  |  |  |  |  |  |  | 0.10 | -0.31 – 0.51 | 0.643 |  |  |  |  |  |  |
| A+ |  |  |  |  |  |  |  |  |  |  |  |  |  |  |  |  |  |  | 1.45 | 0.98 – 1.93 | **<0.001** |
| c60age × A+ |  |  |  |  |  |  |  |  |  |  |  |  |  |  |  |  |  |  | 0.17 | 0.09 – 0.25 | **<0.001** |
| c60age^2  × A+ |  |  |  |  |  |  |  |  |  |  |  |  |  |  |  |  |  |  | 0.01 | 0.00 – 0.02 | **<0.001** |
| Random Effects | | | | | | | | | | | | | | | | | | | | | |
| σ^2^ | 0.80 | | | 0.79 | | | 0.80 | | | 0.80 | | | 0.80 | | | 0.79 | | | 0.79 | | |
| τ_00_ | 1.81 _Reggieid_ | | | 1.70 _Reggieid_ | | | 1.82 _Reggieid_ | | | 1.83 _Reggieid_ | | | 1.83 _Reggieid_ | | | 1.70 _Reggieid_ | | | 1.32 _Reggieid_ | | |
| τ_11_ | 0.03 _Reggieid.c60age_ | | | 0.02 _Reggieid.c60age_ | | | 0.03 _Reggieid.c60age_ | | | 0.03 _Reggieid.c60age_ | | | 0.03 _Reggieid.c60age_ | | | 0.02 _Reggieid.c60age_ | | | 0.02 _Reggieid.c60age_ | | |
| ρ_01_ | 0.75 _Reggieid_ | | | 0.77 _Reggieid_ | | | 0.75 _Reggieid_ | | | 0.77 _Reggieid_ | | | 0.75 _Reggieid_ | | | 0.77 _Reggieid_ | | | 0.70 _Reggieid_ | | |
| ICC | 0.87 | | | 0.86 | | | 0.87 | | | 0.87 | | | 0.87 | | | 0.86 | | | 0.81 | | |
| N | 366 _Reggieid_ | | | 366 _Reggieid_ | | | 366 _Reggieid_ | | | 366 _Reggieid_ | | | 366 _Reggieid_ | | | 366 _Reggieid_ | | | 366 _Reggieid_ | | |
| Observations | 1090 | | | 1090 | | | 1090 | | | 1090 | | | 1090 | | | 1090 | | | 1090 | | |
| Marginal R^2^ / Conditional R^2^ | 0.125 / 0.885 | | | 0.235 / 0.891 | | | 0.124 / 0.885 | | | 0.128 / 0.884 | | | 0.124 / 0.885 | | | 0.235 / 0.891 | | | 0.443 / 0.894 | | |
| AICc | 3906.1 | | | 3850.7 | | | 3906.7 | | | 3907.0 | | | 3908.6 | | | 3850.7 | | | 3751.8 | | |
| $\Delta$AICc | 1.33 | | | -54.15 | | | 1.93 | | | 2.22 | | | 3.83 | | | -54.15 | | | -153.01 | | |

*The base model is age fixed effects up to cubic polynomial (retaining highest order significant term and lower order terms; centered at age 60) and random effects. Models 1-5 started with predictor*age included and then removed if NS. Model 6 bring in significant main effects and interactions from Models 1-5, remove NS interactions sequentially (least significant out first) until only significant interactions (and their supporting main effects) or significant main effect (if no corresponding interactions are significant) remain. Model 7 added age*A status up to cubic polynomial age interaction to model 6 and remove NS interactions sequentially until only significant interactions (and their supporting main effects) or significant main effect. ΔAICc is calculated relative to the base model; negative numbers indicate better fit in the expanded model.

**Supplementary Table 8: Sensitivity analyses of plasma biomarkers mixed effects output in all available plasma biomarkers without A status (Aim 2).**

|  | **z_pTau217** | | | **z_ab4240** | | | **z_pTau181** | | | **z_pTau231** | | | **z_GFAP** | | | **z_NFL** | | |
| --- | --- | --- | --- | --- | --- | --- | --- | --- | --- | --- | --- | --- | --- | --- | --- | --- | --- | --- |
| *Predictors* | *Estimates* | *CI* | *p* | *Estimates* | *CI* | *p* | *Estimates* | *CI* | *p* | *Estimates* | *CI* | *p* | *Estimates* | *CI* | *p* | *Estimates* | *CI* | *p* |
| (Intercept) | -0.03 | -0.24 – 0.17 | 0.768 | -0.15 | -0.31 – 0.01 | 0.068 | -0.18 | -0.46 – 0.11 | 0.226 | -0.27 | -0.44 – -0.09 | **0.003** | 0.06 | -0.14 – 0.26 | 0.573 | -0.06 | -0.24 – 0.12 | 0.497 |
| c60age | 0.08 | 0.05 – 0.11 | **<0.001** | -0.02 | -0.04 – -0.01 | **0.003** | 0.04 | 0.02 – 0.05 | **<0.001** | 0.03 | 0.02 – 0.05 | **<0.001** | 0.05 | 0.04 – 0.06 | **<0.001** | 0.08 | 0.07 – 0.10 | **<0.001** |
| c60age^2 | 0.00 | 0.00 – 0.01 | **<0.001** |  |  |  |  |  |  |  |  |  |  |  |  |  |  |  |
| npscore | 0.40 | 0.23 – 0.56 | **<0.001** | -0.18 | -0.27 – -0.08 | **<0.001** | 0.16 | 0.03 – 0.29 | **0.017** | 0.14 | 0.04 – 0.24 | **0.004** | 0.18 | 0.08 – 0.28 | **<0.001** |  |  |  |
| c60age × npscore | 0.03 | 0.01 – 0.06 | **0.001** | -0.01 | -0.02 – -0.00 | **0.024** | 0.01 | 0.00 – 0.03 | **0.046** |  |  |  |  |  |  |  |  |  |
| c60age^2 × npscore | 0.00 | 0.00 – 0.01 | **<0.001** |  |  |  |  |  |  |  |  |  |  |  |  |  |  |  |
| CKD EPI [Stage 2] |  |  |  | 0.27 | 0.07 – 0.47 | **0.009** |  |  |  | 0.49 | 0.27 – 0.70 | **<0.001** | 0.32 | 0.10 – 0.53 | **0.004** | 0.25 | 0.03 – 0.47 | **0.026** |
| CKD EPI [Stage 3] |  |  |  | 0.33 | -0.46 – 1.11 | 0.413 |  |  |  | 0.34 | -0.34 – 1.03 | 0.322 | 1.27 | 0.50 – 2.05 | **0.001** | 1.60 | 0.91 – 2.28 | **<0.001** |
| c60age × CKD EPI  [Stage 2] |  |  |  | -0.03 | -0.05 – -0.01 | **0.003** |  |  |  |  |  |  |  |  |  |  |  |  |
| c60age × CKD EPI  [Stage 3] |  |  |  | -0.00 | -0.08 – 0.07 | 0.963 |  |  |  |  |  |  |  |  |  |  |  |  |
| LIBRA  [Moderate] |  |  |  |  |  |  | 0.26 | -0.07 – 0.60 | 0.124 |  |  |  |  |  |  |  |  |  |
| LIBRA [High] |  |  |  |  |  |  | 0.34 | 0.02 – 0.66 | **0.036** |  |  |  |  |  |  |  |  |  |
| Male |  |  |  |  |  |  |  |  |  |  |  |  | -0.47 | -0.69 – -0.25 | **<0.001** |  |  |  |
| obese |  |  |  |  |  |  |  |  |  |  |  |  | -0.45 | -0.67 – -0.24 | **<0.001** | -0.30 | -0.52 – -0.08 | **0.008** |
| **Random Effects** | | | | | | | | | | | | | | | | | | |
| σ^2^ | 0.82 | | | 0.25 | | | 0.68 | | | 0.66 | | | 0.33 | | | 1.45 | | |
| τ_00_ | 2.15 _Reggieid_ | | | 0.79 _Reggieid_ | | | 1.39 _Reggieid_ | | | 0.89 _Reggieid_ | | | 0.91 _Reggieid_ | | | 0.63 _Reggieid_ | | |
| τ_11_ | 0.02 _Reggieid.c60age_ | | | 0.00 _Reggieid.c60age_ | | |  | | |  | | | 0.00 _Reggieid.c60age_ | | |  | | |
| ρ_01_ | 0.81 _Reggieid_ | | | 0.73 _Reggieid_ | | |  | | |  | | | 0.92 _Reggieid_ | | |  | | |
| ICC | 0.86 | | | 0.78 | | | 0.67 | | | 0.57 | | | 0.80 | | | 0.30 | | |
| N | 412 _Reggieid_ | | | 411 _Reggieid_ | | | 412 _Reggieid_ | | | 411 _Reggieid_ | | | 411 _Reggieid_ | | | 411 _Reggieid_ | | |
| Observations | 1232 | | | 1230 | | | 1226 | | | 1229 | | | 1229 | | | 1230 | | |
| Marginal R^2^ / Conditional R^2^ | 0.225 / 0.891 | | | 0.111 / 0.806 | | | 0.072 / 0.695 | | | 0.089 / 0.613 | | | 0.145 / 0.831 | | | 0.186 / 0.433 | | |

**Supplementary Table 9: Plasma biomarkers mixed effects output in sample with PiB PET 3 level A status in exploratory analysis (Aim 2).**

|  | **z_pTau217** | | | **z_ab4240** | | | **z_pTau181** | | | **z_pTau231** | | | **z_GFAP** | | | **z_NFL** | | |
| --- | --- | --- | --- | --- | --- | --- | --- | --- | --- | --- | --- | --- | --- | --- | --- | --- | --- | --- |
| *Predictors* | *Estimates* | *CI* | *p* | *Estimates* | *CI* | *p* | *Estimates* | *CI* | *p* | *Estimates* | *CI* | *p* | *Estimates* | *CI* | *p* | *Estimates* | *CI* | *p* |
| (Intercept) | -0.16 | -0.37 – 0.04 | 0.125 | -0.06 | -0.25 – 0.14 | 0.569 | -0.35 | -0.60 – -0.11 | **0.005** | -0.24 | -0.43 – -0.05 | **0.016** | -0.04 | -0.28 – 0.20 | 0.744 | -0.13 | -0.32 – 0.06 | 0.192 |
| c60age | 0.04 | 0.01 – 0.07 | **0.003** | -0.02 | -0.04 – -0.00 | **0.038** | 0.04 | 0.02 – 0.05 | **<0.001** | 0.03 | 0.01 – 0.04 | **<0.001** | 0.03 | 0.01 – 0.05 | **0.004** | 0.08 | 0.07 – 0.10 | **<0.001** |
| c60age^2 | 0.00 | -0.00 – 0.00 | 0.080 |  |  |  |  |  |  |  |  |  |  |  |  |  |  |  |
| npscore | 0.19 | 0.02 – 0.35 | **0.025** | -0.07 | -0.19 – 0.04 | 0.220 |  |  |  |  |  |  | 0.14 | 0.01 – 0.26 | **0.031** |  |  |  |
| c60age × npscore |  |  |  | -0.01 | -0.02 – -0.00 | **0.039** |  |  |  |  |  |  |  |  |  |  |  |  |
| 1.14 < PiB DVR <1.19 | 0.07 | -0.74 – 0.88 | 0.861 | -0.10 | -0.61 – 0.40 | 0.696 | 0.25 | -0.30 – 0.80 | 0.381 | -0.15 | -0.67 – 0.37 | 0.572 | -0.29 | -0.90 – 0.33 | 0.363 |  |  |  |
| 1.19 < PiB DVR] | 1.63 | 1.12 – 2.13 | **<0.001** | -0.52 | -0.81 – -0.23 | **<0.001** | 1.10 | 0.80 – 1.40 | **<0.001** | 0.90 | 0.59 – 1.20 | **<0.001** | 0.86 | 0.48 – 1.24 | **<0.001** |  |  |  |
|  |  |  |  |  |  |  |  |  |  |  |  |  |  |  |  |  |  |  |
| c60age × 1.14 < PiB <1.19 | 0.01 | -0.12 – 0.14 | 0.889 |  |  |  |  |  |  |  |  |  | 0.03 | -0.03 – 0.09 | 0.325 |  |  |  |
| c60age × 1.19 < PiB DVR | 0.17 | 0.09 – 0.25 | **<0.001** |  |  |  |  |  |  |  |  |  | 0.04 | 0.00 – 0.07 | **0.037** |  |  |  |
| 1.14 < PiB DVR <1.19 × c60age^2 | 0.00 | -0.01 – 0.01 | 0.654 |  |  |  |  |  |  |  |  |  |  |  |  |  |  |  |
| 1.19 < PiB DVR × c60age^2 | 0.01 | 0.01 – 0.02 | **<0.001** |  |  |  |  |  |  |  |  |  |  |  |  |  |  |  |
| CKD EPI [Stage 2] |  |  |  | 0.23 | -0.01 – 0.47 | 0.055 |  |  |  | 0.35 | 0.12 – 0.58 | **0.003** | 0.30 | 0.04 – 0.55 | **0.023** | 0.33 | 0.10 – 0.56 | **0.005** |
| CKD EPI [Stage 3] |  |  |  | 0.75 | -0.15 – 1.65 | 0.104 |  |  |  | 0.20 | -0.54 – 0.94 | 0.595 | 1.13 | 0.15 – 2.11 | **0.024** | 1.68 | 0.94 – 2.43 | **<0.001** |
| c60age × CKD EPI  [Stage 2] |  |  |  | -0.03 | -0.06 – -0.01 | **0.004** |  |  |  |  |  |  | 0.00 | -0.02 – 0.03 | 0.881 |  |  |  |
| c60age × CKD EPI  [Stage 3] |  |  |  | 0.01 | -0.07 – 0.08 | 0.895 |  |  |  |  |  |  | 0.13 | 0.03 – 0.22 | **0.011** |  |  |  |
| LIBRA  [Moderate] |  |  |  |  |  |  | 0.33 | 0.03 – 0.64 | **0.030** |  |  |  |  |  |  |  |  |  |
| LIBRA [High] |  |  |  |  |  |  | 0.43 | 0.13 – 0.72 | **0.004** |  |  |  |  |  |  |  |  |  |
| Male |  |  |  |  |  |  |  |  |  |  |  |  | -0.42 | -0.69 – -0.16 | **0.002** |  |  |  |
| obese |  |  |  |  |  |  |  |  |  |  |  |  | -0.40 | -0.66 – -0.14 | **0.003** | -0.31 | -0.54 – -0.08 | **0.008** |
| **Random Effects** | | | | | | | | | | | | | | | | | | |
| σ^2^ | 0.86 | | | 0.26 | | | 0.67 | | | 0.70 | | | 0.34 | | | 0.70 | | |
| τ_00_ | 1.28 _Reggieid_ | | | 0.82 _Reggieid_ | | | 0.87 _Reggieid_ | | | 0.72 _Reggieid_ | | | 0.94 _Reggieid_ | | | 0.72 _Reggieid_ | | |
| τ_11_ | 0.02 _Reggieid.c60age_ | | |  | | |  | | |  | | | 0.00 _Reggieid.c60age_ | | |  | | |
| ρ_01_ | 0.72 _Reggieid_ | | |  | | |  | | |  | | | 0.95 _Reggieid_ | | |  | | |
| ICC | 0.81 | | | 0.76 | | | 0.56 | | | 0.51 | | | 0.79 | | | 0.51 | | |
| N | 319 _Reggieid_ | | | 319 _Reggieid_ | | | 319 _Reggieid_ | | | 319 _Reggieid_ | | | 319 _Reggieid_ | | | 319 _Reggieid_ | | |
| Observations | 953 | | | 953 | | | 949 | | | 952 | | | 952 | | | 953 | | |
| Marginal R^2^ / Conditional R^2^ | 0.463 / 0.896 | | | 0.168 / 0.800 | | | 0.169 / 0.638 | | | 0.147 / 0.579 | | | 0.256 / 0.846 | | | 0.261 / 0.635 | | |

**Supplementary Table 10: Plasma Aβ42/40 z-score mixed effects model sets output in people who have A status (Aim 2)**

|  | **1 (predictor=sex)** | | | **2 (predictor=npscore)** | | | **3 (predictor=BMI)** | | | **4 (predictor=CKD EPI)** | | | **5 (predictor=LIBRA)** | | | **6 (combined)** | | | **7 (A status)** | | |
| --- | --- | --- | --- | --- | --- | --- | --- | --- | --- | --- | --- | --- | --- | --- | --- | --- | --- | --- | --- | --- | --- |
| *Predictors* | *Estimates* | *CI* | *p* | *Estimates* | *CI* | *p* | *Estimates* | *CI* | *p* | *Estimates* | *CI* | *p* | *Estimates* | *CI* | *p* | *Estimates* | *CI* | *p* | *Estimates* | *CI* | *p* |
| (Intercept) | 0.01 | -0.12 – 0.14 | 0.889 | -0.01 | -0.15 – 0.12 | 0.823 | -0.01 | -0.15 – 0.12 | 0.830 | -0.21 | -0.38 – -0.04 | **0.014** | 0.01 | -0.20 – 0.22 | 0.931 | -0.04 | -0.23 – 0.15 | 0.669 | -0.01 | -0.20 – 0.18 | 0.893 |
| c60age | -0.04 | -0.05 – -0.03 | **<0.001** | -0.04 | -0.05 – -0.02 | **<0.001** | -0.05 | -0.06 – -0.03 | **<0.001** | -0.03 | -0.05 – -0.02 | **<0.001** | -0.04 | -0.05 – -0.03 | **<0.001** | -0.02 | -0.04 – -0.01 | **0.004** | -0.02 | -0.04 – -0.01 | **0.009** |
| Male | -0.27 | -0.49 – -0.06 | **0.014** |  |  |  |  |  |  |  |  |  |  |  |  | -0.24 | -0.45 – -0.03 | **0.026** | -0.24 | -0.44 – -0.03 | **0.027** |
| npscore |  |  |  | -0.12 | -0.23 – -0.02 | **0.021** |  |  |  |  |  |  |  |  |  | -0.13 | -0.23 – -0.03 | **0.014** | -0.08 | -0.18 – 0.03 | 0.161 |
| c60age × npscore |  |  |  | -0.02 | -0.03 – -0.01 | **0.001** |  |  |  |  |  |  |  |  |  | -0.02 | -0.02 – -0.01 | **0.002** | -0.01 | -0.02 – -0.00 | **0.005** |
| obese |  |  |  |  |  |  | -0.19 | -0.41 – 0.03 | 0.089 |  |  |  |  |  |  |  |  |  |  |  |  |
| CKD EPI [Stage 2] |  |  |  |  |  |  |  |  |  | 0.25 | 0.02 – 0.48 | **0.033** |  |  |  | 0.22 | 0.00 – 0.45 | **0.050** | 0.24 | 0.02 – 0.46 | **0.029** |
| CKD EPI [Stage 3] |  |  |  |  |  |  |  |  |  | 0.49 | -0.40 – 1.38 | 0.284 |  |  |  | 0.41 | -0.46 – 1.28 | 0.361 | 0.42 | -0.44 – 1.28 | 0.341 |
| c60age × CKD EPI  [Stage 2] |  |  |  |  |  |  |  |  |  | -0.02 | -0.04 – -0.00 | **0.031** |  |  |  | -0.03 | -0.05 – -0.00 | **0.016** | -0.02 | -0.05 – -0.00 | **0.020** |
| c60age × CKD EPI  [Stage 3] |  |  |  |  |  |  |  |  |  | 0.02 | -0.06 – 0.09 | 0.634 |  |  |  | 0.01 | -0.07 – 0.09 | 0.799 | 0.01 | -0.07 – 0.08 | 0.843 |
| LIBRA  [Moderate] |  |  |  |  |  |  |  |  |  |  |  |  | -0.14 | -0.41 – 0.14 | 0.321 |  |  |  |  |  |  |
| LIBRA [High] |  |  |  |  |  |  |  |  |  |  |  |  | -0.10 | -0.37 – 0.16 | 0.437 |  |  |  |  |  |  |
| A+ |  |  |  |  |  |  |  |  |  |  |  |  |  |  |  |  |  |  | -0.49 | -0.76 – -0.22 | **<0.001** |
| **Random Effects** | | | | | | | | | | | | | | | | | | | | | |
| σ^2^ | 0.25 | | | 0.25 | | | 0.25 | | | 0.25 | | | 0.25 | | | 0.25 | | | 0.25 | | |
| τ_00_ | 0.93 _Reggieid_ | | | 0.88 _Reggieid_ | | | 0.94 _Reggieid_ | | | 0.92 _Reggieid_ | | | 0.94 _Reggieid_ | | | 0.85 _Reggieid_ | | | 0.83 _Reggieid_ | | |
| ICC | 0.78 | | | 0.78 | | | 0.79 | | | 0.78 | | | 0.79 | | | 0.77 | | | 0.77 | | |
| N | 374 _Reggieid_ | | | 374 _Reggieid_ | | | 374 _Reggieid_ | | | 374 _Reggieid_ | | | 374 _Reggieid_ | | | 374 _Reggieid_ | | | 374 _Reggieid_ | | |
| Observations | 1112 | | | 1112 | | | 1112 | | | 1112 | | | 1112 | | | 1112 | | | 1112 | | |
| Marginal R^2^ / Conditional R^2^ | 0.087 / 0.804 | | | 0.110 / 0.802 | | | 0.078 / 0.803 | | | 0.081 / 0.802 | | | 0.076 / 0.804 | | | 0.130 / 0.801 | | | 0.164 / 0.804 | | |
| AICc | 2549.5 | | | 2531.2 | | | 2552.7 | | | 2551.7 | | | 2556.6 | | | 2526.3 | | | 2515.9 | | |
| $\Delta$AICc | -4.08 | | | -22.38 | | | -0.89 | | | -1.89 | | | 3.00 | | | -27.34 | | | -37.76 | | |

***The base model is age fixed effects up to cubic polynomial (retaining highest order significant term and lower order terms; centered at age 60) and random effects. Models 1-5 started with predictor*age included and then removed if NS. Model 6 bring in significant main effects and interactions from Models 1-5, remove NS interactions sequentially (least significant out first) until only significant interactions (and their supporting main effects) or significant main effect (if no corresponding interactions are significant) remain. Model 7 added age*A status up to cubic polynomial age interaction to model 6 and remove NS interactions sequentially until only significant interactions (and their supporting main effects) or significant main effect.** $\boldsymbol{\Delta}$**AICc is calculated relative to the base model; negative numbers indicate better fit in the expanded model.**

**Supplementary Table 11: Plasma p-tau181 z-score mixed effects model sets output in people who have A status (Aim 2)**

|  | **1 (predictor=sex)** | | | **2 (predictor=npscore)** | | | **3 (predictor=BMI)** | | | **4 (predictor=CKD EPI)** | | | **5 (predictor=LIBRA)** | | | **6 (combined)** | | | **7 (A status)** | | |
| --- | --- | --- | --- | --- | --- | --- | --- | --- | --- | --- | --- | --- | --- | --- | --- | --- | --- | --- | --- | --- | --- |
| *Predictors* | *Estimates* | *CI* | *p* | *Estimates* | *CI* | *p* | *Estimates* | *CI* | *p* | *Estimates* | *CI* | *p* | *Estimates* | *CI* | *p* | *Estimates* | *CI* | *p* | *Estimates* | *CI* | *p* |
| (Intercept) | 0.07 | -0.10 – 0.23 | 0.442 | -0.02 | -0.18 – 0.15 | 0.851 | 0.13 | -0.04 – 0.30 | 0.132 | 0.03 | -0.17 – 0.24 | 0.766 | -0.12 | -0.38 – 0.15 | 0.389 | -0.29 | -0.56 – -0.01 | **0.043** | -0.35 | -0.61 – -0.09 | **0.008** |
| c60age | 0.05 | 0.03 – 0.06 | **<0.001** | 0.05 | 0.03 – 0.06 | **<0.001** | 0.05 | 0.03 – 0.06 | **<0.001** | 0.04 | 0.03 – 0.06 | **<0.001** | 0.04 | 0.03 – 0.06 | **<0.001** | 0.05 | 0.03 – 0.06 | **<0.001** | 0.04 | 0.02 – 0.05 | **<0.001** |
| Male | 0.15 | -0.12 – 0.43 | 0.265 |  |  |  |  |  |  |  |  |  |  |  |  |  |  |  |  |  |  |
| npscore |  |  |  | 0.20 | 0.08 – 0.32 | **0.001** |  |  |  |  |  |  |  |  |  | 0.21 | 0.09 – 0.33 | **0.001** |  |  |  |
| obese |  |  |  |  |  |  | -0.04 | -0.32 – 0.23 | 0.746 |  |  |  |  |  |  |  |  |  |  |  |  |
| CKD EPI [Stage 2] |  |  |  |  |  |  |  |  |  | 0.15 | -0.12 – 0.42 | 0.267 |  |  |  |  |  |  |  |  |  |
| CKD EPI [Stage 3] |  |  |  |  |  |  |  |  |  | 0.20 | -0.69 – 1.10 | 0.656 |  |  |  |  |  |  |  |  |  |
| LIBRA  [Moderate] |  |  |  |  |  |  |  |  |  |  |  |  | 0.27 | -0.07 – 0.60 | 0.123 | 0.32 | -0.02 – 0.65 | 0.062 | 0.40 | 0.08 – 0.72 | **0.015** |
| LIBRA [High] |  |  |  |  |  |  |  |  |  |  |  |  | 0.35 | 0.02 – 0.67 | **0.036** | 0.38 | 0.06 – 0.70 | **0.020** | 0.42 | 0.12 – 0.73 | **0.007** |
| A+ |  |  |  |  |  |  |  |  |  |  |  |  |  |  |  |  |  |  | 1.05 | 0.73 – 1.36 | **<0.001** |
| **Random Effects** | | | | | | | | | | | | | | | | | | | | | |
| σ^2^ | 0.62 | | | 0.62 | | | 0.62 | | | 0.62 | | | 0.62 | | | 0.62 | | | 0.62 | | |
| τ_00_ | 1.36 _Reggieid_ | | | 1.32 _Reggieid_ | | | 1.36 _Reggieid_ | | | 1.37 _Reggieid_ | | | 1.35 _Reggieid_ | | | 1.30 _Reggieid_ | | | 1.20 _Reggieid_ | | |
| ICC | 0.69 | | | 0.68 | | | 0.69 | | | 0.69 | | | 0.69 | | | 0.68 | | | 0.66 | | |
| N | 374 _Reggieid_ | | | 374 _Reggieid_ | | | 374 _Reggieid_ | | | 374 _Reggieid_ | | | 374 _Reggieid_ | | | 374 _Reggieid_ | | | 374 _Reggieid_ | | |
| Observations | 1107 | | | 1107 | | | 1107 | | | 1107 | | | 1107 | | | 1107 | | | 1107 | | |
| Marginal R^2^ / Conditional R^2^ | 0.049 / 0.702 | | | 0.064 / 0.700 | | | 0.047 / 0.702 | | | 0.051 / 0.704 | | | 0.056 / 0.703 | | | 0.076 / 0.702 | | | 0.138 / 0.707 | | |
| AICc | 3360.9 | | | 3351.8 | | | 3362.1 | | | 3362.9 | | | 3359.7 | | | 3350.1 | | | 3321.7 | | |
| $\Delta$AICc | 0.77 | | | -8.38 | | | 1.91 | | | 2.74 | | | -0.51 | | | -10.12 | | | -38.44 | | |

***The base model is age fixed effects up to cubic polynomial (retaining highest order significant term and lower order terms; centered at age 60) and random effects. Models 1-5 started with predictor*age included and then removed if NS. Model 6 bring in significant main effects and interactions from Models 1-5, remove NS interactions sequentially (least significant out first) until only significant interactions (and their supporting main effects) or significant main effect (if no corresponding interactions are significant) remain. Model 7 added age*A status up to cubic polynomial age interaction to model 6 and remove NS interactions sequentially until only significant interactions (and their supporting main effects) or significant main effect.** $\boldsymbol{\Delta}$**AICc is calculated relative to the base model; negative numbers indicate better fit in the expanded model.**

**Supplementary Table 12: Plasma p-tau231 z-score mixed effects model sets output in people who have A status (Aim 2)**

|  | **1 (predictor=sex)** | | | **2 (predictor=npscore)** | | | **3 (predictor=BMI)** | | | **4 (predictor=CKD EPI)** | | | **5 (predictor=LIBRA)** | | | **6 (combined)** | | | **7 (A status)** | | |
| --- | --- | --- | --- | --- | --- | --- | --- | --- | --- | --- | --- | --- | --- | --- | --- | --- | --- | --- | --- | --- | --- |
| *Predictors* | *Estimates* | *CI* | *p* | *Estimates* | *CI* | *p* | *Estimates* | *CI* | *p* | *Estimates* | *CI* | *p* | *Estimates* | *CI* | *p* | *Estimates* | *CI* | *p* | *Estimates* | *CI* | *p* |
| (Intercept) | 0.10 | -0.05 – 0.24 | 0.190 | -0.02 | -0.16 – 0.11 | 0.747 | 0.09 | -0.05 – 0.23 | 0.219 | -0.16 | -0.33 – 0.01 | 0.064 | -0.06 | -0.28 – 0.16 | 0.604 | -0.26 | -0.43 – -0.08 | **0.005** | -0.27 | -0.43 – -0.11 | **0.001** |
| c60age | 0.03 | 0.01 – 0.04 | **0.001** | 0.04 | 0.03 – 0.05 | **<0.001** | 0.04 | 0.02 – 0.05 | **<0.001** | 0.03 | 0.02 – 0.05 | **<0.001** | 0.04 | 0.02 – 0.05 | **<0.001** | 0.03 | 0.02 – 0.05 | **<0.001** | 0.03 | 0.01 – 0.04 | **<0.001** |
| Male | -0.07 | -0.33 – 0.18 | 0.575 |  |  |  |  |  |  |  |  |  |  |  |  |  |  |  |  |  |  |
| c60age × Male | 0.03 | 0.00 – 0.05 | **0.049** |  |  |  |  |  |  |  |  |  |  |  |  |  |  |  |  |  |  |
| npscore |  |  |  | 0.14 | 0.04 – 0.24 | **0.005** |  |  |  |  |  |  |  |  |  | 0.14 | 0.05 – 0.24 | **0.004** |  |  |  |
| obese |  |  |  |  |  |  | -0.05 | -0.27 – 0.18 | 0.686 |  |  |  |  |  |  |  |  |  |  |  |  |
| CKD EPI [Stage 2] |  |  |  |  |  |  |  |  |  | 0.43 | 0.21 – 0.64 | **<0.001** |  |  |  | 0.43 | 0.21 – 0.64 | **<0.001** | 0.39 | 0.19 – 0.60 | **<0.001** |
| CKD EPI [Stage 3] |  |  |  |  |  |  |  |  |  | 0.32 | -0.40 – 1.04 | 0.377 |  |  |  | 0.36 | -0.35 – 1.07 | 0.321 | 0.38 | -0.30 – 1.06 | 0.275 |
| LIBRA  [Moderate] |  |  |  |  |  |  |  |  |  |  |  |  | 0.16 | -0.12 – 0.44 | 0.269 |  |  |  |  |  |  |
| LIBRA [High] |  |  |  |  |  |  |  |  |  |  |  |  | 0.19 | -0.08 – 0.46 | 0.160 |  |  |  |  |  |  |
| A+ |  |  |  |  |  |  |  |  |  |  |  |  |  |  |  |  |  |  | 0.88 | 0.62 – 1.14 | **<0.001** |
| **Random Effects** | | | | | | | | | | | | | | | | | | | | | |
| σ^2^ | 0.64 | | | 0.64 | | | 0.64 | | | 0.64 | | | 0.64 | | | 0.64 | | | 0.64 | | |
| τ_00_ | 0.85 _Reggieid_ | | | 0.82 _Reggieid_ | | | 0.85 _Reggieid_ | | | 0.81 _Reggieid_ | | | 0.84 _Reggieid_ | | | 0.79 _Reggieid_ | | | 0.71 _Reggieid_ | | |
| ICC | 0.57 | | | 0.56 | | | 0.57 | | | 0.56 | | | 0.57 | | | 0.55 | | | 0.53 | | |
| N | 374 _Reggieid_ | | | 374 _Reggieid_ | | | 374 _Reggieid_ | | | 374 _Reggieid_ | | | 374 _Reggieid_ | | | 374 _Reggieid_ | | | 374 _Reggieid_ | | |
| Observations | 1111 | | | 1111 | | | 1111 | | | 1111 | | | 1111 | | | 1111 | | | 1111 | | |
| Marginal R^2^ / Conditional R^2^ | 0.048 / 0.590 | | | 0.055 / 0.584 | | | 0.042 / 0.587 | | | 0.073 / 0.591 | | | 0.046 / 0.587 | | | 0.086 / 0.590 | | | 0.146 / 0.595 | | |
| AICc | 3254.8 | | | 3249.0 | | | 3256.7 | | | 3244.2 | | | 3256.8 | | | 3237.9 | | | 3203.2 | | |
| $\Delta$AICc | -0.052 | | | -5.79 | | | 1.85 | | | -10.66 | | | 1.94 | | | -16.90 | | | -51.62 | | |

***The base model is age fixed effects up to cubic polynomial (retaining highest order significant term and lower order terms; centered at age 60) and random effects. Models 1-5 started with predictor*age included and then removed if NS. Model 6 bring in significant main effects and interactions from Models 1-5, remove NS interactions sequentially (least significant out first) until only significant interactions (and their supporting main effects) or significant main effect (if no corresponding interactions are significant) remain. Model 7 added age*A status up to cubic polynomial age interaction to model 6 and remove NS interactions sequentially until only significant interactions (and their supporting main effects) or significant main effect.** $\boldsymbol{\Delta}$**AICc is calculated relative to the base model; negative numbers indicate better fit in the expanded model.**

**Supplementary Table 13: Plasma GFAP z-score mixed effects model sets output in people who have A status (Aim 2)**

|  | **1 (predictor=sex)** | | | **2 (predictor=npscore)** | | | **3 (predictor=BMI)** | | | **4 (predictor=CKD EPI)** | | | **5 (predictor=LIBRA)** | | | **6 (combined)** | | | **7 (A status)** | | |
| --- | --- | --- | --- | --- | --- | --- | --- | --- | --- | --- | --- | --- | --- | --- | --- | --- | --- | --- | --- | --- | --- |
| *Predictors* | *Estimates* | *CI* | *p* | *Estimates* | *CI* | *p* | *Estimates* | *CI* | *p* | *Estimates* | *CI* | *p* | *Estimates* | *CI* | *p* | *Estimates* | *CI* | *p* | *Estimates* | *CI* | *p* |
| Intercept) | 0.25 | 0.11 – 0.39 | **0.001** | -0.04 | -0.18 – 0.10 | 0.537 | 0.22 | 0.07 – 0.36 | **0.004** | -0.10 | -0.28 – 0.07 | 0.253 | 0.14 | -0.09 – 0.38 | 0.236 | 0.08 | -0.13 – 0.30 | 0.447 | 0.06 | -0.14 – 0.26 | 0.566 |
| c60age | 0.05 | 0.04 – 0.06 | **<0.001** | 0.05 | 0.04 – 0.06 | **<0.001** | 0.05 | 0.03 – 0.06 | **<0.001** | 0.04 | 0.03 – 0.06 | **<0.001** | 0.05 | 0.04 – 0.06 | **<0.001** | 0.05 | 0.03 – 0.06 | **<0.001** | 0.03 | 0.01 – 0.05 | **0.005** |
| Male | -0.53 | -0.78 – -0.28 | **<0.001** |  |  |  |  |  |  |  |  |  |  |  |  | -0.50 | -0.74 – -0.26 | **<0.001** | -0.53 | -0.76 – -0.30 | **<0.001** |
| npscore |  |  |  | 0.19 | 0.08 – 0.30 | **0.001** |  |  |  |  |  |  |  |  |  | 0.20 | 0.10 – 0.31 | **<0.001** |  |  |  |
| obese |  |  |  |  |  |  | -0.38 | -0.63 – -0.12 | **0.003** |  |  |  |  |  |  | -0.45 | -0.68 – -0.21 | **<0.001** | -0.39 | -0.62 – -0.16 | **0.001** |
| CKD EPI [Stage 2] |  |  |  |  |  |  |  |  |  | 0.31 | 0.06 – 0.55 | **0.014** |  |  |  | 0.30 | 0.06 – 0.53 | **0.012** | 0.27 | 0.04 – 0.49 | **0.021** |
| CKD EPI [Stage 3] |  |  |  |  |  |  |  |  |  | 1.45 | 0.55 – 2.36 | **0.002** |  |  |  | 1.36 | 0.49 – 2.23 | **0.002** | 1.01 | 0.10 – 1.93 | **0.030** |
| LIBRA  [Moderate] |  |  |  |  |  |  |  |  |  |  |  |  | -0.03 | -0.35 – 0.29 | 0.855 |  |  |  |  |  |  |
| LIBRA [High] |  |  |  |  |  |  |  |  |  |  |  |  | -0.12 | -0.43 – 0.18 | 0.435 |  |  |  |  |  |  |
| A+ |  |  |  |  |  |  |  |  |  |  |  |  |  |  |  |  |  |  | 0.92 | 0.59 – 1.25 | **<0.001** |
| c60age × A+ |  |  |  |  |  |  |  |  |  |  |  |  |  |  |  |  |  |  | 0.04 | 0.01 – 0.07 | **0.019** |
| c60age × CKD EPI  [Stage 2] |  |  |  |  |  |  |  |  |  |  |  |  |  |  |  |  |  |  | 0.01 | -0.02 – 0.03 | 0.588 |
| c60age × CKD EPI  [Stage 3] |  |  |  |  |  |  |  |  |  |  |  |  |  |  |  |  |  |  | 0.10 | 0.00 – 0.19 | **0.044** |
| **Random Effects** | | | | | | | | | | | | | | | | | | | | | |
| σ^2^ | 0.34 | | | 0.34 | | | 0.34 | | | 0.34 | | | 0.34 | | | 0.34 | | | 0.34 | | |
| τ_00_ | 1.00 _Reggieid_ | | | 1.05 _Reggieid_ | | | 1.07 _Reggieid_ | | | 1.06 _Reggieid_ | | | 1.08 _Reggieid_ | | | 0.94 _Reggieid_ | | | 0.87 _Reggieid_ | | |
| τ_11_ | 0.00 _Reggieid.c60age_ | | | 0.00 _Reggieid.c60age_ | | | 0.00 _Reggieid.c60age_ | | | 0.00 _Reggieid.c60age_ | | | 0.00 _Reggieid.c60age_ | | | 0.00 _Reggieid.c60age_ | | | 0.00 _Reggieid.c60age_ | | |
| ρ_01_ | 0.71 _Reggieid_ | | | 0.80 _Reggieid_ | | | 0.71 _Reggieid_ | | | 0.74 _Reggieid_ | | | 0.69 _Reggieid_ | | | 0.86 _Reggieid_ | | | 0.79 _Reggieid_ | | |
| ICC | 0.82 | | | 0.82 | | | 0.83 | | | 0.82 | | | 0.83 | | | 0.80 | | | 0.79 | | |
| N | 374 _Reggieid_ | | | 374 _Reggieid_ | | | 374 _Reggieid_ | | | 374 _Reggieid_ | | | 374 _Reggieid_ | | | 374 _Reggieid_ | | | 374 _Reggieid_ | | |
| Observations | 1111 | | | 1111 | | | 1111 | | | 1111 | | | 1111 | | | 1111 | | | 1111 | | |
| Marginal R^2^ / Conditional R^2^ | 0.083 / 0.837 | | | 0.070 / 0.834 | | | 0.070 / 0.837 | | | 0.088 / 0.838 | | | 0.054 / 0.838 | | | 0.148 / 0.833 | | | 0.250 / 0.844 | | |
| AICc | 2934.4 | | | 2940.7 | | | 2942.9 | | | 2939.8 | | | 2952.8 | | | 2905.7 | | | 2873.0 | | |
| $\Delta$AICc | -15.0 | | | -8.75 | | | -6.53 | | | -9.65 | | | 3.32 | | | -43.78 | | | -76.43 | | |

***The base model is age fixed effects up to cubic polynomial (retaining highest order significant term and lower order terms; centered at age 60) and random effects. Models 1-5 started with predictor*age included and then removed if NS. Model 6 bring in significant main effects and interactions from Models 1-5, remove NS interactions sequentially (least significant out first) until only significant interactions (and their supporting main effects) or significant main effect (if no corresponding interactions are significant) remain. Model 7 added age*A status up to cubic polynomial age interaction to model 6 and remove NS interactions sequentially until only significant interactions (and their supporting main effects) or significant main effect.** $\boldsymbol{\Delta}$**AICc is calculated relative to the base model; negative numbers indicate better fit in the expanded model.**

**Supplementary Table 14: Plasma Nfl z-score mixed effects model sets output in people who have A status (Aim 2)**

|  | **1 (predictor=sex)** | | | **2 (predictor=npscore)** | | | **3 (predictor=BMI)** | | | **4 (predictor=CKD EPI)** | | | **5 (predictor=LIBRA)** | | | **6 (combined)** | | | **7 (A status)** | | |
| --- | --- | --- | --- | --- | --- | --- | --- | --- | --- | --- | --- | --- | --- | --- | --- | --- | --- | --- | --- | --- | --- |
| *Predictors* | *Estimates* | *CI* | *p* | *Estimates* | *CI* | *p* | *Estimates* | *CI* | *p* | *Estimates* | *CI* | *p* | *Estimates* | *CI* | *p* | *Estimates* | *CI* | *p* | *Estimates* | *CI* | *p* |
| (Intercept) | 0.04 | -0.11 – 0.20 | 0.580 | -0.04 | -0.20 – 0.11 | 0.578 | 0.09 | -0.07 – 0.24 | 0.287 | -0.15 | -0.33 – 0.03 | 0.104 | -0.07 | -0.31 – 0.17 | 0.574 | -0.06 | -0.26 – 0.13 | 0.537 | -0.06 | -0.26 – 0.13 | 0.537 |
| c60age | 0.09 | 0.07 – 0.11 | **<0.001** | 0.09 | 0.08 – 0.11 | **<0.001** | 0.09 | 0.07 – 0.10 | **<0.001** | 0.08 | 0.07 – 0.10 | **<0.001** | 0.09 | 0.07 – 0.11 | **<0.001** | 0.08 | 0.07 – 0.10 | **<0.001** | 0.08 | 0.07 – 0.10 | **<0.001** |
| Male | -0.16 | -0.40 – 0.08 | 0.191 |  |  |  |  |  |  |  |  |  |  |  |  |  |  |  |  |  |  |
| npscore |  |  |  | 0.05 | -0.06 – 0.16 | 0.367 |  |  |  |  |  |  |  |  |  |  |  |  |  |  |  |
| obese |  |  |  |  |  |  | -0.27 | -0.51 – -0.03 | **0.030** |  |  |  |  |  |  | -0.28 | -0.51 – -0.04 | **0.023** | -0.28 | -0.51 – -0.04 | **0.023** |
| CKD EPI [Stage 2] |  |  |  |  |  |  |  |  |  | 0.23 | -0.01 – 0.46 | 0.060 |  |  |  | 0.24 | 0.01 – 0.48 | **0.041** | 0.24 | 0.01 – 0.48 | **0.041** |
| CKD EPI [Stage 3] |  |  |  |  |  |  |  |  |  | 1.49 | 0.72 – 2.26 | **<0.001** |  |  |  | 1.48 | 0.72 – 2.25 | **<0.001** | 1.48 | 0.72 – 2.25 | **<0.001** |
| LIBRA  [Moderate] |  |  |  |  |  |  |  |  |  |  |  |  | 0.10 | -0.20 – 0.41 | 0.503 |  |  |  |  |  |  |
| LIBRA [High] |  |  |  |  |  |  |  |  |  |  |  |  | 0.06 | -0.23 – 0.35 | 0.681 |  |  |  |  |  |  |
| **Random Effects** | | | | | | | | | | | | | | | | | | | | | |
| σ^2^ | 1.59 | | | 1.59 | | | 1.59 | | | 1.59 | | | 1.59 | | | 1.59 | | | 1.59 | | |
| τ_00_ | 0.70 _Reggieid_ | | | 0.71 _Reggieid_ | | | 0.69 _Reggieid_ | | | 0.66 _Reggieid_ | | | 0.71 _Reggieid_ | | | 0.64 _Reggieid_ | | | 0.64 _Reggieid_ | | |
| ICC | 0.31 | | | 0.31 | | | 0.30 | | | 0.29 | | | 0.31 | | | 0.29 | | | 0.29 | | |
| N | 374 _Reggieid_ | | | 374 _Reggieid_ | | | 374 _Reggieid_ | | | 374 _Reggieid_ | | | 374 _Reggieid_ | | | 374 _Reggieid_ | | | 374 _Reggieid_ | | |
| Observations | 1112 | | | 1112 | | | 1112 | | | 1112 | | | 1112 | | | 1112 | | | 1112 | | |
| Marginal R^2^ / Conditional R^2^ | 0.145 / 0.407 | | | 0.144 / 0.407 | | | 0.149 / 0.407 | | | 0.164 / 0.409 | | | 0.144 / 0.409 | | | 0.170 / 0.408 | | | 0.170 / 0.408 | | |
| AICc | 3985.3 | | | 3986.2 | | | 3982.4 | | | 3973.4 | | | 3988.6 | | | 3970.2 | | | 3970.2 | | |
| $\Delta$AICc | 0.29 | | | 1.20 | | | -2.68 | | | -11.62 | | | 3.58 | | | -14.83 | | | -14.83 | | |

***The base model is age fixed effects up to cubic polynomial (retaining highest order significant term and lower order terms; centered at age 60) and random effects. Models 1-5 started with predictor*age included and then removed if NS. Model 6 bring in significant main effects and interactions from Models 1-5, remove NS interactions sequentially (least significant out first) until only significant interactions (and their supporting main effects) or significant main effect (if no corresponding interactions are significant) remain. Model 7 added age*A status up to cubic polynomial age interaction to model 6 and remove NS interactions sequentially until only significant interactions (and their supporting main effects) or significant main effect.** $\boldsymbol{\Delta}$**AICc is calculated relative to the base model; negative numbers indicate better fit in the expanded model.**

**Supplementary Table 15: Interaction simple slopes summary**

|  |  | Simple slope (se) for p-tau217 Value representing: | | | Effect size for pairwise comparisons  (BF indicates significant pairwise diff) | | |
| --- | --- | --- | --- | --- | --- | --- | --- |
| outcome | Age | Low (L) | Intermediate (I) | High (H) | L-I | L-H | I-H |
| PACC3 | 60 | -0.035 (0.005) | -0.043 (0.005) | -0.061 (0.009) | **0.02** | **0.08** | **0.05** |
|  | 65 | -0.044 (0.005) | -0.055 (0.005) | -0.080 (0.007) | **0.03** | **0.11** | **0.08** |
|  | 70 | -0.063 (0.006) | -0.077 (0.006) | -0.110 (0.008) | **0.04** | **0.14** | **0.10** |
| Exec Fnc | 60 | -0.065 (0.006) | -0.073 (0.006) | -0.091 (0.008) | **0.02** | **0.08** | **0.06** |
|  | 65 | -0.075 (0.007) | -0.082 (0.007) | -0.101 (0.008) | **0.02** | **0.08** | **0.06** |
|  | 70 | -0.085 (0.008) | -0.092 (0.008) | -0.111 (0.009) | **0.02** | **0.08** | **0.06** |
| Imm Memory | 60 | -0.041 (0.007) | -0.055 (0.007) | -0.091 (0.012) | **0.03** | **0.10** | **0.07** |
|  | 65 | -0.045 (0.007) | -0.065 (0.007) | -0.114 (0.010) | **0.04** | **0.14** | **0.10** |
|  | 70 | -0.061 (0.009) | -0.086 (0.009) | -0.148 (0.013) | **0.05** | **0.18** | **0.13** |
| Delayed | 60 | -0.027 (0.008) | -0.046 (0.007) | -0.090 (0.013) | **0.04** | **0.13** | **0.19** |
|  | 65 | -0.033 (0.008) | -0.056 (0.007) | -0.114 (0.011) | **0.05** | **0.17** | **0.12** |
|  | 70 | -0.050 (0.010) | -0.079 (0.009) | -0.149 (0.013) | **0.06** | **0.21** | **0.15** |
| CDR Sum of Box | 60 | 0.003 (0.005) | -0.005 (0.005) | -0.023 (0.012) | 0.02 | 0.06 | 0.04 |
|  | 65 | 0.003 (0.003) | 0.008 (0.003) | 0.021 (0.008) | -0.01 | -0.04 | -0.03 |
|  | 70 | 0.003 (0.005) | 0.021 (0.004) | 0.066 (0.008) | **-0.04** | **-0.15** | **-0.11** |

**Supplementary Table 16: PACC3 mixed effects output in sensitivity analysis.**

|  | **Model 1** | | | **Model 7** | | |
| --- | --- | --- | --- | --- | --- | --- |
| *Predictors* | *Estimates* | *CI* | *p* | *Estimates* | *CI* | *p* |
| (Intercept) | 0.00 | -0.14 – 0.14 | 0.978 | 0.00 | -0.14 – 0.14 | 0.989 |
| Male | -0.52 | -0.64 – -0.39 | **<0.001** | -0.51 | -0.63 – -0.38 | **<0.001** |
| no BA | -0.20 | -0.33 – -0.06 | **0.005** | -0.20 | -0.33 – -0.06 | **0.005** |
| Practice | 0.09 | 0.07 – 0.12 | **<0.001** | 0.09 | 0.07 – 0.12 | **<0.001** |
| WRAT3 | 0.02 | 0.01 – 0.03 | **<0.001** | 0.02 | 0.01 – 0.03 | <0.001 |
| c60age | -0.04 | -0.05 – -0.03 | **<0.001** | -0.04 | -0.05 – -0.03 | **<0.001** |
| c60age^2 | -0.00 | -0.00 – 0.00 | 0.077 | -0.00 | -0.00 – -0.00 | 0.037 |
| c60age^3 | -0.00 | -0.00 – -0.00 | **<0.001** | -0.00 | -0.00 – -0.00 | 0.011 |
| z pTau217 | -0.00 | -0.03 – 0.03 | 0.875 | -0.01 | -0.04 – 0.02 | 0.582 |
| c60age × z pTau217 | -0.01 | -0.01 – -0.00 | **0.002** | -0.01 | -0.01 – -0.00 | **0.003** |
| z pTau217 × c60age^2 | -0.00 | -0.00 – -0.00 | **0.041** |  |  |  |
| z GFAP |  |  |  | 0.03 | -0.03 – 0.08 | 0.339 |
| c60age × z GFAP |  |  |  | 0.00 | -0.01 – 0.01 | 0.665 |
| z GFAP × c60age^2 |  |  |  | -0.00 | -0.00 – -0.00 | **0.002** |
| **Random Effects** | | | | | | |
| σ^2^ | 0.11 | | | 0.11 | | |
| τ_00_ | 0.29 _Reggieid_ | | | 0.29 _Reggieid_ | | |
| τ_11_ | 0.00 _Reggieid.c60age_ | | | 0.00 _Reggieid.c60age_ | | |
| ρ_01_ | -0.00 _Reggieid_ | | | 0.01 _Reggieid_ | | |
| ICC | 0.76 | | | 0.76 | | |
| N | 412 _Reggieid_ | | | 412 _Reggieid_ | | |
| Observations | 1973 | | | 1973 | | |
| Marginal R^2^ / Conditional R^2^ | 0.352 / 0.842 | | | 0.353 / 0.842 | | |
| AICc | 2600.4 | | | 2591.2 | | |
| $\Delta$AICc |  | | | -9.2 | | |

*We added terms sequentially to model 1 of table 5, beginning with the biomarker terms from the best-fitting model from models 2-6 of table 5, retaining significant interactions or main effects before comparing with the Model 1 AICc. If ∆AICc showed improved model fit, we continued to the next best model from Models 2-6 and added terms from that model. Only biomarkers from models that improved model fit over the base model were considered for this process.

**Supplementary Table 17: EF mixed effects output.**

|  | **1(predictor= p-tau217)** | | | **2(predictor= Aβ42/40)** | | | **3(predictor= p-tau181)** | | | **4(predictor= p-tau231)** | | | **5(predictor= GFAP)** | | | **6(predictor= Nfl)** | | |
| --- | --- | --- | --- | --- | --- | --- | --- | --- | --- | --- | --- | --- | --- | --- | --- | --- | --- | --- |
| *Predictors* | *Estimates* | *CI* | *p* | *Estimates* | *CI* | *p* | *Estimates* | *CI* | *p* | *Estimates* | *CI* | *p* | *Estimates* | *CI* | *p* | *Estimates* | *CI* | *p* |
| (Intercept) | -0.14 | -0.31 – 0.04 | 0.125 | -0.17 | -0.34 – 0.01 | 0.062 | -0.16 | -0.33 – 0.01 | 0.060 | -0.15 | -0.33 – 0.02 | 0.084 | -0.15 | -0.33 – 0.03 | 0.100 | -0.16 | -0.34 – 0.02 | 0.085 |
| c60age | -0.07 | -0.08 – -0.05 | **<0.001** | -0.07 | -0.08 – -0.06 | **<0.001** | -0.07 | -0.08 – -0.06 | **<0.001** | -0.07 | -0.08 – -0.06 | **<0.001** | -0.07 | -0.08 – -0.06 | **<0.001** | -0.07 | -0.08 – -0.06 | **<0.001** |
| z p-tau217 | -0.03 | -0.07 – 0.01 | 0.097 |  |  |  |  |  |  |  |  |  |  |  |  |  |  |  |
| c60age^2 | -0.00 | -0.00 – -0.00 | **<0.001** | -0.00 | -0.00 – -0.00 | **<0.001** | -0.00 | -0.00 – -0.00 | **<0.001** | -0.00 | -0.00 – -0.00 | **<0.001** | -0.00 | -0.00 – -0.00 | **<0.001** | -0.00 | -0.00 – -0.00 | **<0.001** |
| gender f [Male] | -0.23 | -0.39 – -0.07 | **0.005** | -0.23 | -0.39 – -0.07 | **0.004** | -0.22 | -0.37 – -0.06 | **0.007** | -0.24 | -0.40 – -0.08 | **0.003** | -0.25 | -0.41 – -0.08 | **0.003** | -0.24 | -0.40 – -0.08 | **0.003** |
| BA f [no BA] | -0.04 | -0.22 – 0.14 | 0.679 | -0.03 | -0.21 – 0.15 | 0.741 | -0.01 | -0.19 – 0.16 | 0.870 | -0.03 | -0.21 – 0.14 | 0.715 | -0.03 | -0.21 – 0.15 | 0.733 | -0.04 | -0.22 – 0.14 | 0.667 |
| practice | 0.09 | 0.06 – 0.12 | **<0.001** | 0.10 | 0.07 – 0.13 | **<0.001** | 0.10 | 0.06 – 0.13 | **<0.001** | 0.09 | 0.06 – 0.12 | **<0.001** | 0.09 | 0.06 – 0.13 | **<0.001** | 0.09 | 0.06 – 0.13 | **<0.001** |
| readstn100 | 0.02 | 0.01 – 0.03 | **<0.001** | 0.02 | 0.01 – 0.03 | **<0.001** | 0.02 | 0.01 – 0.03 | **<0.001** | 0.02 | 0.01 – 0.03 | **<0.001** | 0.02 | 0.01 – 0.03 | **<0.001** | 0.02 | 0.01 – 0.03 | **<0.001** |
| c60age × z p-tau217 | -0.01 | -0.01 – -0.00 | **<0.001** |  |  |  |  |  |  |  |  |  |  |  |  |  |  |  |
| z ab4240 |  |  |  | -0.00 | -0.07 – 0.07 | 0.969 |  |  |  |  |  |  |  |  |  |  |  |  |
| c60age × z ab4240 |  |  |  | 0.01 | 0.00 – 0.01 | **0.002** |  |  |  |  |  |  |  |  |  |  |  |  |
| z p-tau181 |  |  |  |  |  |  | -0.08 | -0.13 – -0.03 | **0.002** |  |  |  |  |  |  |  |  |  |
| z p-tau231 |  |  |  |  |  |  |  |  |  | -0.06 | -0.13 – -0.00 | **0.046** |  |  |  |  |  |  |
| z GFAP |  |  |  |  |  |  |  |  |  |  |  |  | -0.01 | -0.08 – 0.05 | 0.693 |  |  |  |
| c60age × z GFAP |  |  |  |  |  |  |  |  |  |  |  |  | -0.01 | -0.01 – -0.00 | **0.017** |  |  |  |
| z NfL |  |  |  |  |  |  |  |  |  |  |  |  |  |  |  | -0.02 | -0.10 – 0.06 | 0.553 |
| **Random Effects** | | | | | | | | | | | | | | | | | | |
| σ^2^ | 0.10 | | | 0.10 | | | 0.10 | | | 0.10 | | | 0.10 | | | 0.10 | | |
| τ_00_ | 0.50 _Reggieid_ | | | 0.50 _Reggieid_ | | | 0.47 _Reggieid_ | | | 0.49 _Reggieid_ | | | 0.50 _Reggieid_ | | | 0.50 _Reggieid_ | | |
| τ_11_ | 0.00 _Reggieid.c60age_ | | | 0.00 _Reggieid.c60age_ | | | 0.00 _Reggieid.c60age_ | | | 0.00 _Reggieid.c60age_ | | | 0.00 _Reggieid.c60age_ | | | 0.00 _Reggieid.c60age_ | | |
| ρ_01_ | 0.17 _Reggieid_ | | | 0.21 _Reggieid_ | | | 0.18 _Reggieid_ | | | 0.19 _Reggieid_ | | | 0.18 _Reggieid_ | | | 0.20 _Reggieid_ | | |
| ICC | 0.85 | | | 0.86 | | | 0.85 | | | 0.85 | | | 0.85 | | | 0.86 | | |
| N | 408 _Reggieid_ | | | 408 _Reggieid_ | | | 404 _Reggieid_ | | | 408 _Reggieid_ | | | 408 _Reggieid_ | | | 408 _Reggieid_ | | |
| Observations | 1690 | | | 1690 | | | 1671 | | | 1690 | | | 1690 | | | 1690 | | |
| Marginal R^2^ / Conditional R^2^ | 0.294 / 0.896 | | | 0.279 / 0.896 | | | 0.309 / 0.895 | | | 0.284 / 0.895 | | | 0.283 / 0.896 | | | 0.279 / 0.895 | | |
| AICc | 2336.4 | | | 2344.5 | | | 2309.2^+^ | | | 2347.6 | | | 2347.2 | | | 2351.2 | | |
| $\Delta$AICc | **-13.13** | | | -5.07 | | | -7.74 | | | -1.99 | | | -2.33 | | | 1.67 | | |

^*^ Base model includes the age fixed effects up to cubic polynomial (retaining highest order significant term and lower order terms; centered at age 60), random effects and covariates: sex, practice, education, WRAT3 reading score. $\Delta$AICc is calculated relative to the base model; negative numbers indicate better fit in the expanded model.

^+^There are 4 missing baseline p-tau181. The smallest AICc is Model 1, 2304.1 (ΔAICc=-12.92), followed by Model 3, 2309.2 (ΔAICc = -7.74), Model 2, 2311.8 (ΔAICc = -5.15) when we compared the model results in the same datasets.

**Supplementary Table 18: Immediate Memory mixed effects output.**

|  | **1(predictor= p-tau217)** | | | **2(predictor= Aβ42/40)** | | | **3(predictor= p-tau181)** | | | **4(predictor= p-tau231)** | | | **5(predictor= GFAP)** | | | **6(predictor= Nfl)** | | |
| --- | --- | --- | --- | --- | --- | --- | --- | --- | --- | --- | --- | --- | --- | --- | --- | --- | --- | --- |
| *Predictors* | *Estimates* | *CI* | *p* | *Estimates* | *CI* | *p* | *Estimates* | *CI* | *p* | *Estimates* | *CI* | *p* | *Estimates* | *CI* | *p* | *Estimates* | *CI* | *p* |
| (Intercept) | -0.17 | -0.37 – 0.02 | 0.078 | -0.20 | -0.40 – -0.01 | **0.037** | -0.17 | -0.37 – 0.02 | 0.081 | -0.19 | -0.38 – 0.01 | 0.056 | -0.18 | -0.38 – 0.02 | 0.072 | -0.16 | -0.36 – 0.03 | 0.105 |
| c60age | -0.04 | -0.06 – -0.03 | **<0.001** | -0.05 | -0.06 – -0.04 | **<0.001** | -0.05 | -0.06 – -0.03 | **<0.001** | -0.05 | -0.06 – -0.03 | **<0.001** | -0.05 | -0.06 – -0.04 | **<0.001** | -0.05 | -0.06 – -0.03 | **<0.001** |
| z p-tau217 | 0.02 | -0.03 – 0.06 | 0.417 |  |  |  |  |  |  |  |  |  |  |  |  |  |  |  |
| c60age^2 | -0.00 | -0.00 – 0.00 | 0.930 | -0.00 | -0.00 – 0.00 | 0.199 | -0.00 | -0.00 – 0.00 | 0.899 | -0.00 | -0.00 – 0.00 | 0.404 | -0.00 | -0.00 – 0.00 | 0.468 | -0.00 | -0.00 – 0.00 | 0.121 |
| c60age^3 | -0.00 | -0.00 – -0.00 | **0.007** | -0.00 | -0.00 – -0.00 | **0.003** | -0.00 | -0.00 – -0.00 | **<0.001** | -0.00 | -0.00 – -0.00 | **0.008** | -0.00 | -0.00 – -0.00 | **0.037** | -0.00 | -0.00 – -0.00 | **0.001** |
| gender f [Male] | -0.60 | -0.77 – -0.43 | **<0.001** | -0.61 | -0.78 – -0.44 | **<0.001** | -0.61 | -0.78 – -0.44 | **<0.001** | -0.61 | -0.78 – -0.44 | **<0.001** | -0.61 | -0.79 – -0.44 | **<0.001** | -0.62 | -0.79 – -0.45 | **<0.001** |
| BA f [no BA] | -0.24 | -0.43 – -0.05 | **0.013** | -0.22 | -0.41 – -0.03 | **0.023** | -0.25 | -0.44 – -0.05 | **0.012** | -0.23 | -0.42 – -0.04 | **0.019** | -0.23 | -0.42 – -0.03 | **0.021** | -0.24 | -0.43 – -0.05 | **0.015** |
| practice | 0.19 | 0.16 – 0.23 | **<0.001** | 0.20 | 0.16 – 0.24 | **<0.001** | 0.20 | 0.16 – 0.23 | **<0.001** | 0.20 | 0.16 – 0.23 | **<0.001** | 0.20 | 0.16 – 0.24 | **<0.001** | 0.19 | 0.15 – 0.23 | **<0.001** |
| readstn100 | 0.03 | 0.02 – 0.05 | **<0.001** | 0.04 | 0.02 – 0.05 | **<0.001** | 0.03 | 0.02 – 0.05 | **<0.001** | 0.03 | 0.02 – 0.05 | **<0.001** | 0.03 | 0.02 – 0.05 | **<0.001** | 0.03 | 0.02 – 0.05 | **<0.001** |
| c60age × z p-tau217 | -0.01 | -0.02 – -0.01 | **<0.001** |  |  |  |  |  |  |  |  |  |  |  |  |  |  |  |
| z p-tau217 × c60age^2 | -0.00 | -0.00 – -0.00 | **0.023** |  |  |  |  |  |  |  |  |  |  |  |  |  |  |  |
| z ab4240 |  |  |  | -0.03 | -0.11 – 0.05 | 0.424 |  |  |  |  |  |  |  |  |  |  |  |  |
| c60age × z ab4240 |  |  |  | 0.00 | -0.01 – 0.01 | 0.914 |  |  |  |  |  |  |  |  |  |  |  |  |
| z ab4240 × c60age^2 |  |  |  | 0.00 | 0.00 – 0.00 | **0.021** |  |  |  |  |  |  |  |  |  |  |  |  |
| z p-tau181 |  |  |  |  |  |  | 0.01 | -0.05 – 0.07 | 0.730 |  |  |  |  |  |  |  |  |  |
| c60age × z p-tau181 |  |  |  |  |  |  | -0.02 | -0.02 – -0.01 | **<0.001** |  |  |  |  |  |  |  |  |  |
| z p-tau231 |  |  |  |  |  |  |  |  |  | 0.02 | -0.05 – 0.09 | 0.544 |  |  |  |  |  |  |
| c60age × z p-tau231 |  |  |  |  |  |  |  |  |  | -0.01 | -0.01 – 0.00 | 0.066 |  |  |  |  |  |  |
| z p-tau231 × c60age^2 |  |  |  |  |  |  |  |  |  | -0.00 | -0.00 – -0.00 | **0.031** |  |  |  |  |  |  |
| z GFAP |  |  |  |  |  |  |  |  |  |  |  |  | 0.04 | -0.04 – 0.11 | 0.335 |  |  |  |
| c60age × z GFAP |  |  |  |  |  |  |  |  |  |  |  |  | -0.01 | -0.02 – 0.00 | 0.125 |  |  |  |
| z GFAP × c60age^2 |  |  |  |  |  |  |  |  |  |  |  |  | -0.00 | -0.00 – -0.00 | **0.030** |  |  |  |
| z NfL |  |  |  |  |  |  |  |  |  |  |  |  |  |  |  | -0.04 | -0.13 – 0.04 | 0.330 |
| **Random Effects** | | | | | | | | | | | | | | | | | | |
| σ^2^ | 0.24 | | | 0.24 | | | 0.24 | | | 0.24 | | | 0.24 | | | 0.24 | | |
| τ_00_ | 0.54 _Reggieid_ | | | 0.52 _Reggieid_ | | | 0.53 _Reggieid_ | | | 0.53 _Reggieid_ | | | 0.53 _Reggieid_ | | | 0.52 _Reggieid_ | | |
| τ_11_ | 0.00 _Reggieid.c60age_ | | | 0.00 _Reggieid.c60age_ | | | 0.00 _Reggieid.c60age_ | | | 0.00 _Reggieid.c60age_ | | | 0.00 _Reggieid.c60age_ | | | 0.00 _Reggieid.c60age_ | | |
| ρ_01_ | 0.05 _Reggieid_ | | | 0.06 _Reggieid_ | | | 0.02 _Reggieid_ | | | 0.03 _Reggieid_ | | | 0.03 _Reggieid_ | | | 0.01 _Reggieid_ | | |
| ICC | 0.73 | | | 0.74 | | | 0.73 | | | 0.74 | | | 0.73 | | | 0.74 | | |
| N | 412 _Reggieid_ | | | 412 _Reggieid_ | | | 408^+^ _Reggieid_ | | | 412 _Reggieid_ | | | 412 _Reggieid_ | | | 412 _Reggieid_ | | |
| Observations | 1822 | | | 1822 | | | 1802 | | | 1822 | | | 1822 | | | 1822 | | |
| Marginal R^2^ / Conditional R^2^ | 0.294 / 0.810 | | | 0.273 / 0.808 | | | 0.293 / 0.809 | | | 0.276 / 0.808 | | | 0.277 / 0.807 | | | 0.272 / 0.807 | | |
| AICc | 3713.2 | | | 3761.8 | | | 3693.7^+^ | | | 3753.6 | | | 3748.1 | | | 3764.1 | | |
| $\Delta$AICc | **-49.8** | | | -1.24 | | | -31.4 | | | -9.41 | | | -14.9 | | | 1.08 | | |

^*^ Base model includes the age fixed effects up to cubic polynomial (retaining highest order significant term and lower order terms; centered at age 60), random effects and covariates: sex, practice, education, WRAT3 reading score. $\Delta$AICc is calculated relative to the base model; negative numbers indicate better fit in the expanded model.

^+^There are 4 missing baseline p-tau181. The smallest AICc is Model 1, 3675.7 (ΔAICc=-49.4), followed by Model 3, 3693.7 (ΔAICc = -31.4), Model 5, 3710.4 (ΔAICc = -14.6), Model 4, 3715.7 (ΔAICc = -9.31) when we compared the model results in the same datasets.

**Supplementary Table 19: Delayed Memory mixed effects output.**

|  | **1(predictor= p-tau217)** | | | **2(predictor= Aβ42/40)** | | | **3(predictor= p-tau181)** | | | **4(predictor= p-tau231)** | | | **5(predictor= GFAP)** | | | **6(predictor= Nfl)** | | |
| --- | --- | --- | --- | --- | --- | --- | --- | --- | --- | --- | --- | --- | --- | --- | --- | --- | --- | --- |
| *Predictors* | *Estimates* | *CI* | *p* | *Estimates* | *CI* | *p* | *Estimates* | *CI* | *p* | *Estimates* | *CI* | *p* | *Estimates* | *CI* | *p* | *Estimates* | *CI* | *p* |
| (Intercept) | -0.09 | -0.29 – 0.10 | 0.340 | -0.12 | -0.31 – 0.08 | 0.236 | -0.08 | -0.28 – 0.11 | 0.392 | -0.11 | -0.30 – 0.08 | 0.259 | -0.10 | -0.30 – 0.10 | 0.325 | -0.09 | -0.29 – 0.11 | 0.373 |
| c60age | -0.03 | -0.05 – -0.02 | **<0.001** | -0.04 | -0.05 – -0.02 | **<0.001** | -0.04 | -0.05 – -0.02 | **<0.001** | -0.04 | -0.05 – -0.02 | **<0.001** | -0.04 | -0.05 – -0.02 | **<0.001** | -0.04 | -0.05 – -0.02 | **<0.001** |
| z p-tau217 | 0.02 | -0.03 – 0.06 | 0.438 |  |  |  |  |  |  |  |  |  |  |  |  |  |  |  |
| c60age^2 | -0.00 | -0.00 – 0.00 | 0.852 | -0.00 | -0.00 – 0.00 | 0.076 | -0.00 | -0.00 – 0.00 | 0.594 | -0.00 | -0.00 – 0.00 | 0.372 | -0.00 | -0.00 – 0.00 | 0.380 | -0.00 | -0.00 – 0.00 | 0.076 |
| c60age^3 | -0.00 | -0.00 – -0.00 | **0.005** | -0.00 | -0.00 – -0.00 | **0.001** | -0.00 | -0.00 – -0.00 | **0.001** | -0.00 | -0.00 – -0.00 | **0.006** | -0.00 | -0.00 – -0.00 | **0.040** | -0.00 | -0.00 – -0.00 | **0.001** |
| gender f [Male] | -0.54 | -0.71 – -0.37 | **<0.001** | -0.55 | -0.72 – -0.38 | **<0.001** | -0.54 | -0.71 – -0.37 | **<0.001** | -0.55 | -0.72 – -0.38 | **<0.001** | -0.56 | -0.73 – -0.39 | **<0.001** | -0.56 | -0.73 – -0.39 | **<0.001** |
| BA f [no BA] | -0.27 | -0.46 – -0.08 | **0.006** | -0.24 | -0.44 – -0.05 | **0.012** | -0.28 | -0.47 – -0.09 | **0.004** | -0.25 | -0.44 – -0.06 | **0.010** | -0.25 | -0.44 – -0.06 | **0.011** | -0.26 | -0.45 – -0.07 | **0.008** |
| practice | 0.17 | 0.13 – 0.21 | **<0.001** | 0.18 | 0.14 – 0.21 | **<0.001** | 0.17 | 0.14 – 0.21 | **<0.001** | 0.18 | 0.14 – 0.21 | **<0.001** | 0.17 | 0.14 – 0.21 | **<0.001** | 0.17 | 0.13 – 0.21 | **<0.001** |
| readstn100 | 0.03 | 0.02 – 0.04 | **<0.001** | 0.03 | 0.02 – 0.04 | **<0.001** | 0.03 | 0.02 – 0.04 | **<0.001** | 0.03 | 0.02 – 0.04 | **<0.001** | 0.03 | 0.02 – 0.04 | **<0.001** | 0.03 | 0.02 – 0.04 | **<0.001** |
| c60age × z p-tau217 | -0.01 | -0.02 – -0.01 | **<0.001** |  |  |  |  |  |  |  |  |  |  |  |  |  |  |  |
| z p-tau217 × c60age^2 | -0.00 | -0.00 – -0.00 | **0.034** |  |  |  |  |  |  |  |  |  |  |  |  |  |  |  |
| z ab4240 |  |  |  | 0.01 | -0.07 – 0.08 | 0.841 |  |  |  |  |  |  |  |  |  |  |  |  |
| z p-tau181 |  |  |  |  |  |  | -0.00 | -0.06 – 0.06 | 0.995 |  |  |  |  |  |  |  |  |  |
| c60age × z p-tau181 |  |  |  |  |  |  | -0.02 | -0.02 – -0.01 | **<0.001** |  |  |  |  |  |  |  |  |  |
| z p-tau231 |  |  |  |  |  |  |  |  |  | 0.02 | -0.05 – 0.09 | 0.574 |  |  |  |  |  |  |
| c60age × z p-tau231 |  |  |  |  |  |  |  |  |  | -0.01 | -0.02 – -0.00 | **0.017** |  |  |  |  |  |  |
| z p-tau231 × c60age^2 |  |  |  |  |  |  |  |  |  | -0.00 | -0.00 – -0.00 | **0.027** |  |  |  |  |  |  |
| z GFAP |  |  |  |  |  |  |  |  |  |  |  |  | 0.03 | -0.04 – 0.10 | 0.442 |  |  |  |
| c60age × z GFAP |  |  |  |  |  |  |  |  |  |  |  |  | -0.01 | -0.02 – 0.00 | 0.100 |  |  |  |
| z GFAP × c60age^2 |  |  |  |  |  |  |  |  |  |  |  |  | -0.00 | -0.00 – -0.00 | **0.014** |  |  |  |
| z NfL |  |  |  |  |  |  |  |  |  |  |  |  |  |  |  | -0.04 | -0.13 – 0.04 | 0.305 |
| **Random Effects** | | | | | | | | | | | | | | | | | | |
| σ^2^ | 0.22 | | | 0.22 | | | 0.22 | | | 0.22 | | | 0.22 | | | 0.22 | | |
| τ_00_ | 0.54 _Reggieid_ | | | 0.53 _Reggieid_ | | | 0.53 _Reggieid_ | | | 0.53 _Reggieid_ | | | 0.54 _Reggieid_ | | | 0.53 _Reggieid_ | | |
| τ_11_ | 0.00 _Reggieid.c60age_ | | | 0.00 _Reggieid.c60age_ | | | 0.00 _Reggieid.c60age_ | | | 0.00 _Reggieid.c60age_ | | | 0.00 _Reggieid.c60age_ | | | 0.00 _Reggieid.c60age_ | | |
| ρ_01_ | -0.09 _Reggieid_ | | | -0.11 _Reggieid_ | | | -0.13 _Reggieid_ | | | -0.11 _Reggieid_ | | | -0.10 _Reggieid_ | | | -0.12 _Reggieid_ | | |
| ICC | 0.75 | | | 0.76 | | | 0.75 | | | 0.76 | | | 0.76 | | | 0.76 | | |
| N | 412 _Reggieid_ | | | 412 _Reggieid_ | | | 408^+^ _Reggieid_ | | | 412 _Reggieid_ | | | 412 _Reggieid_ | | | 412 _Reggieid_ | | |
| Observations | 1820 | | | 1820 | | | 1800 | | | 1820 | | | 1820 | | | 1820 | | |
| Marginal R^2^ / Conditional R^2^ | 0.264 / 0.818 | | | 0.223 / 0.814 | | | 0.251 / 0.815 | | | 0.235 / 0.816 | | | 0.237 / 0.816 | | | 0.225 / 0.814 | | |
| AICc | 3638.0 | | | 3703.9 | | | 3636.4^+^ | | | 3687.1 | | | 3681.3 | | | 3702.9 | | |
| $\Delta$AICc | **-63.97** | | | 1.99 | | | -33.15 | | | -14.89 | | | -20.67 | | | 0.97 | | |

^*^ Base model includes the age fixed effects up to cubic polynomial (retaining highest order significant term and lower order terms; centered at age 60), random effects and covariates: sex, practice, education, WRAT3 reading score. $\Delta$AICc is calculated relative to the base model; negative numbers indicate better fit in the expanded model.

^+^There are 4 missing baseline p-tau181. The smallest AICc is Model 1, 3606.6 (ΔAICc=-63.01), followed by Model 3, 3636.4 (ΔAICc = -33.15), Model 5, 3649.2 (ΔAICc = -20.4), Model 4, 3655.1 (ΔAICc = -14.45) when we compared the model results in the same datasets.

**Supplementary Table 20: CDR Sum of Box mixed effects output.**

|  | **1(predictor= p-tau217)** | | | **2(predictor= Aβ42/40)** | | | **3(predictor= p-tau181)** | | | **4(predictor= p-tau231)** | | | **5(predictor= GFAP)** | | | **6(predictor= Nfl)** | | |
| --- | --- | --- | --- | --- | --- | --- | --- | --- | --- | --- | --- | --- | --- | --- | --- | --- | --- | --- |
| *Predictors* | *Estimates* | *CI* | *p* | *Estimates* | *CI* | *p* | *Estimates* | *CI* | *p* | *Estimates* | *CI* | *p* | *Estimates* | *CI* | *p* | *Estimates* | *CI* | *p* |
| (Intercept) | 0.00 | -0.12 – 0.12 | 0.981 | -0.05 | -0.12 – 0.02 | 0.159 | -0.04 | -0.11 – 0.03 | 0.292 | -0.03 | -0.11 – 0.04 | 0.341 | -0.04 | -0.11 – 0.04 | 0.327 | -0.02 | -0.09 – 0.05 | 0.588 |
| c60age | 0.00 | -0.01 – 0.01 | 0.691 | -0.00 | -0.01 – 0.01 | 0.821 | -0.00 | -0.01 – 0.01 | 0.803 | -0.00 | -0.01 – 0.01 | 0.702 | -0.00 | -0.01 – 0.01 | 0.661 | -0.01 | -0.02 – 0.00 | 0.203 |
| z p-tau217 | 0.01 | -0.02 – 0.03 | 0.695 |  |  |  |  |  |  |  |  |  |  |  |  |  |  |  |
| c60age^2 | 0.00 | -0.00 – 0.00 | 0.260 | 0.00 | 0.00 – 0.00 | **<0.001** | 0.00 | 0.00 – 0.00 | **0.041** | 0.00 | 0.00 – 0.00 | **0.041** | 0.00 | -0.00 – 0.00 | 0.067 | 0.00 | 0.00 – 0.00 | **0.001** |
| gender f [Male] | -0.01 | -0.08 – 0.06 | 0.788 | -0.01 | -0.08 – 0.06 | 0.773 | -0.01 | -0.08 – 0.06 | 0.836 | -0.00 | -0.07 – 0.07 | 0.936 | 0.01 | -0.06 – 0.08 | 0.792 | 0.00 | -0.07 – 0.07 | 0.957 |
| BA f [no BA] | 0.03 | -0.05 – 0.12 | 0.413 | 0.03 | -0.05 – 0.11 | 0.495 | 0.04 | -0.04 – 0.12 | 0.328 | 0.03 | -0.05 – 0.12 | 0.413 | 0.03 | -0.05 – 0.11 | 0.461 | 0.04 | -0.05 – 0.12 | 0.382 |
| practice | -0.01 | -0.04 – 0.01 | 0.424 |  |  |  |  |  |  |  |  |  |  |  |  |  |  |  |
| readstn100 | -0.01 | -0.01 – -0.00 | **0.004** | -0.01 | -0.01 – -0.00 | **0.004** | -0.01 | -0.01 – -0.00 | **0.009** | -0.01 | -0.01 – -0.00 | **0.005** | -0.01 | -0.01 – -0.00 | **0.006** | -0.01 | -0.01 – -0.00 | **0.004** |
| c60age × z p-tau217 | -0.01 | -0.01 – -0.00 | **0.037** |  |  |  |  |  |  |  |  |  |  |  |  |  |  |  |
| z p-tau217 × c60age^2 | 0.00 | 0.00 – 0.00 | **<0.001** |  |  |  |  |  |  |  |  |  |  |  |  |  |  |  |
| z ab4240 |  |  |  | -0.04 | -0.07 – -0.01 | **0.007** |  |  |  |  |  |  |  |  |  |  |  |  |
| z p-tau181 |  |  |  |  |  |  | -0.00 | -0.04 – 0.04 | 0.938 |  |  |  |  |  |  |  |  |  |
| c60age × z p-tau181 |  |  |  |  |  |  | -0.01 | -0.01 – 0.00 | 0.079 |  |  |  |  |  |  |  |  |  |
| z p-tau181 × c60age^2 |  |  |  |  |  |  | 0.00 | 0.00 – 0.00 | **<0.001** |  |  |  |  |  |  |  |  |  |
| z p-tau231 |  |  |  |  |  |  |  |  |  | -0.02 | -0.05 – 0.02 | 0.419 |  |  |  |  |  |  |
| c60age × z p-tau231 |  |  |  |  |  |  |  |  |  | -0.00 | -0.01 – 0.00 | 0.302 |  |  |  |  |  |  |
| z p-tau231 × c60age^2 |  |  |  |  |  |  |  |  |  | 0.00 | 0.00 – 0.00 | **<0.001** |  |  |  |  |  |  |
| z GFAP |  |  |  |  |  |  |  |  |  |  |  |  | 0.00 | -0.05 – 0.05 | 0.973 |  |  |  |
| c60age × z GFAP |  |  |  |  |  |  |  |  |  |  |  |  | -0.00 | -0.01 – 0.00 | 0.256 |  |  |  |
| z GFAP × c60age^2 |  |  |  |  |  |  |  |  |  |  |  |  | 0.00 | 0.00 – 0.00 | **<0.001** |  |  |  |
| z NfL |  |  |  |  |  |  |  |  |  |  |  |  |  |  |  | 0.05 | 0.00 – 0.11 | **0.048** |
| c60age × z NfL |  |  |  |  |  |  |  |  |  |  |  |  |  |  |  | -0.01 | -0.02 – 0.00 | 0.081 |
| z NfL × c60age^2 |  |  |  |  |  |  |  |  |  |  |  |  |  |  |  | 0.00 | 0.00 – 0.00 | **0.010** |
| **Random Effects** | | | | | | | | | | | | | | | | | | |
| σ^2^ | 0.17 | | | 0.18 | | | 0.18 | | | 0.17 | | | 0.18 | | | 0.18 | | |
| τ_00_ | 0.02 _Reggieid_ | | | 0.01 _Reggieid_ | | | 0.02 _Reggieid_ | | | 0.02 _Reggieid_ | | | 0.01 _Reggieid_ | | | 0.01 _Reggieid_ | | |
| τ_11_ | 0.00 _Reggieid.c60age_ | | | 0.00 _Reggieid.c60age_ | | | 0.00 _Reggieid.c60age_ | | | 0.00 _Reggieid.c60age_ | | | 0.00 _Reggieid.c60age_ | | | 0.00 _Reggieid.c60age_ | | |
| ρ_01_ | -0.54 _Reggieid_ | | | -0.77 _Reggieid_ | | | -0.60 _Reggieid_ | | | -0.60 _Reggieid_ | | | -0.62 _Reggieid_ | | | -0.70 _Reggieid_ | | |
| ICC | 0.39 | | | 0.37 | | | 0.38 | | | 0.38 | | | 0.38 | | | 0.38 | | |
| N | 404 _Reggieid_ | | | 404 _Reggieid_ | | | 400^+^ _Reggieid_ | | | 404 _Reggieid_ | | | 404 _Reggieid_ | | | 404 _Reggieid_ | | |
| Observations | 1243 | | | 1243 | | | 1230 | | | 1243 | | | 1243 | | | 1243 | | |
| Marginal R^2^ / Conditional R^2^ | 0.162 / 0.486 | | | 0.054 / 0.407 | | | 0.106 / 0.449 | | | 0.121 / 0.459 | | | 0.108 / 0.445 | | | 0.071 / 0.420 | | |
| AICc | 1721.8 | | | 1765.2 | | | 1739.6^+^ | | | 1739.9 | | | 1746.5 | | | 1764.8 | | |
| $\Delta$AICc | **-48.67** | | | -5.26 | | | -23.85 | | | -30.64 | | | -23.98 | | | -5.69 | | |

^*^ Base model includes the age fixed effects up to cubic polynomial (retaining highest order significant term and lower order terms; centered at age 60), random effects and covariates: sex, education, WRAT3 reading score. $\Delta$AICc is calculated relative to the base model; negative numbers indicate better fit in the expanded model.

^+^There are 4 missing baseline p-tau181. The smallest AICc is Model 1, 1715.6 (ΔAICc=-47.85), followed by Model 4, 1732.8 (ΔAICc = -30.58), Model 5, 1739.5 (ΔAICc = -23.97), Model 3, 1739.6 (ΔAICc = -23.85), Model 6, 1757.2 (ΔAICc = -6.18), Model 2, 1758.2 (ΔAICc = -5.20) when we compared the model results in the same datasets.
